# Pseudo-chromosome–length genome assembly of a double haploid “Bartlett” pear (*Pyrus communis* L.)

**DOI:** 10.1093/gigascience/giz138

**Published:** 2019-12-09

**Authors:** Gareth Linsmith, Stephane Rombauts, Sara Montanari, Cecilia H Deng, Jean-Marc Celton, Philippe Guérif, Chang Liu, Rolf Lohaus, Jason D Zurn, Alessandro Cestaro, Nahla V Bassil, Linda V Bakker, Elio Schijlen, Susan E Gardiner, Yves Lespinasse, Charles-Eric Durel, Riccardo Velasco, David B Neale, David Chagné, Yves Van de Peer, Michela Troggio, Luca Bianco

**Affiliations:** 1 Center for Plant Systems Biology, VIB, Technologiepark 71, 9052, Gent, Belgium; 2 Department of Plant Biotechnology and Bioinformatics, Ghent University, Technologiepark 71, 9052 Gent, Belgium; 3 Fondazione Edmund Mach, via E. Mach 1, 38010, San Michele all'Adige (TN), Italy; 4 University of California Davis, Department of Plant Sciences, One Shields Ave, Davis, CA 95616, USA; 5 The New Zealand Institute for Plant & Food Research Limited (PFR), Mt Albert Research Centre,120 Mt Albert Road, Sandringham, Auckland, 1025, New Zealand; 6 IRHS, INRA, Agrocampus-Ouest, Université d'Angers, SFR 4207 Quasav, 42 rue Georges Morel, F-49071 Beaucouzé, France; 7 ZMBP, Allgemeine Genetik, Universität Tübingen, Auf der Morgenstelle 32, D-72076 Tübingen, Germany; 8 USDA-ARS National Clonal Germplasm Repository, 33447 Peoria Road, Corvallis, OR 97333, USA; 9 Wageningen UR – Bioscience P.O. Box 16, 6700AA, Wageningen, The Netherlands; 10 The New Zealand Institute for Plant & Food Research Limited (PFR), Palmerston North Research Centre, Palmerston North, New Zealand; 11 CREA Research Centre for Viticulture and Enology, Via XXVIII Aprile 26, 31015 Conegliano (TV), Italy; 12 Center for Microbial Ecology and Genomics, Department of Biochemistry, Genetics and Microbiology, University of Pretoria, Roper street, Pretoria 0028, South Africa

**Keywords:** Pyrus communis L, chromosome-scale assembly, Hi-C, Pac-Bio sequencing

## Abstract

**Background:**

We report an improved assembly and scaffolding of the European pear (*Pyrus communis* L.) genome (referred to as BartlettDHv2.0), obtained using a combination of Pacific Biosciences RSII long-read sequencing, Bionano optical mapping, chromatin interaction capture (Hi-C), and genetic mapping. The sample selected for sequencing is a double haploid derived from the same “Bartlett” reference pear that was previously sequenced. Sequencing of di-haploid plants makes assembly more tractable in highly heterozygous species such as *P. communis*.

**Findings:**

A total of 496.9 Mb corresponding to 97% of the estimated genome size were assembled into 494 scaffolds. Hi-C data and a high-density genetic map allowed us to anchor and orient 87% of the sequence on the 17 pear chromosomes. Approximately 50% (247 Mb) of the genome consists of repetitive sequences. Gene annotation confirmed the presence of 37,445 protein-coding genes, which is 13% fewer than previously predicted.

**Conclusions:**

We showed that the use of a doubled-haploid plant is an effective solution to the problems presented by high levels of heterozygosity and duplication for the generation of high-quality genome assemblies. We present a high-quality chromosome-scale assembly of the European pear *Pyrus communis* and demostrate its high degree of synteny with the genomes of *Malus x Domestica* and *Pyrus x bretschneideri*.

## Introduction

The genomics era has revolutionized research on fruit tree species, and many of these genomes have recently been sequenced or are currently being sequenced [1, 2]. Nevertheless, although the cost for sequencing genomes has decreased considerably, obtaining high-quality assemblies and annotations for complex plant genomes is still challenging [3]. In addition to high numbers of repeats and transposable elements, high levels of heterozygosity complicate genome assembly for most fruit trees. Indeed, outcrossing fruit tree species often exhibit extremely high levels of heterozygosity with, e.g., in apple [4], 1 single-nucleotide polymorphism (SNP) every 50 bp. The traditional solution to circumvent the challenge of heterozygosity is to sequence highly inbred plant material [5, 6]. However, such material may not always be available and many sequencing projects have used heterozygous samples for sequencing of economically important cultivars [7, 8].

Earlier assemblies of Asian pear (*Pyrus*×*bretschneideri*) [8], European pear (*Pyrus communis*) [9], and apple (*Malus*×*domestica*) [7] were based on heterozygous plant material, resulting in each case in erroneous and fragmented assemblies consisting of thousands of scaffolds. Both the Asian pear and apple genomes were subsequently re-assembled using different strategies to address the problem of extreme heterozygosity [2, 8]. In the case of Asian pear the genome was re-assembled using a BAC by BAC strategy, combined with Illumina sequencing [8]. For apple, a double-haploid (DH) plant, derived from the same cultivar “Golden Delicious” as the original reference genome, was sequenced [2].

Here, we describe the assembly of the genome of the European pear (*P. communis*) using a DH derived from the variety “Bartlett,” analogous to the strategy used by Daccord et al. [2] in apple. The “Bartlett.DH” developed at INRA, Angers, France [10], was chosen because it is derived from the same cultivar as used for the previous European pear assembly, Bartlettv1.0, obtained by Roche 454 sequencing of extremely heterozygous plant material [9]. This new genome sequence (BartlettDHv2.0) was assembled by combining short-read Illumina and long-read PacBio sequencing, optical mapping, Hi-C, and genetic maps. The BartlettDHv2.0 genome assembly improves the European pear assembly to 17 pseudo-chromosomes and will be a critical tool for contemporary genomic studies in pear, including genome-wide association studies (GWAS) and GS for the benefit of pear breeding. There currently exist resequencing data from 113 pear accessions [11] representing both cultivated and wild pear species, but as yet no chromosome-scale reference genome has been published.

## Results and Discussion

### Genome sequencing and assembly

**Figure 1:**
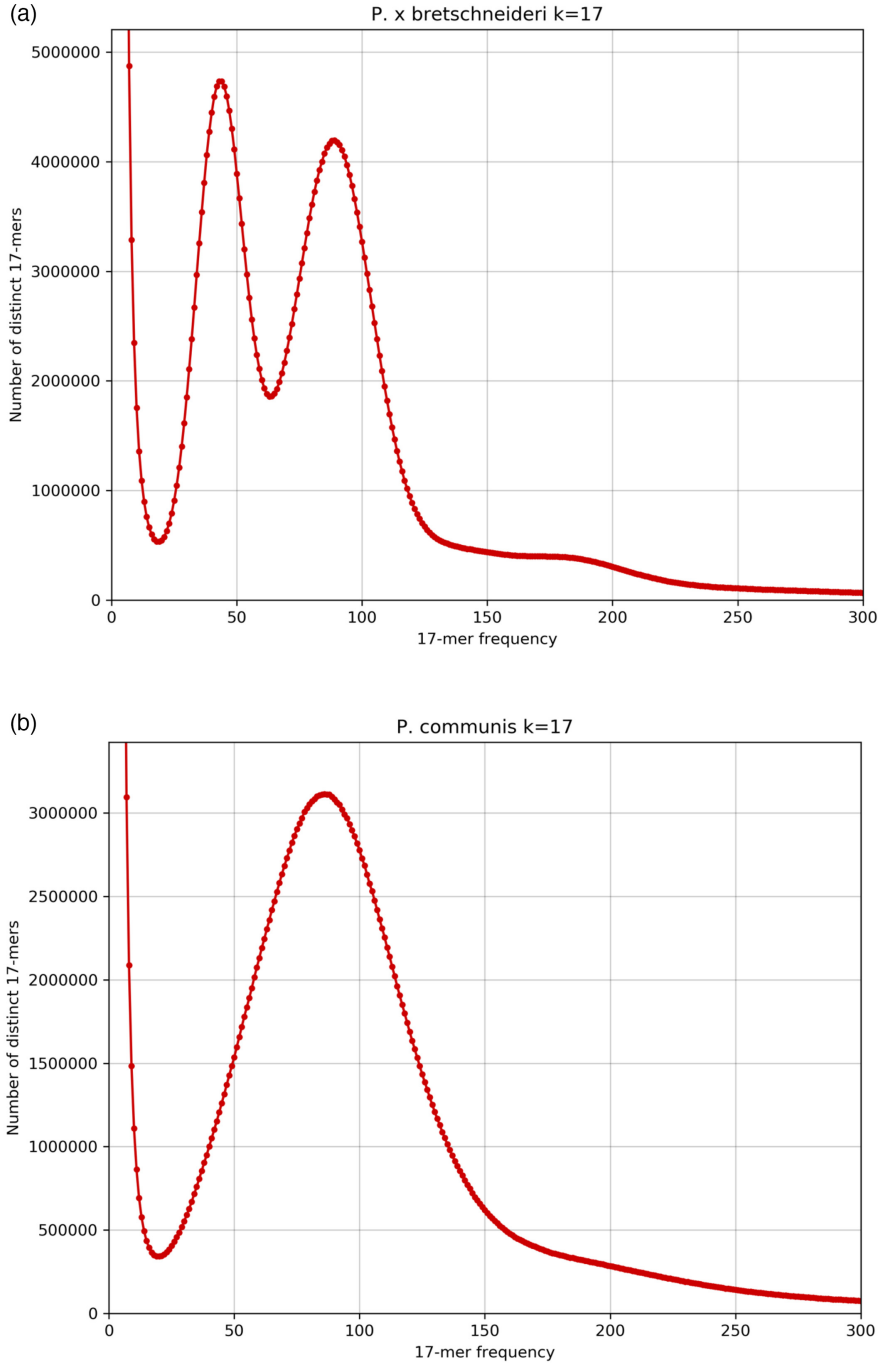
(a) 17-mer frequency distribution of diploid *P*× *bretschneideri*. Using KAT [13] v2.3.4, 17-mers were counted in all whole-genome shotgun PE reads. The density plot of the number of unique *k*-mer species (y axis) for each *k*-mer frequency (x axis) is plotted. The homozygous peak is observed at a multiplicity (*k*-mer coverage) of 86×, while the heterozygous peak is observed at 43×. (b) 17-mer frequency distribution of DH *P. communis*(BartlettDHv2.0). Using KAT [13] v2.3.4, 17-mers were counted in all whole-genome shotgun PE reads. The density plot of the number of unique *k*-mer species (y axis) for each *k*-mer frequency (x axis) is plotted. The homozygous peak is observed at a multiplicity (*k*-mer coverage) of 86×, while no heterozygous peak is observed.

A total of 31.4 Gb of PacBio RSII long-read sequence data were produced, comprising 3,665,270 reads with a read N50 of 14.2 kb. Reads longer than 10 kb sum to 21.9 Gb. The RSII sequencing was supplemented by 123-fold coverage of Illumina (2 × 125 bp) paired-end (PE) reads with a target insert size of 350 bp (61.5 Gb of sequence). Sequencing of 2 Hi-C libraries yielded 51.6 Gb of Illumina PE data as (2 × 125 bp) reads. *k*-mer analysis of PE Illumina data confirmed the homozygous nature of the “Bartlett.DH” sample, with no heterozygosity peak visible in the 17-mer frequency distribution (Fig. 1b vs Fig. 1a for Asian pear). Estimation of genome size from the 17-mer distribution provided an estimate of 528 Mb, which agrees well with the 527 Mb genome size estimation made by Wu et al. [8] for Asian pear. The PacBio data therefore equate to 63-fold, long-read coverage of the genome with 44-fold coverage in reads >10 kb.

The genome was assembled into 592 scaffolds totalling 496.9 Mb, or 94.0% of the expected genome size. The scaffold N50 is 6.5 Mb, which is a near 1,000-fold improvement over the Bartlettv1.0 assembly. Of these assembled scaffolds, 230 scaffolds totalling 445.1 Mb could be anchored to the 17 chromosomes of the pear genome using a combination of Hi-C data and the high-density genetic map. Thus 84.2% of the genome is anchored into 17 pseudomolecules with a further 51.8 Mb (477 smaller sequences) collected in linkage group zero (LG0). These metrics are summarized in Table 1. Searching of telomeric sequences (5′-TTTAGGG-3′) enriched in the terminal parts of the pseudo-chromosomes allowed the identification of 22 telomeres. Of the 17 pseudo-chromosomes, all but Chr14 and Chr16 had ≥1 of the 2 telomeres, and the presence of telomeric sequences could be found at both ends of 7 of the pseudo-chromosomes (Chr4, Chr6, Chr8, Chr10, Chr11, Chr15, and Chr17). Additionally, SuperScaffold_290 was found to start with the 3′-CCCTAAA-5′ sequence, suggesting that it may be located at the terminal end of 1 chromosome. This hypothesis is also supported by 3 SNPs from this scaffold genetically mapping at the beginning of Chr02.

BUSCO analysis revealed 1,357 complete BUSCOs (94.3%, 64.0% single copy and 30.3% duplicated) with 1.9% fragmented and 3.8% missing BUSCOs. Marey maps [12], showing the relationship between genetic and physical distance across each chromosome, demonstrate good agreement between the assembly and the Bartlett genetic map (Supplementary Figs S1–S17); an example showing Chr1 is provided in Fig. 2. All plots have been produced using Python's matplotlib v.3.0.3 library.

**Figure 2:**
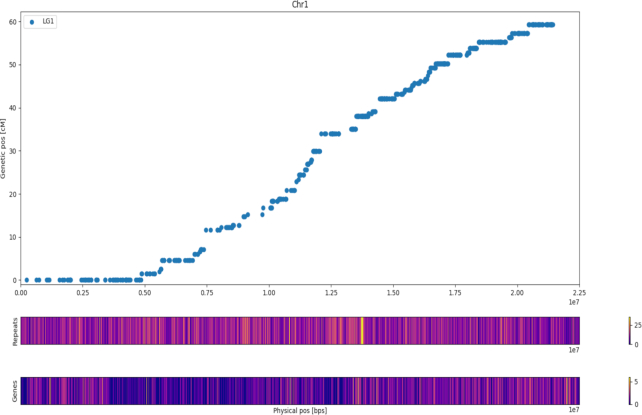
Marey plot of Chr1 with heat map of dispersed repeats and genes in bins of 200 kb. The lighter the colour the more elements are present. Genetic positions refer to the high-density map of Bartlett. Dots represent the genetic and physic position (on BartlettDHv2.0) of 11,474 SNPs.

**Table 1:** Genome assembly metrics

Metric	Total assembled (Mb)	% Genome	N50 (MB)	No. of sequences
Contigs	501	94.8	5.3	620
Scaffolds	496.9	94.0	6.5	592
Anchored into chromosomes	445.1	84.2	26.2	17
LG0	51.8	9.8	0.19	477

Using the high-density genetic map of Bartlett, a haplotype map for BartlettDHv2 was produced, allowing the identification of the 2 haplotypes of Bartlett in the BartlettDHv2 assembly, as well as the recombination breakpoints (Supplementary Fig. S1), confirming the gynogenesis origin of the BartlettDHv2 hypothesized by Bouvier et al. [10].

Summary statistics of 2 assemblies produced using Canu [14] and Falcon [15] are presented in Table 2. The Canu assembly has higher contiguity (501 Mb in 620 scaffolds), while the Falcon assembly produces a slightly larger but more fragmented result (515 Mb in 1,282 scaffolds). Both assemblies were used for the optical mapping data analysis, and results for both the Canu and Falcon assemblies are provided in Table 3. While the total amount of sequence is similar in both cases, the Canu assembly produced fewer conflicts with the optical mapping data than Falcon (13 vs 38), as well as much longer scaffolds (scaffold N50 of 8.1 Mb vs 3.5 Mb in Canu and Falcon, respectively). Alignment with the high-density linkage map indicated that the Canu assembly produced fewer conflicts with the genetic map than the Falcon assembly (3 vs 8). The Canu assembly was therefore selected as the contig assembly.

**Table 2:** Summary statistics of best Canu and best Falcon contig assemblies

Assembly	Total assembled (Mb)	% Genome	N50 (Mb)	No. of contigs	>140 kb (Mb)
Canu	501	94.8	5.3	620	479.6
Falcon	515	97.5	2.4	1,282	483.6

**Table 3:** Summary statistics of the Canu and Falcon hybrid assemblies combined with the Bionano optical mapping data

Hybrid assembly	Bionano incorporated (Mb)	% Genome	N50 (Mb)	No. of scaffolds	No. of conflicts with optical map
Canu + Bionano	459.2	87.0	8.1	123	13
Falcon + Bionano	451.4	85.4	3.5	214	38

Consensus was called on the assembly using PacBio whole-genome sequencing (WGS), Illumina WGS, and Illumina RNA-Seq data. A single iteration of consensus calling using raw PacBio data was followed by polishing with Illumina WGS data. This Illumina consensus calling was performed iteratively while monitoring the number of *k*-mers shared between the assembly and the Illumina read data. This metric reached a maximum value after 7 iterations and Illumina WGS consensus calling was halted at this point. Finally, iterative consensus calling was run using RNA sequencing (RNA-Seq) data instead of the WGS Illumina data in order to focus the consensus on coding sequence (CDS). The rationale for this was that small errors are particularly a problem in coding regions because they can introduce frameshifts that severely affect the annotation of genes [16]. Metrics indicated that the consensus calling of coding regions was optimal after the second iteration. The second iteration of RNA-Seq consensus calling was therefore selected as the final scaffold assembly. *k*-mer comparison of the assembly with Illumina data (Supplementary Fig. S37) does not suggest any significant amount of duplicated or missing content.

Combining scaffolds with proximity information from Hi-C sequencing enabled arrangement of the scaffolds into 17 ordered and oriented clusters representing the 17 chromosomes of the pear genome. Agreement of Hi-C clusters with the genetic map was not perfect but was very high, with 11 of the 17 Hi-C clusters being in perfect agreement with the genetic map. For such clusters, every anchored scaffold in that cluster is anchored to the same linkage group (LG) by the genetic map and no scaffold from another cluster was ever anchored to that LG. Comparison of the other 6 Hi-C clusters with the genetic map suggested that the Hi-C had correctly grouped and oriented chromosome arms. These clusters could be made to agree perfectly with the genetic map by splitting each of them into 2. These remaining 6 clusters were therefore split and then rejoined in accordance with the genetic map.

### Comparison of BartlettDHv2.0 assembly with Bartlettv1.0 assembly

The Bartlettv1.0 assembly totals 507.7 Mb (excluding N's), of which 99.8% (506.8 Mb of sequence in 141,034 of the 142,083 original scaffolds) was aligned to the BartlettDHv2.0 assembly. Inter-assembly synteny is very strong, suggesting that although highly fragmented, the Bartlettv1.0 assembly was a true depiction of the genome. There is evidence of some haplotype separation in the Bartlettv1.0 assembly because 25,120 scaffolds totalling 25.6 Mb align to overlapping positions on the BartlettDHv2.0 assembly. Conversely, 1,974 scaffolds, totalling 1.6 Mb, aligned to multiple places in the BartlettDHv2.0 assembly. These scaffolds represent repeats that are collapsed in the Bartlettv1.0 assembly but not in the BartlettDHv2.0 assembly. This 1.6 Mb of repeat scaffolds from the Bartlettv1.0 assembly becomes 4.4 Mb of sequence in the BartlettDHv2.0 assembly, highlighting the importance of third-generation, long-read data in resolving the repetitive structures of plant genomes.

### Gene annotation

The combination of *ab initio* gene prediction with protein alignment and complementary DNA alignment prediction enabled the annotation of 37,445 protein-coding genes in the BartlettDHv2.0 assembly. BUSCO analysis of predicted proteins revealed uncovering of 1,202 complete “BUSCO” genes (83.5%, 62.7% single copy and 20.8% duplicated) with 6.2% fragmented and 10.3% missing BUSCO genes. In total, 95% of these are supported by RNA-seq evidence. On average, gene models consisted of transcript lengths of 2,944 bp, coding lengths of 1,120 bp, and 5 exons per gene." to match the values in the table. These values are similar to those observed in Asian pear [8], apple [2], and the Bartlettv1.0 assembly [9] (Table 4). All gene models had matches in ≥1 of the public protein databases (nrprot or interpro), while 95% of them contained domains recognized in the interpro database. The average gene density in BartlettDHv2.0 assembly is 7.1 genes per 100 kb, with genes being more abundant in sub-telomeric regions, as previously observed in other sequenced plant genomes (Fig. 2, Supplementary Figs S1–S17).

**Table 4:** Summary statistics of gene annotations from selected Rosaceae species, *Pyrus communis*(BartlettDHv2.0, Bartlettv1.0 assembly [9]), *Pyrus*×*bretschneideri* [8], *Malus*×*domestica* (GDDH13) [2], *Fragaria vesca* [17]

Statistic	*P. communis* (BartlettDHv2.0)	*P. communis* (Bartlettv1.0)	*P*. × *bretschneideri*	*F. vesca*	*M*. × *domestica*(GDDH13)
Predicted genes	37,445	45,217	42,812	28,588	44,105
Mean CDS length (nt)	1,120	1,209	1,346	1,177	1,167
Mean exon length (CDS only) (nt)	222	236	285	297	282
Mean intron length (nt)	296	352	346	407	689
Mean exons per gene	5	5	5	5	5
Single-exon genes	6,789	11,268	12,309	5,004	6,350
Genes per 100 kb	7.6	9.1	8.5	62.3	6.7

### Orthology analysis

**Figure 3:**
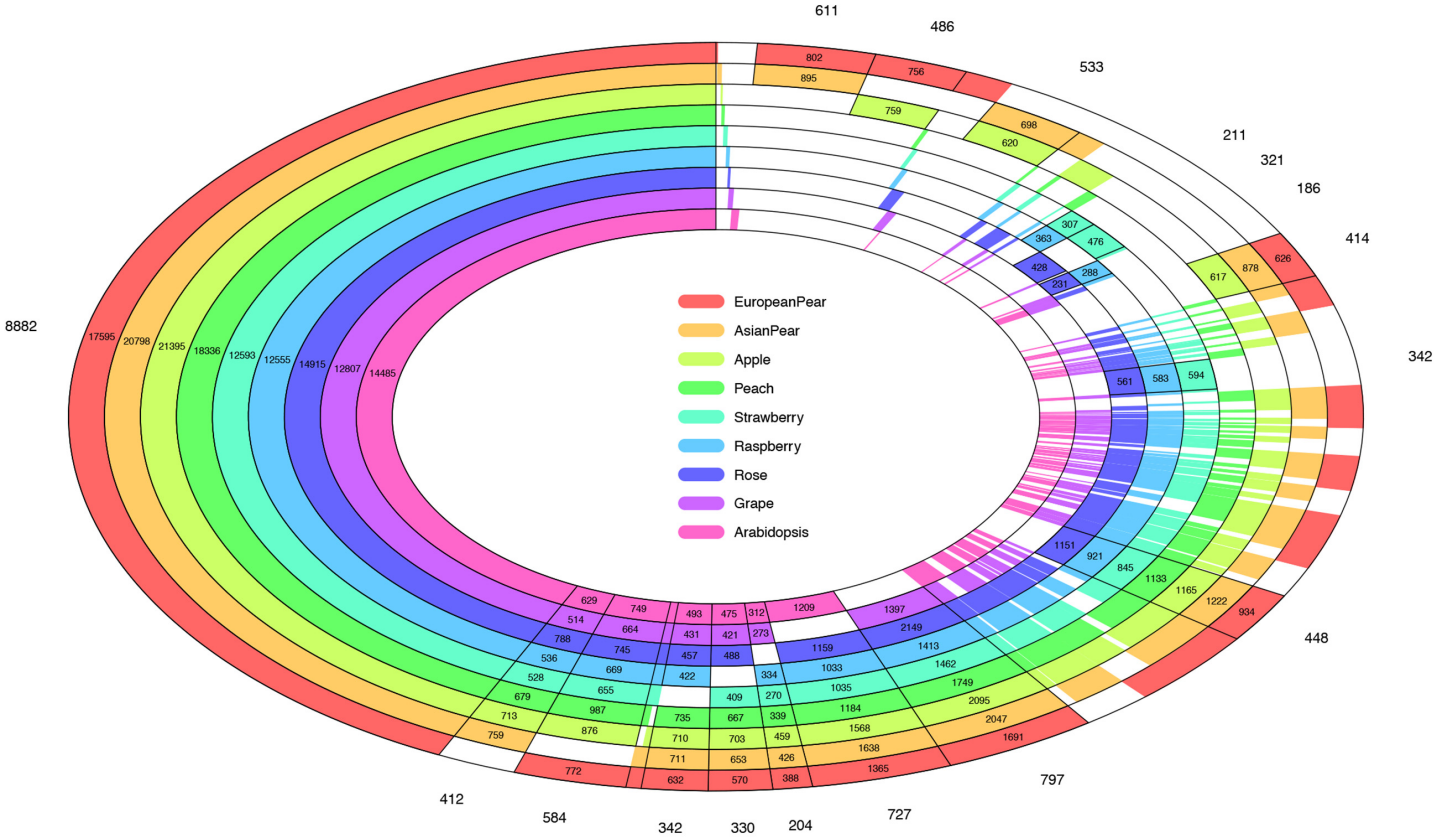
Plot of protein clusters shared by 9 species, *P*.×*bretschneideri* [8], *P. communis*(BartlettDHv2.0), *M*.×*domestica* [2], *F. vesca* [17], *P. persica* [18], *R. chinensis* [19], *R. occidentalis* [1], *V. vinifera* [20], and *A. thaliana* [21].

The predicted protein sequences from European pear were compared with those from 8 other species, *Pyrus*×*bretschneideri* [8], *Malus*×*domestica* (GDDH13) [2], *Fragaria vesca* [17], *Prunus persica* [18], *Rosa chinensis* [19], *Rubus occidentalis* [1], *Vitis vinifera* [20], and *Arabidopsis thaliana* [21]. Proteins were clustered into 20,677 orthologous groups (≥2 members), of which 8,877 (43%) were common to all 9 genomes (Fig. 3). Full results of the orthology analysis are available from the pear project database on request. A set of 414 gene clusters were identified as being specific to the 3 pome fruits analysed (i.e., to apple and the 2 species of pear). A set of 611 gene clusters was identified as being shared by the 2 pear species but not by apple. A set of 8 gene clusters was found to be specific to the European pear, while 22 gene clusters were specific to the Asian pear and 7 gene clusters were found to be specific to apple.

Gene clusters that were determined by the orthology analysis to be pear specific, or specific to 1 of the 3 Malinae species (Asian pear, European pear, and apple), were queried in the other Malinae genomes by aligning gene sequences with Genome Threader [22]. This gene sequence re-alignment revealed that, in most of these cases, gene clusters shown to be organism specific by the orthology analysis revealed genes that were missed by the automatic annotation of the respective genome assemblies. All Asian pear and European pear–specific gene clusters could be identified in 1 of the other Malinae genomes, while 5 gene clusters were found to be genuinely apple specific. Of the 611 pear-specific gene clusters, 526 were found in the apple genome. Of the remaining 85 pear-specific gene clusters 74 are supported by RNA-Seq in *P. communis*, 31 have a functional annotation, and all 85 have either RNA-Seq support or a functional annotation. The gene structures resolved by alignment of Asian pear and apple genes were merged with the BartlettDHv2.0 annotation, adding a further 209 gene models.

The results of this gene structure re-alignment highlight the limits of automated gene annotation and the importance of ongoing curation of gene structure annotations. An example of the importance of manual curation of gene models has recently been reported in kiwifruit, where >90% of the *in silico* predicted gene models were re-annotated compared to a previous draft version [23]. The annotation of the BartlettDHv2.0 assembly has been loaded into the Online Resource for Community Annotation of Eukaryotes (ORCAE) [24] to facilitate ongoing manual curation of gene models.

### Whole-genome duplication

**Figure 4:**
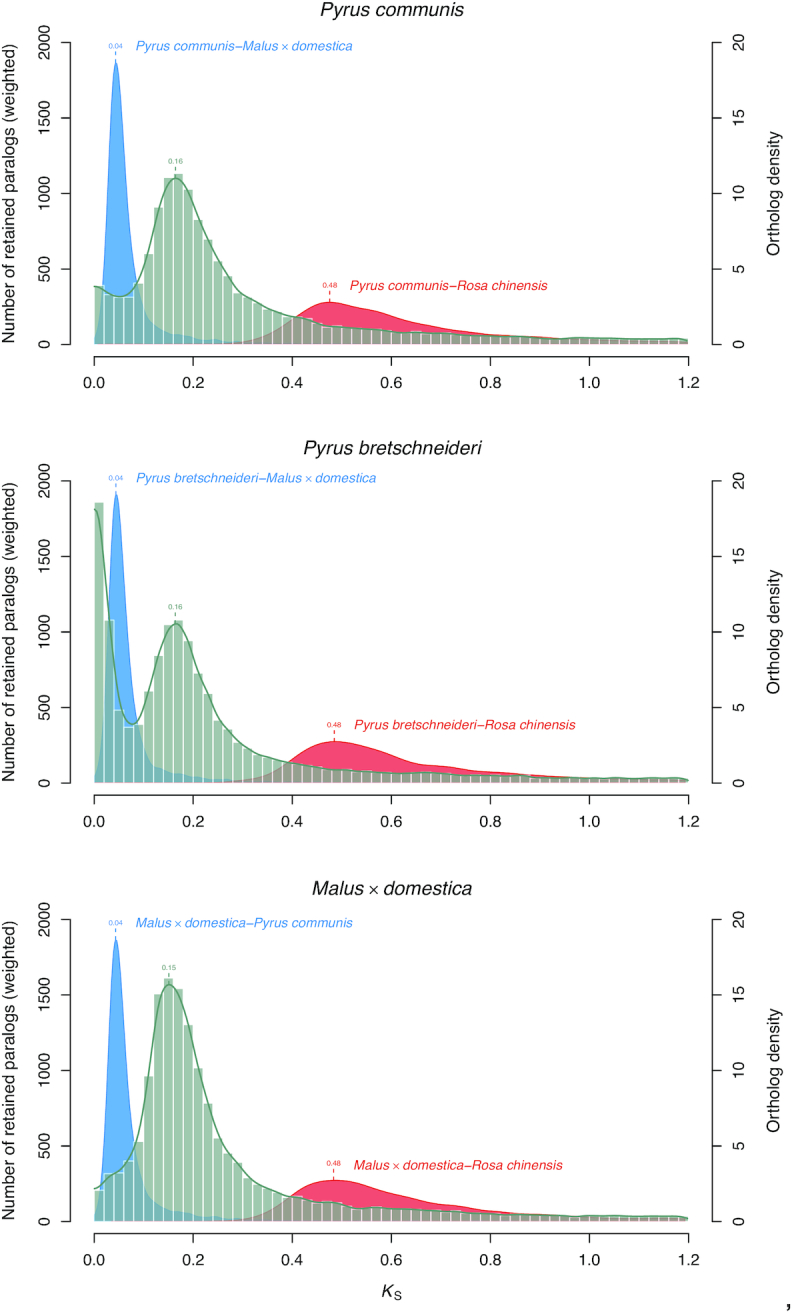
(a, b, c) Paralog *K*_S_ distributions of *P. communis*BartlettDHv2.0, *P*. × *bretschneideri* [8], and *M*. × *domestica* GDDH13 [2] (grey histograms and line, left-hand y-axes; a peak represents a WGD event) and 1-to-1 ortholog *K*_S_ distributions between indicated species (blue and red filled curves of kernel-density estimates, right-hand y-axes; a peak represents a species divergence event).

Distributions of synonymous substitutions per synonymous site (*K*_S_) produced for the whole paranomes of *P. communis, P*. × *bretschneideri*, and *M*. × *domestica* all support the common whole-genome duplication (WGD) event shared by the Malinae. Signature WGD peaks in the *K*_S_ plots for the 3 species can be found at almost identical *K*_S_ values of ∼0.16 (Fig. 4 a, b, c), as expected on the basis of previous research [2, 7–9]. Comparison of these WGD *K*_S_ peaks with the *K*_S_ peaks of ortholog distributions between pears and apple and between pears/apple and rose (*R. chinensis*) [19] suggest that the WGD occurred quite a long time after the divergence of Amygdaloideae and Rosoideae and well before the divergence of pear and apple (unless substantial substitution rate acceleration/deceleration occurred in these lineages).

### Functional annotation and GO enrichment analysis

A combination of BLAST (NR prot) and interproscan searches enabled the annotation of 12,444 of the 37,445 genes (33%) with a functional description. Loading predicted transcripts into the TRAPID online annotation platform [25] enabled annotation of 24,257 (69%) genes with ≥1 GO term. GO enrichment analysis was performed within the TRAPID platform on gene sets of particular biological interest, i.e., pear-specific gene families and pome-specific gene families. No enriched GO terms were found for the pear-specific gene families, while significantly enriched GO terms for the pome-specific gene families are presented in the supplementary material (Table S1).

### Repetitive element annotation

A combination of *de novo* and homology-based repeat annotation identified a total of 247 Mb of transposable element sequences, accounting for 49.7% of the assembly. As is typical for plant genomes, the most abundant transposable elements are retrotransposons of the long terminal repeat (LTR) family, totalling 32.6% of the genome. Although widely dispersed throughout the genome, transposon-related sequences were most abundant in centromeric regions.

The recent re-assembly of the apple genome [2] revealed a previously undescribed LTR element dubbed “HODOR” (high-copy golden delicious repeat), and the expansion of this element was implicated as having a potential role in the speciation of apple and pear. This element has now been verified in the pear genome. BLAST analysis revealed 232 full-length HODOR copies in the BartlettDHv2.0 genome, only 29% of the number of full-length copies identified in the apple genome. Although the HODOR element has, to date, only been identified in the apple and pear genomes, this finding must be treated with a degree of caution. The apple and pear genomes have been re-assembled using the latest long-read technology to arrive at chromosome-scale assemblies. HODOR is a 9.2-kb transposable element, and as such it may simply not have been completely assembled in previous Rosaceae genomes based on short-read data. Nevertheless, BLAST searches reveal no trace of the HODOR element in the recent chromosome-scale re-assemblies of *F. vesca* [17], *R. chinensis* [19], or *R. occidentalis* [1], all of which were developed from long-read data.

Future in-depth studies into the repeat content of Rosaceae genomes may reveal the point in the evolution of the Rosaceae at which this element first emerged and how it relates to phenotypic differences among Rosaceae species.

### Chromosome structure

**Figure 5:**
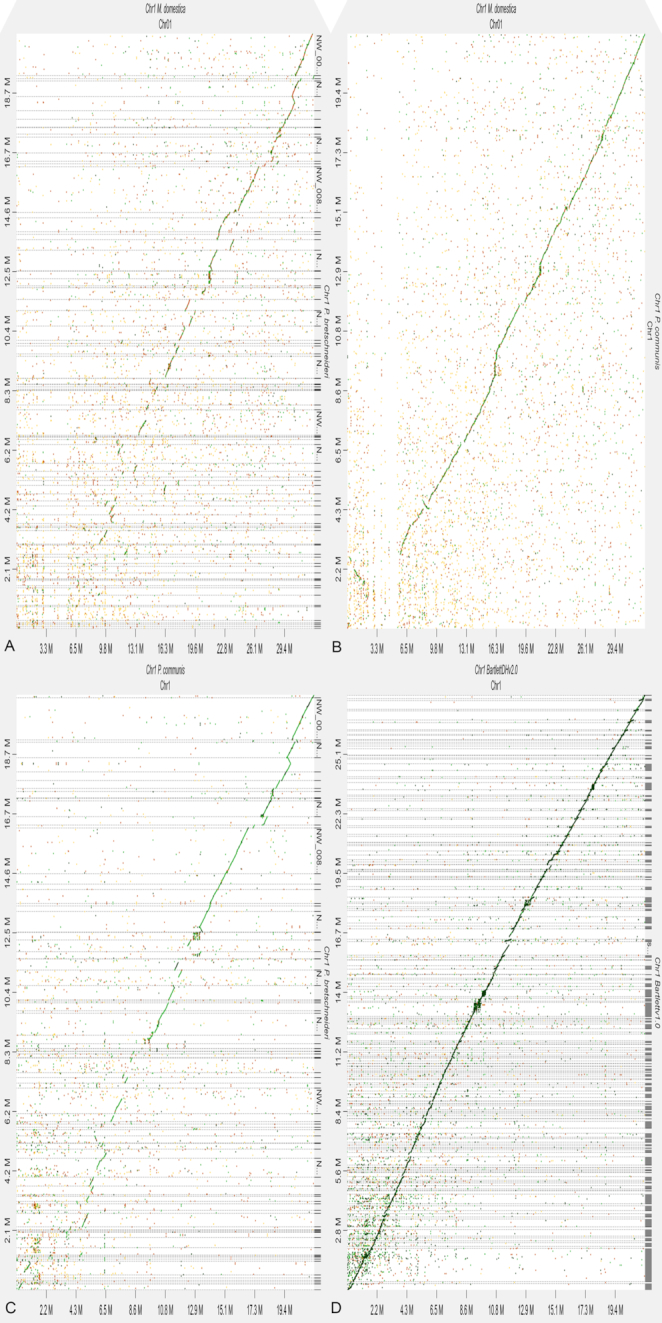
Chromosome 1 alignment dot plots. Dot plots are produced using the DGENIE software [30] and alignments with minimap2 (v2.16). (a) Dot plot of Chromosome 1 *P*.×*bretschneideri* to *P. communis—*BartlettDHv2.0 (top left). (b) Dot plot of Chromosome 1 *P. communis* BartlettDHv2.0 to *M*.×*domestica*—GDDH13 (top right). (c) Dot plot of Chromosome 1 *P*.×*bretschneideri* to *M*.×*domestica—*GDDH13 (bottom left). (d) Dot plot of Chromosome 1 *P. communis—*Bartlettv1.0 to *P. communis—*BartlettDHv2.0 (bottom right).

All 17 chromosomes of the European pear genome displayed strong nucleotide-level synteny with the recent chromosome-scale assembly of the apple genome [2] (Supplementary Figs S18b–S34b). Although only a scaffold-level assembly of the Asian pear is publicly available at this time, 1,913 of the 2,182 scaffolds (88%) from the Asian pear assembly can be aligned to the European pear assembly. The aligned scaffolds sum to 495 Mb or 99.5% of the Asian pear assembly. Of the 1,913 aligned scaffolds, there are 882 scaffolds totalling 403.8 Mb (or 81% of the Asian pear assembly) that align unambiguously to the 17 assembled pseudomolecules. Numerous small-scale inversions with respect to European pear are evident within Asian pear scaffolds, and any of these small-scale structural differences could prove to be of biological interest. Fig. 5 is included as an example of the inter-species and inter-assembly alignments showing chromosome 1. Alignments for all 17 chromosomes are given in Supplementary Figs S19–S34.

**Figure 6:**
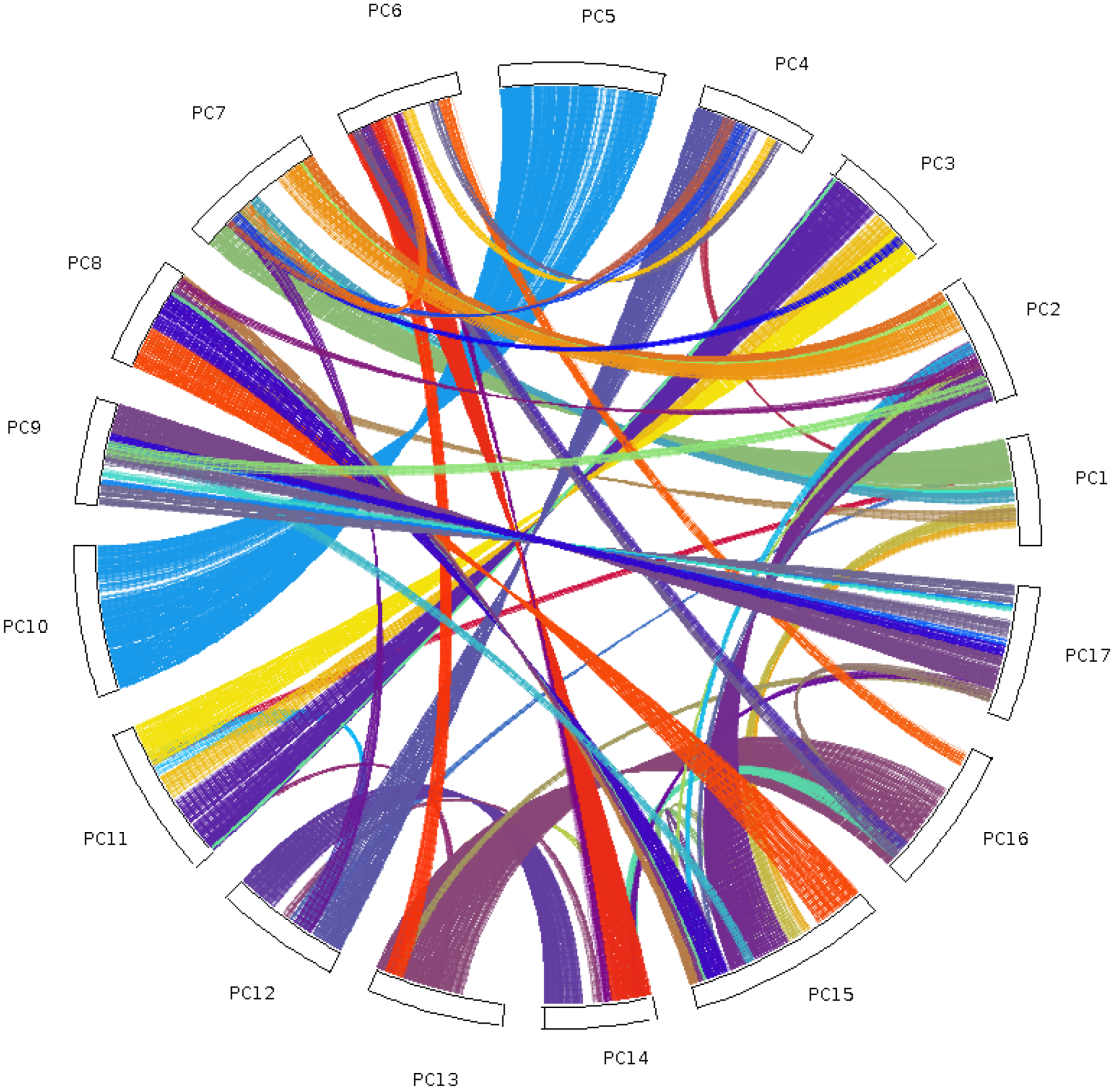
Self-collinearity of *P. communis* (BartlettDHv2). The coloured lines link collinearity blocks representing syntenic regions that were identified by MCScanX.

**Figure 7:**
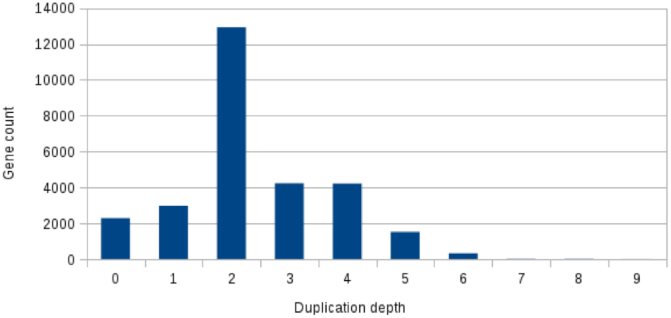
Duplication depth of *F. vesca* gene homologs in *P. communis* (BartlettDHv2). Inter-species collinearity between *F. vesca*[17] and *P. communis* was interrogated using MCScanX and at each gene locus of the *F. vesca* assembly the number of *P. communis*—*F. vesca* inter-species collinear blocks (duplication depth) was counted. The number of *F. vesca* gene homologs having each copy number in the *P. communis* (BartlettDHv2) assembly is then plotted. It can be seen that most gene loci from *F. vesca* occur twice in *P. communis*.

Self-synteny of the genome based on collinear gene blocks reveals that the syntenic chromosome pairs for apple [7] and pear [10] (LG3 and LG11, LG5 and LG10, LG9 and LG17, and LG13 and LG16) are clearly identifiable (Fig. 6) and most collinear regions in strawberry correspond to 2 regions in European pear (Fig. 7), as described for both apple and Asian pear [9, 10]. Hence, the BartlettDHv2.0 assembly confirms that macrosyntenic chromosome structure is conserved across the Malinae.

### Revision of the gene number relative to *P. communis* version 1 assembly

Many scaffolds from the Bartlettv1.0 assembly align to overlapping positions on the BartlettDHv2.0 assembly. These overlapping scaffolds most likely represent assembly of both haplotypes at the same genomic locus. Over-assembly is a danger when assembling a highly heterozygous genome and such separation of the haplotypes led to over-estimation of the gene number for apple [2, 7]. Re-examination of apple gene predictions and removal of overlapping gene models enabled Wu et al. [8] to arrive at a new, lower estimate of the gene number for apple. Gene annotation of the BartlettDHv2.0 assembly resulted in a lower number of predicted genes than reported for [8] the Bartlettv1.0 assembly [9]. When Bartlettv1.0 gene models were aligned to the BartlettDHv2.0 assembly and overlapping gene models were collapsed down to a single locus, only 37,997 independent gene loci were identified. Thus, the removal of overlapping genes brings the number of gene predictions for the 2 *P. communis* assembly versions much more closely in line.

### Conclusions

Cost-effective, high-throughput, long-read sequencing is democratizing the effective assembly of complex genomes, particularly repeat-rich plant genomes. These advances in sequencing technology have enabled the improvement or complete re-assembly of the draft genome sequences that have been typical of non-model organisms, including those of *Pyrus* species. This new improved assembly of the genome of *P. communis* will enable step changes in the progression of genome-based technologies for pear breeders, analogous to those being developed for *Malus* following publication of the Golden Delicious v3.0 assembly [26]. These include the ability to undertake genomic selection, and develop genetic markers based on candidate genes for traits of interest to breeders. These markers could be identified in the genome assembly following quantitative trait locus mapping, or GWAS. Such technologies will enable more efficient and targeted breeding of new varieties of pear with attributes that are desired by consumers and are also grower-friendly.

## Materials and Methods

### Breeding the doubled haploid plant from “Bartlett”

In 1994, the European pear variety “Bartlett” (synonymous “Williams”) was crossed as a female parent with the variety “Passe Crassane” (male). Among the 971 seedlings obtained after sowing in the greenhouse in 1996, 1 showed the typical phenotype of pear haploid plants, i.e., a smaller size compared to diploid seedlings, with a slender stem and narrow, thin leaves of a pale green colour [27]. This haploid plant (referenced W65) was confirmed by flow cytometry and propagated *in vitro* until development was sufficient for a chromosome doubling experiment, which was performed in 1998 with oryzalin on the basis of a protocol adapted from apple [28]. The doubled haploid plant W65DH (here called “Bartlett.DH”) was confirmed as homozygous by isozyme and microsatellite markers [27] and also with the recently developed 70K SNP array [29] (data not shown). “Bartlett.DH” was grafted on rootstock “Adams” and is kept in an experimental orchard at INRA, Angers, France.

### Sample preparation and sequencing

For Illumina sequencing, genomic DNA from Bartlett.DH was purified from young rolled leaves and young meristem tissue using the NucleoSpin Plant II DNA extraction kit (Macherey-Nagel GmbH, Düren, Germany), following the manufacturer's instructions. One Illumina PE library was constructed at CNAG-CRG, Barcelona, Spain, with 340-bp insert size according to KAPA Library Preparation Kit with no PCR Library Amplification/Illumina series (Roche-Kapa Biosystems) protocol and sequenced on HiSeq2000 (v4) in a single lane. Illumina adaptor sequences were clipped using the Scythe software. Illumina sequences were trimmed at their 3′ end where the average quality within a 25-bp window fell below Q20 using the Sickle software. For the BioNano and PacBio single-molecule real-time (SMRT) sequencing, genomic DNA was extracted using a modified nuclei preparation method [31] identical to that used by Daccord et al. [2] followed by an additional phenol-chloroform purification step. Thirty SMRT cells were sequenced on the Pacific Biosciences RSII platform with the P5-C3 chemistry at the Genome Center at UC Davis.

### Hi-C library preparation and sequencing

The *in situ* Hi-C library preparation was performed according to a protocol established for rice seedlings with minor modifications [32]. The libraries were made from 2 biological replicates of “Bartlett.DH”; for each replicate, 0.5 g of fixed leaves were used as the starting material. Due to the presence of a large amount of cellular debris after isolation of nuclei, the nuclear pellet was divided into 5 parts prior to chromatin digestion with DpnII. The Hi-C libraries were sent to the Australian Genome Research Facility (Melbourne, Australia) for sequencing using 1 lane of 100-bp PE sequencing using a HiSeq2000 (Illumina Inc.).

### BioNano Genomics genome mapping

Agarose plug embedded nuclei were Proteinase K treated for 2 days followed by RNAse treatment (Biorad CHEF Genomic DNA Plug Kit). DNA was recovered from agarose plugs according to IrysPrep™ Plug Lysis Long DNA Isolation guidelines (BioNano Genomics). Of the isolated DNA, 300 ng was used for subsequent DNA nicking using Nt.BspQ1 (NEB) incubating for 2 hours at 50°C. Labelling, repair, and staining reactions were performed according to IrysPrep™ Assay NLRS (30024D) protocol. Finally, labelled DNA molecules were analysed on a BioNano Genomics Irys instrument with optimized recipes using 1 Irys chip, 1 flowcell, 9 runs, with 270 cycles in total.

Data were collected and processed using IrisView software V 2.5 together with a XeonPhi (version v4704) accelerated cluster and special software (both BioNano Genomics, Inc.). A *de novo* map assembly was generated using molecules ≥140 kb, and containing a minimum 6 labels per molecule. In total, the molecules used for assembly encompassed 291 Mb equivalent space and on average 8 labels per 100-kb molecule size. For the assembly process, stringency settings for “alignment” and “refineAlignment” were set to 1e−8 and 1e−9, respectively. The assembly was performed by applying 4 iterations, where each iteration consisted of an extension and merging step.

Hybrid scaffolding was done using “hybrid scaffolding_config_aggressive” settings of IrysView.

### Genome assembly and scaffolding

The genome assembly workflow began with *de novo* assembly of contigs from the PacBio long reads using 2 tools, Canu (version 1.5) and Falcon (version 0.5). For each assembler the most important assembly parameters were systematically varied (Supplementary Methods), as defined by the tool developers, and by consideration of assembly theory (e.g., overlap length, overlap identity for overlap layout consensus assembly). Optimal settings were selected by comparison of assembly statistics (total size assembled and contig N50) and by alignment of Illumina PE data to the assembly with bowtie2 [27] (using the “very fast” preset). For all PacBio assemblies the consensus step was performed by running Quiver (Genomic Consensus version 2.3.3) [28] (with default parameters) on raw PacBio contigs and using the full 63× of PacBio data.

Assembled contigs were further joined into scaffolds using a combination of BioNano optical mapping data, Hi-C chromatin conformation capture data, and genetic maps. The best assemblies from Canu and Falcon were independently combined with BioNano optical mapping data using the IrysView software to develop the Canu + BioNano (CB) and Falcon + BioNano (FB) assemblies, respectively. The BioNano scaffolding process identified conflicts between the assembled contigs and the optical map, indicating some degree of misassembly in both Canu and Falcon results.

### Assembly polishing

Pilon (version1.21) [31] was run iteratively on the assembly, with Illumina sequence realigned to the polished assembly at each iteration and then alignments passed to Pilon to call the next consensus. Alignments for Pilon were produced using BWA mem (v0.7.17) with default settings. *k*-mer spectrum comparisons were made using the *k*-mer analysis toolkit (KAT) (version 2.3.4) [13] (KAT comp) at a *k*-mer size of 32, and the metric used to assess each iteration was the number of *k*-mers shared between the assembly and the Illumina reads. In a second consensus phase, RNA-Seq reads were aligned as single end (SE), to the genome using Hisat (version 2.1.0) [33] with default parameters. This time the effectiveness of consensus calling was assessed by analysis of full-length alignments of assembled RNA-Seq transcripts. All transcripts designated as “complete” by Evigene [29] were aligned to the genome with BLAT [13] (version 3.4, with [34] minimum match identity 90%). Alignments were filtered to retain only full-length alignments (i.e., from query start to query end). Finally, the number of gaps in the alignments (query gaps + target gaps) was used as a metric with the rationale that this serves as a proxy for the number of indels in alignments of assembled messenger RNA sequence.

### Scaffold validation using a high-density genetic map

A high-density genetic map was developed using a 100-individual “Old Home” × “Bartlett” F_1_ population and the Axiom™ Pear 70K Genotyping Array [35]. Markers were filtered to have <5% missing data and fit segregation ratios of 1:1 and 1:2:1 (α = 0.01). Mapping was conducted in an iterative process using the maximum likelihood algorithm in JoinMap 5 [36]. After each round of mapping, a graphical genotyping approach was used to identify and fix marker order errors and regions with low marker density caused by segregation distortion. Markers that fitted segregation ratios of 2:1 and 2:3:1 (α = 0.01) were added to the dataset after a high-quality framework map was constructed to improve the low-density regions.

The Bartlett parental map produced by JoinMap included 11,474 markers. This map was used to validate and anchor the scaffolds from both the CB and FB assemblies. SNP probe sequences from the array [35] used in the construction of the genetic map were mapped to the assembly with BLAT (version 3.4) [34]. Alignments were filtered to retain only markers perfectly matched to unique loci in the assembly as well as those with a maximum of 2 mismatches in the second best hit. The resulting alignments were queried to identify problematic scaffolds mapped with SNP probes from different LGs. The number of scaffolds with SNP probes mapped from different LGs was used as a metric in the quality assessment of the FB and CB assemblies. After selection of the CB assembly, its scaffolds were broken at the 3 positions where SNP mapping switched from 1 LG to another. Each scaffold breaking was performed by dividing the scaffold at the position 500 bp past the last good SNP marker.

### Scaffold clustering and genome anchoring using Hi-C

Hi-C reads were aligned to the polished scaffolds in CB with Bowtie2 (version 2.3.3.1) [37]. Each of the paired reads was aligned independently; then these SE alignments were subsequently merged as recommended by the LACHESIS developers. Based on the alignments, CB scaffolds were arranged into 17 ordered and oriented clusters using the LACHESIS software [38]. As an internal check, the process was completed on 2 different random 95% sub-samplings of the Hi-C data, as well as on the full data set. The clusters produced by all 3 of these LACHESIS runs were identical. LACHESIS produces groups of scaffolds that are ordered and oriented relative to each other. These scaffold groupings were compared with the genetic map and the consistency of these sources of information was assessed. The SNP probe mapping at the scaffold validation step was compared with the clusters produced by LACHESIS.

### Illumina assembly

The Illumina data were also assembled on their own, using the de Bruijn graph–based assembler SOAPdenovo2 (version 2.04) [39]. This assembly was used in various ways during the course of the pear genome project (for further scaffold validation, for training the *ab initio* gene predictors, etc.). The Illumina data were assembled twice. The first pass contigs were screened using the Kraken [40] software and an index built from the entire RefSeq database. Reads aligning to contaminant contigs were removed and the remaining data were assembled again.

### Repeat annotation

Repbase (v16.02) [41] was used to identify repeats by using RepeatMasker (version 4.0.5) [42]. RepeatModeler (version 1.0.8) [42] was used to build *de novo* repeats. HODOR sequences [2] were identified by blasting the apple HODOR sequence onto the assembly.

### Transcriptome assembly

The 26.6-Gb “Bartlett” RNA-Seq data (SRA accession numbers SRR1572981 to SRR1572991) were assembled *de novo*, using Trinity (version 2.2.0) [43] and also genome guided, using both Cufflinks (version 2.2.1) [44] and Trinity-GG (version 2.2.0) [45]. All transcripts from these 3 assemblies were pooled and input into the EviGene pipeline [29], which produces a non-redundant transcript database classified into putative primary and alternative transcripts.

### Gene annotation

Gene prediction was guided by the non-redundant transcriptome assembly, as well as by spliced alignments from 3 sources: CDS from closely related species (apple and Asian pear), proteins from these and other less related plant species (*Arabidopsis*, rice, tomato), and RNA-Seq read data aligned onto the genome. All assembled European pear transcripts classified as both full length and primary by EviGene were input to the ORF finder Transdecoder (version 3.0.0) [46] to give a set of predicted CDS sequences. These predicted CDS and CDS from closely related species were aligned to the genome using BLAT (version 3.4) [34] and Genome Threader (version 1.7.0) [22]. Protein alignments were performed using Genome Threader. Mapping of all these evidence sources was first made to Illumina contigs and a training set for the training of *ab initio* gene predictors was constructed by manual annotation of genes on these contigs. Both Augustus (version 3.3) [47] and EuGène (version 4.2) [48] gene predictors were trained using this manually annotated training set.

Spliced alignments of RNA-Seq reads to the genome provide strong evidence for the structure of genes by delineating intron-exon boundaries. RNA-Seq data downloaded from NCBI/SRA were aligned to the pear genome using HiSat (version 2.1.0) [33] with custom parameters. This evidence was leveraged by providing Augustus (version 3.3) [47] with “hints” files detailing the intron-exon boundaries and providing EuGène (version 4.2) [48] with splice site models generated by the SpliceMachine software (version 1.2) [49]. Spliced alignments of assembled transcripts were leveraged by passing them to the PASA pipeline (version 2.3.1) [43, 50], which constructs a genome-based transcriptome assembly. PASA-assembled transcripts were then processed by Transdecoder (version 5.0.2) to produce a set of open reading frames (ORFs) as genome-based GFF coordinates.

*Ab initio* gene predictions were performed with Augustus and EuGène using models trained on the manually annotated Illumina sequence. Augustus was executed with hint files conveying information about the spliced mappings of RNA-Seq reads, assembled transcripts, CDS sequences and proteins, and the repeat annotation of the genome. Similarly, these supporting hints were supplied to EuGène and the prediction was run on repeat masked sequence (with soft masking). The *ab initio* gene models from Augustus and EuGène were combined with the PASA gene models as well as the gene models produced by Genome Threader alignment of proteins, CDS, and assembled transcripts. The EVidenceModeler software (version 1.1.1) [51] was used to combine these different gene models and evidence sources. Finally, the EVidenceModeler annotation was taken and used to retrain EuGène. A final EuGène iteration using this EVidenceModeler annotation as an evidence track helped to clean up the splice boundaries of some CDSs.

### *K*_S_-based paralog and ortholog age distributions

Paralog age distributions of synonymous substitutions per synonymous site (*K*_S_) were constructed as previously described [52], except using PhyML [53] instead of average linkage hierarchical clustering for tree construction. Briefly, to build the paranome, an all-against-all BLASTP search was performed with an E-value cutoff of 1 × 10^−10^, followed by gene family construction using the mclblastline pipeline (v10–201) [54, 55]. Gene families with >400 members were removed. Each gene family was aligned using MUSCLE (v3.8.31) [56], and *K*_S_ estimates for all pairwise comparisons within a gene family were obtained through maximum likelihood (ML) estimation using the CODEML program [57] of the PAML package (v4.4c) [58]. Gene families were then subdivided into subfamilies for which *K*_S_ estimates between members did not exceed a value of 5. To correct for the redundancy of *K*_S_ values [a gene family of *n* members produces *n*(*n* – 1)/2 pairwise *K*_S_ estimates for *n* – 1 retained duplication events], a phylogenetic tree was constructed for each subfamily using PhyML [53] under default settings. For each duplication node in the resulting phylogenetic tree, all *m K*_S_ estimates between the 2 child clades were added to the *K*_S_ distribution with a weight of 1/*m* (where *m* is the number of *K*_S_ estimates for a duplication event), so that the weights of all *K*_S_ estimates for a single duplication event summed to 1.

*K*_S_-based ortholog age distributions were constructed by identifying 1-to-1 orthologs between species using InParanoid [59] with default settings, followed by *K*_S_ estimation using the CODEML program as above. CDSs for *M*. × *domestica* and *P*. × *bretschneideri* were obtained from the apple GDDH13 genome project [60] and from the PLAZA dicot database [61, 62].

### Gene family analysis

Proteins of *P*.×*bretschneideri* [8], *M*.×*domestica* [2], *F. vesca* [17], *P.persica* [18], *R. chinensis* [19], *R. occidentalis* [1], *V. vinifera* [20], and *A. thaliana* [21] were collected for all-against-all alignment to predicted proteins for *P. communis* with BLASTP [58] (E-value < 10^−4^). These alignments were passed to the OrthoFinder [63] software, which was run with default parameters.

### Collinearity and synteny

All-against-all protein alignments were also passed to the MCScanX software [64] to identify collinearity blocks. Self-collinearity of pear was plotted using the circle_plotter program bundled with MCScanX, after rebuilding the collinearity blocks with a minimum block size of 20 to reduce the noise level. Duplication depth of strawberry homologs in pear was counted with the dissect_multiple_alignment script bundled with MCScanX. DNA level synteny between *P. communis, P*. × *bretschneideri, M*.×*domestica*, and the 2 assembly versions for *P. communis* were all plotted using DGenies [30] with default parameters after aligning with minimap2 (version 2.16).

## Availability of Supporting Data and Materials

Genome assembly and gene predictions have been submitted to the Genome Database for Rosaceae [65] and are freely available [66] alongside tools like JBrowse and BLAST. The community annotation portal for *P. communis* is available for read-only access [67]. To participate in the ongoing manual annotation efforts please contact Yves Van de Peer.

Supporting data, including the genome assembly, annotations, and raw sequencing information, are also available via the *GigaScience* database, GigaDB [68].

## Additional Files

**Figure S1:** Haplotype map for the BartlettDHv2.0. The blue and red colours represent the 2 different haplotypes of the diploid Bartlett progenitor.

**Figure S2:** Marey plot of Chr1 with heat map of dispersed repeats and genes in bins of 200 kb. The lighter the colour the more elements are present. Genetic positions refer to the high-density map of Bartlett. Dots represent the genetic and physical position (on BartlettDHv2.0) of 11,474 SNPs.

**Figure S3:** Marey plot of Chr2 with heat map of dispersed repeats and genes in bins of 200 kb. The lighter the colour the more elements are present. Genetic positions refer to the high-density map of Bartlett. Dots represent the genetic and physical position (on BartlettDHv2.0) of 11,474 SNPs.

**Figure S4:** Marey plot of Chr3 with heat map of dispersed repeats and genes in bins of 200 kb. The lighter the colour the more elements are present. Genetic positions refer to the high-density map of Bartlett. Dots represent the genetic and physical position (on BartlettDHv2.0) of 11,474 SNPs.

**Figure S5:** Marey plot of Chr4 with heat map of dispersed repeats and genes in bins of 200 kb. The lighter the colour the more elements are present. Genetic positions refer to the high-density map of Bartlett. Dots represent the genetic and physical position (on BartlettDHv2.0) of 11,474 SNPs.

**Figure S6:** Marey plot of Chr5 with heat map of dispersed repeats and genes in bins of 200 kb. The lighter the colour the more elements are present. Genetic positions refer to the high-density map of Bartlett. Dots represent the genetic and physical position (on BartlettDHv2.0) of 11,474 SNPs.

**Figure S7:** Marey plot of Chr6 with heat map of dispersed repeats and genes in bins of 200 kb. The lighter the colour the more elements are present. Genetic positions refer to the high-density map of Bartlett. Dots represent the genetic and physical position (on BartlettDHv2.0) of 11,474 SNPs.

**Figure S8:** Marey plot of Chr7 with heat map of dispersed repeats and genes in bins of 200 kb. The lighter the colour the more elements are present. Genetic positions refer to the high-density map of Bartlett. Dots represent the genetic and physical position (on BartlettDHv2.0) of 11,474 SNPs.

**Figure S9:** Marey plot of Chr8 with heat map of dispersed repeats and genes in bins of 200 kb. The lighter the colour the more elements are present. Genetic positions refer to the high-density map of Bartlett. Dots represent the genetic and physical position (on BartlettDHv2.0) of 11,474 SNPs.

**Figure S10:** Marey plot of Chr9 with heat map of dispersed repeats and genes in bins of 200 kb. The lighter the colour the more elements are present. Genetic positions refer to the high-density map of Bartlett. Dots represent the genetic and physical position (on BartlettDHv2.0) of 11,474 SNPs.

**Figure S11:** Marey plot of Chr10 with heat map of dispersed repeats and genes in bins of 200 kb. The lighter the colour the more elements are present. Genetic positions refer to the high-density map of Bartlett. Dots represent the genetic and physical position (on BartlettDHv2.0) of 11,474 SNPs.

**Figure S12:** Marey plot of Chr11 with heat map of dispersed repeats and genes in bins of 200 kb. The lighter the colour the more elements are present. Genetic positions refer to the high-density map of Bartlett. Dots represent the genetic and physical position (on BartlettDHv2.0) of 11,474 SNPs.

**Figure S13:** Marey plot of Chr12 with heat map of dispersed repeats and genes in bins of 200 kb. The lighter the colour the more elements are present. Genetic positions refer to the high-density map of Bartlett. Dots represent the genetic and physical position (on BartlettDHv2.0) of 11,474 SNPs.

**Figure S14:** Marey plot of Chr13 with heat map of dispersed repeats and genes in bins of 200 kb. The lighter the colour the more elements are present. Genetic positions refer to the high-density map of Bartlett. Dots represent the genetic and physical position (on BartlettDHv2.0) of 11,474 SNPs.

**Figure S15:** Marey plot of Chr14 with heat map of dispersed repeats and genes in bins of 200 kb. The lighter the colour the more elements are present. Genetic positions refer to the high-density map of Bartlett. Dots represent the genetic and physical position (on BartlettDHv2.0) of 11,474 SNPs.

**Figure S16:** Marey plot of Chr15 with heat map of dispersed repeats and genes in bins of 200 kb. The lighter the colour the more elements are present. Genetic positions refer to the high-density map of Bartlett. Dots represent the genetic and physical position (on BartlettDHv2.0) of 11,474 SNPs.

**Figure S17:** Marey plot of Chr16 with heat map of dispersed repeats and genes in bins of 200 kb. The lighter the colour the more elements are present. Genetic positions refer to the high-density map of Bartlett. Dots represent the genetic and physical position (on BartlettDHv2.0) of 11,474 SNPs.

**Figure S18:** Marey plot of Chr17 with heat map of dispersed repeats and genes in bins of 200 kb. The lighter the colour the more elements are present. Genetic positions refer to the high-density map of Bartlett. Dots represent the genetic and physical position (on BartlettDHv2.0) of 11,474 SNPs. This Marey plot only shows data for the first 12 Mb of Chr17. Markers in the remaining region show a high level of segregation distortion (and therefore were not mapped genetically) that should be further investigated.

**Figure S19:** Chromosome 1 alignment dot plots. Dot plots are produced using the DGENIE software [1] and alignments with minimap2 (v2.16).

**Figure S20:** Chromosome 2 alignment dot plots. Dot plots are produced using the DGENIE software [1] and alignments with minimap2 (v2.16).

**Figure S21:** Chromosome 3 alignment dot plots. Dot plots are produced using the DGENIE software [1] and alignments with minimap2 (v2.16).

**Figure S22:** Chromosome 4 alignment dot plots. Dot plots are produced using the DGENIE software [1] and alignments with minimap2 (v2.16).

**Figure S23:** Chromosome 5 alignment dot plots. Dot plots are produced using the DGENIE software [1] and alignments with minimap2 (v2.16).

**Figure S24:** Chromosome 6 alignment dot plots. Dot plots are produced using the DGENIE software [1] and alignments with minimap2 (v2.16).

**Figure S25:** Chromosome 7 alignment dot plots. Dot plots are produced using the DGENIE software [1] and alignments with minimap2 (v2.16).

**Figure S26:** Chromosome 8 alignment dot plots. Dot plots are produced using the DGENIE software [1] and alignments with minimap2 (v2.16).

**Figure S27:** Chromosome 9 alignment dot plots. Dot plots are produced using the DGENIE software [1] and alignments with minimap2 (v2.16).

**Figure S28:** Chromosome 10 alignment alignment dot plots. Dot plots are produced using the DGENIE software [1] and alignments with minimap2 (v2.16).

**Figure S29:** Chromosome 11 alignment alignment dot plots. Dot plots are produced using the DGENIE software [1] and alignments with minimap2 (v2.16).

**Figure S30:** Chromosome 12 alignment alignment dot plots. Dot plots are produced using the DGENIE software [1] and alignments with minimap2 (v2.16).

**Figure S31:** Chromosome 13 alignment alignment dot plots. Dot plots are produced using the DGENIE software [1] and alignments with minimap2 (v2.16).

**Figure S32:** Chromosome 14 alignment alignment dot plots. Dot plots are produced using the DGENIE software [1] and alignments with minimap2 (v2.16).

**Figure S33:** Chromosome 15 alignment alignment dot plots. Dot plots are produced using the DGENIE software [1] and alignments with minimap2 (v2.16).

**Figure S34:** Chromosome 16 alignment alignment dot plots. Dot plots are produced using the DGENIE software [1] and alignments with minimap2 (v2.16).

**Figure S35:** Chromosome 17 alignment alignment dot plots. Dot plots are produced using the DGENIE software [1] and alignments with minimap2 (v2.16).

**Figure S36:** *k*-mer spectrum copy number plot for BartlettDHv2 (*k* = 17)

**Figure S37:** *k*-mer spectrum copy number plot for *P. × bretschneideri*(*k* = 17)

**Table S1:** GO terms enriched in *P. communis* genes determined to be pomme specific

**Table S2:** Full parameter settings for all tools.

**Table S3**: Assembly parameter settings including non-optimal assemblies

giz138_GIGA-D-19-00257_Original_Submission

giz138_GIGA-D-19-00257_Revision_1

giz138_GIGA-D-19-00257_Revision_2

giz138_Response_to_Reviewer_Comments_Original_Submission

giz138_Response_to_Reviewer_Comments_Revision_1

giz138_Reviewer_1_Report_Original_Submission C Robin Buell -- 7/31/2019 Reviewed

giz138_Reviewer_2_Report_Original_Submission Anna Syme -- 8/7/2019 Reviewed

giz138_Supplemental_Figures_and_Tables

## Abbreviations

BAC: bacterial artificial chromosome; BLAST: Basic Local Alignment Search Tool; bp: base pairs; BUSCO: Benchmarking Universal Single-Copy Orthologs; BWA: Burrows-Wheeler Aligner; CB: Canu + BioNano; CDS: coding sequence; DH: double-haploid; FB: Falcon + BioNano; Gb: gigabase pairs; GO: Gene Ontology; GWAS: genome-wide association studies; HODOR: high-copy golden delicious repeat; kb: kilobase pairs; LG: linkage group; LTR: long terminal repeat; Mb: megabase pairs; NCBI: National Center for Biotechnology Information; nt: nucleotides; ORCAE: Online Resource for Community Annotation of Eukaryotes; ORF: open reading frame; PE: paired-end; RNA-Seq: RNA sequencing; SMRT: single-molecule real-time; SNP: single-nucleotide polymorphism; SRA: Sequence Read Archive; WGD: whole-genome duplication; WGS: whole-genome sequencing.

## Competing Interests

The authors declare that they have no competing interests.

## Funding

We thank the California Pear Advisory Board and the Pear Pest Management Research Fund for providing funding to perform PacBio sequencing of the DH and genotyping of the mapping population and the Provincia Autonoma di Trento that partially funded this project. Y.V.d.P. acknowledges support from the European Union Seventh Framework Programme (FP7/2007-2013) under European Research Council Advanced Grant Agreement 322739–DOUBLEUP.

## Authors' Contributions

G.L. and L.B. assembled the genome and performed assembly quality assurance. L.V.B. and E.S. performed Bionano optical mapping. G.L. and S.R. performed gene annotation and functional annotation. G.L. performed *k*-mer analysis, repeat annotation, orthology analysis, synteny analysis, inter assembly comparisons, transcriptome assembly, Hi-C data analysis, interpreted the results and wrote the manuscript. C.L. prepared Hi-C libraries. C.D. contributed to the BUSCO analysis, to interspecies synteny analysis, performed contamination screening of Illumina assemblies and produced the orthology plot. R.L. performed analysis of the WGD. Under the scientific authority of Y.L., P.G. identified, checked, doubled, multiplied, and has maintained W65/Bartlett.DH since 1996, with DNA finally extracted by J.M.C. S.M., M.T., L.B., J.D.Z., N.V.B, D.C., and C.D. contributed linkage maps. D.B.N., M.T., D.C., C.H.D., S.M., and R.V. contributed funding toward the sequencing. G.L., S.R., L.B., M.T., D.C., D.B.N., C.H.D., Y.V.D.P., and R.V. designed the project. D.C., L.B., M.T., C.D., S.M., J.D.Z., N.V.B, R.L., S.R., C.L., E.S., A.C., S.E.G., and Y.V.D.P. contributed to writing the manuscript. All authors approved the final manuscript.

## References

[1] VanBuren R, Bryant D, Bushakra JM, et al. The genome of black raspberry (*Rubus occidentalis*). Plant J. 2016;87(6):535–47., doi:10.1111/tpj.13215. 27228578

[2] Daccord N, Celton J-M, Linsmith G, et al. High-quality de novo assembly of the apple genome and methylome dynamics of early fruit development. Nat Genet. 2017;49(7):1099–106., doi:10.1038/ng.3886. 28581499

[3] Van de Peer Y Size does matter. Nat Plants. 2018;4(11):859–60., doi:10.1038/s41477-018-0293-8. 30390078

[4] Micheletti D, Troggio M, Zharkikh A, et al. Genetic diversity of the genus *Malus* and implications for linkage mapping with SNPs. Tree Genet Genomes. 2011;7(4):857–68., doi:10.1007/s11295-011-0380-8.

[5] Xu X, Pan S, Cheng S, et al. Genome sequence and analysis of the tuber crop potato. Nature. 2011;475(7355):189–95., doi:10.1038/nature10158. 21743474

[6] Haberer G, Young S, Bharti AK, et al. Structure and architecture of the maize genome. Plant Physiol. 2005;139(4):1612–24., doi:10.1104/pp.105.068718. 16339807 PMC1310546

[7] Velasco R, Zharkikh A, Affourtit J, et al. The genome of the domesticated apple (*Malus* × *domestica* Borkh.). Nat Genet. 2010;42(10):833–9., doi:10.1038/ng.654. 20802477

[8] Wu J, Wang Z, Shi Z, et al. The genome of the pear (*Pyrus bretschneideri*Rehd.). Genome Res. 2013;23(2):396–408., doi:10.1101/gr.144311.112. 23149293 PMC3561880

[9] Chagné D, Crowhurst RN, Pindo M, et al. The draft genome sequence of European pear (*Pyrus communis* L. ‘Bartlett’). PLoS One. 2014;9(4):e92644, doi:10.1371/journal.pone.0092644. 24699266 PMC3974708

[10] Bouvier L, Guérif P, Djulbic M, et al. Chromosome doubling of pear haploid plants and homozygosity assessment using isozyme and microsatellite markers. Euphytica. 2002;123(2):255–62., doi:10.1023/A:1014998019674.

[11] Wu J, Wang Y, Xu J, et al. Diversification and independent domestication of Asian and European pears. Genome Biol. 2018;19(1):77, doi:10.1186/s13059-018-1452-y. 29890997 PMC5996476

[12] Chakravarti A A graphical representation of genetic and physical maps: the Marey map. Genomics. 1991;11(1):219–22. 1765381 10.1016/0888-7543(91)90123-v

[13] Mapleson D, Garcia Accinelli G, Kettleborough G, et al. KAT: a K-mer analysis toolkit to quality control NGS datasets and genome assemblies. Bioinformatics. 2017;33(4):574–6., doi:10.1093/bioinformatics/btw663. 27797770 PMC5408915

[14] Koren S, Walenz BP, Berlin K, et al. Canu: scalable and accurate long-read assembly via adaptive k-mer weighting and repeat separation. Genome Res. 2017;27(5):722–36., doi:10.1101/gr.215087.116. 28298431 PMC5411767

[15] Chin C-S, Peluso P, Sedlazeck FJ, et al. Phased diploid genome assembly with single-molecule real-time sequencing. Nat Methods. 2016;13(12):1050–4., doi:10.1038/nmeth.4035. 27749838 PMC5503144

[16] Watson M, Warr A Errors in long-read assemblies can critically affect protein prediction. Nat Biotechnol. 2019;37(2):124–6., doi:10.1038/s41587-018-0004-z. 30670796

[17] Edger PP, VanBuren R, Colle M, et al. Single-molecule sequencing and optical mapping yields an improved genome of woodland strawberry (*Fragaria vesca*) with chromosome-scale contiguity. Gigascience. 2018;7(2):1–7., doi:10.1093/gigascience/gix124. PMC5801600 29253147

[18] Verde I, Abbott AG, Scalabrin S, et al. The high-quality draft genome of peach (*Prunus persica*) identifies unique patterns of genetic diversity, domestication and genome evolution. Nat Genet. 2013;45(5):487–94., doi:10.1038/ng.2586. 23525075

[19] Raymond O, Gouzy J, Just J, et al. The *Rosa* genome provides new insights into the domestication of modern roses. Nat Genet. 2018;50(6):772–7., doi:10.1038/s41588-018-0110-3. 29713014 PMC5984618

[20] French-Italian Public Consortium for Grapevine Genome Characterization. Jaillon O, Aury J-M, et al. The grapevine genome sequence suggests ancestral hexaploidization in major angiosperm phyla. Nature. 2007;449(7161):463–7., doi:10.1038/nature06148. 17721507

[21] Arabidopsis Genome Initiative. Analysis of the genome sequence of the flowering plant *Arabidopsis thaliana*. Nature. 2000;408(6814):796–815., doi:10.1038/35048692. 11130711

[22] Gremme G, Brendel V, Sparks ME, et al. Engineering a software tool for gene structure prediction in higher organisms. Inf Softw Technol. 2005;47(15):965–78., doi:10.1016/J.INFSOF.2005.09.005.

[23] Pilkington SM, Crowhurst R, Hilario E, et al. A manually annotated *Actinidia chinensis* var. chinensis (kiwifruit) genome highlights the challenges associated with draft genomes and gene prediction in plants. BMC Genomics. 2018;19(1):257, doi:10.1186/s12864-018-4656-3. 29661190 PMC5902842

[24] Sterck L, Billiau K, Abeel T, et al. ORCAE: online resource for community annotation of eukaryotes. Nat Methods. 2012;9(11):1041, doi:10.1038/nmeth.2242. 23132114

[25] Van Bel M, Proost S, Van Neste C, et al. TRAPID: an efficient online tool for the functional and comparative analysis of de novo RNA-Seq transcriptomes. Genome Biol. 2013;14(12):R134, doi:10.1186/gb-2013-14-12-r134. 24330842 PMC4053847

[26] Peace CP, Bianco L, Troggio M, et al. Apple whole genome sequences: recent advances and new prospects. Hortic Res. 2019;6(1):59, doi:10.1038/s41438-019-0141-7. 30962944 PMC6450873

[27] Bouvier L, Zhang Y-X, Lespinasse Y Two methods of haploidization in pear, *Pyrus communis* L.: greenhouse seedling selection and in situ parthenogenesis induced by irradiated pollen. Theor Appl Genet. 1993;87(1-2):229–32., doi:10.1007/BF00223769. 24190217

[28] Bouvier L, Fillon FR, Lespinasse Y Oryzalin as an efficient agent for chromosome doubling of haploid apple shoots in vitro. Plant Breed. 1994;113(4):343–6., doi:10.1111/j.1439-0523.1994.tb00748.x.

[29] EvidentialGene: mRNA Transcript Assembly Software. http://arthropods.eugenes.org/EvidentialGene/trassembly.html. Last accessed: 19.11.2019.

[30] Cabanettes F, Klopp C D-GENIES: dot plot large genomes in an interactive, efficient and simple way. PeerJ. 2018;6:e4958, doi:10.7717/peerj.4958. 29888139 PMC5991294

[31] Jaskiewicz M, Peterhansel C, Conrath U Detection of histone modifications in plant leaves. J Vis Exp. 2011;(55), doi:10.3791/3096. PMC3230183 21969029

[32] Liu C, Cheng Y-J, Wang J-W, et al. Prominent topologically associated domains differentiate global chromatin packing in rice from *Arabidopsis*. Nat Plants. 2017;3(9):742–8., doi:10.1038/s41477-017-0005-9. 28848243

[33] Kim D, Langmead B, Salzberg SL HISAT: a fast spliced aligner with low memory requirements. Nat Methods. 2015;12(4):357–60., doi:10.1038/nmeth.3317. 25751142 PMC4655817

[34] Kent WJ BLAT—The BLAST-Like alignment tool. Genome Res. 2002;12(4):656–64., doi:10.1101/gr.229202. 11932250 PMC187518

[35] Montanari S, Bianco L, Allen BJ, et al. Development of a highly efficient Axiom^TM^ 70 K SNP array for *Pyrus* and evaluation for high-density mapping and germplasm characterization. BMC Genomics. 2019;20:331, doi:10.1186/s12864-019-5712-3. 31046664 PMC6498479

[36] Van Ooijen JW JoinMap® 4.0: software for the calculation of genetic linkage maps in experimental population. Kyazma BV; 2006.

[37] Langmead B, Salzberg SL Fast gapped-read alignment with Bowtie 2. Nat Methods. 2012;9(4):357–9., doi:10.1038/nmeth.1923. 22388286 PMC3322381

[38] Burton JN, Adey A, Patwardhan RP, et al. Chromosome-scale scaffolding of de novo genome assemblies based on chromatin interactions. Nat Biotechnol. 2013;31(12):1119–25., doi:10.1038/nbt.2727. 24185095 PMC4117202

[39] Luo R, Liu B, Xie Y, et al. SOAPdenovo2: an empirically improved memory-efficient short-read de novo assembler. Gigascience. 2012;1(1):18, doi:10.1186/2047-217X-1-18. 23587118 PMC3626529

[40] Wood DE, Salzberg SL Kraken: ultrafast metagenomic sequence classification using exact alignments. Genome Biol. 2014;15(3):R46, doi:10.1186/gb-2014-15-3-r46. 24580807 PMC4053813

[41] Bao W, Kojima KK, Kohany O Repbase Update, a database of repetitive elements in eukaryotic genomes. Mob DNA. 2015;6(1):11, doi:10.1186/s13100-015-0041-9. 26045719 PMC4455052

[42] Smit A, Hubley R, Grenn P RepeatMasker Open-4.0. *RepeatMasker Open-40 5*. http://www.repeatmasker.org. Last accessed: 19.11.2019.

[43] Haas BJ, Papanicolaou A, Yassour M, et al. De novo transcript sequence reconstruction from RNA-seq using the Trinity platform for reference generation and analysis. Nat Protoc. 2013;8(8):1494–512., doi:10.1038/nprot.2013.084. 23845962 PMC3875132

[44] Trapnell C, Roberts A, Goff L, et al. Differential gene and transcript expression analysis of RNA-seq experiments with TopHat and Cufflinks. Nat Protoc. 2012;7(3):562–78., doi:10.1038/nprot.2012.016. 22383036 PMC3334321

[45] Grabherr MG, Haas BJ, Yassour M, et al. Full-length transcriptome assembly from RNA-Seq data without a reference genome. Nat Biotechnol. 2011;29(7):644–52., doi:10.1038/nbt.1883. 21572440 PMC3571712

[46] Transdecoder. https://github.com/TransDecoder. Last accessed: 19.11.2019.

[47] Stanke M, Diekhans M, Baertsch R, et al. Using native and syntenically mapped cDNA alignments to improve de novo gene finding. Bioinformatics. 2008;24(5):637–44., doi:10.1093/bioinformatics/btn013. 18218656

[48] Foissac S, Gouzy J, Rombauts S, et al. Genome annotation in plants and fungi: EuGène as a model platform. Curr Bioinformatics. 2008;3:87–97.

[49] Degroeve S, Saeys Y, De Baets B, et al. SpliceMachine: predicting splice sites from high-dimensional local context representations. Bioinformatics. 2005;21(8):1332–8., doi:10.1093/bioinformatics/bti166. 15564294

[50] Haas BJ, Delcher AL, Mount SM, et al. Improving the *Arabidopsis* genome annotation using maximal transcript alignment assemblies. Nucleic Acids Res. 2003;31(19):5654–66., doi:10.1093/NAR/GKG770. 14500829 PMC206470

[51] Haas BJ, Salzberg SL, Zhu W, et al. Automated eukaryotic gene structure annotation using EVidenceModeler and the Program to Assemble Spliced Alignments. Genome Biol. 2008;9(1):R7, doi:10.1186/gb-2008-9-1-r7. 18190707 PMC2395244

[52] Vanneste K, Van de Peer Y, Maere S Inference of genome duplications from age distributions revisited. Mol Biol Evol. 2013;30(1):177–90., doi:10.1093/molbev/mss214. 22936721

[53] Guindon S, Dufayard J-F, Lefort V, et al. New algorithms and methods to estimate maximum-likelihood phylogenies: assessing the performance of PhyML 3.0. Syst Biol. 2010;59(3):307–21., doi:10.1093/sysbio/syq010. 20525638

[54] MCL - a cluster algorithm for graphs. micans.org/mcl. Last accessed: 19.11.2019.

[55] Enright AJ, Van Dongen S, Ouzounis CA An efficient algorithm for large-scale detection of protein families. Nucleic Acids Res. 2002;30(7):1575–84. 11917018 10.1093/nar/30.7.1575 PMC101833

[56] Edgar RC MUSCLE: multiple sequence alignment with high accuracy and high throughput. Nucleic Acids Res. 2004;32(5):1792–7., doi:10.1093/nar/gkh340. 15034147 PMC390337

[57] Goldman N, Yang Z A codon-based model of nucleotide substitution for protein-coding DNA sequences. Mol Biol Evol. 1994;11(5):725–36., doi:10.1093/oxfordjournals.molbev.a040153. 7968486

[58] Yang Z PAML 4: Phylogenetic analysis by maximum likelihood. Mol Biol Evol. 2007;24(8):1586–91., doi:10.1093/molbev/msm088. 17483113

[59] Ostlund G, Schmitt T, Forslund K, et al. InParanoid 7: new algorithms and tools for eukaryotic orthology analysis. Nucleic Acids Res. 2010;38(Database):D196–203., doi:10.1093/nar/gkp931. 19892828 PMC2808972

[60] Apple genome database. https://iris.angers.inra.fr/gddh13/the-apple-genome-downloads.html. Last accessed: 19.11.2019.

[61] Plaza dicots database. https://bioinformatics.psb.ugent.be/plaza/versions/plaza_v4_dicots/. Last accessed: 19.11.2019.

[62] Van Bel M, Diels T, Vancaester E, et al. PLAZA 4.0: an integrative resource for functional, evolutionary and comparative plant genomics. Nucleic Acids Res. 2018;46(D1):D1190–6., doi:10.1093/nar/gkx1002. 29069403 PMC5753339

[63] Emms DM, Kelly S OrthoFinder: solving fundamental biases in whole genome comparisons dramatically improves orthogroup inference accuracy. Genome Biol. 2015;16(1):157, doi:10.1186/s13059-015-0721-2. 26243257 PMC4531804

[64] Wang Y, Tang H, DeBarry JD, et al. MCScanX: a toolkit for detection and evolutionary analysis of gene synteny and collinearity. Nucleic Acids Res. 2012;40(7):e49, doi:10.1093/nar/gkr1293. 22217600 PMC3326336

[65] Jung S, Lee T, Cheng C-H, et al. 15 years of GDR: new data and functionality in the Genome Database for Rosaceae. Nucleic Acids Res. 2019;47(D1):D1137–45., doi:10.1093/nar/gky1000. 30357347 PMC6324069

[66] Linsmith G, Rombauts S, Montanari S, et al. Entry in the Genome Database for Rosaceae 2019 https://www.rosaceae.org/organism/Pyrus/communis. Last accessed: 19.11.2019.

[67] Linsmith G, Rombauts S, Montanari S, et al. Online resource for Community annotation of Eukaryotes 2019 https://bioinformatics.psb.ugent.be/orcae/. Last accessed: 19.11.2019.

[68] Linsmith G, Rombauts S, Montanari S, et al. Supporting data for “Pseudo-chromosome length genome assembly of a double haploid ‘Bartlett’ pear (*Pyrus communis* L.).”. GigaScience Database. 2019 10.5524/100644. Last accessed: 19.11.2019. PMC6901071 31816089

