# Supplementary material for: Pseudo-chromosome–length genome assembly of a double haploid “Bartlett” pear (Pyrus communis L.)
Source: Gigascience. 2019 Dec 9;8(12):giz138. doi: 10.1093/gigascience/giz138 (PMC6901071; doi:10.1093/gigascience/giz138)
Supplement: giz138_Supplemental_Figures_and_Tables [file giz138_supplemental_figures_and_tables.pdf]

Supplementary Figures

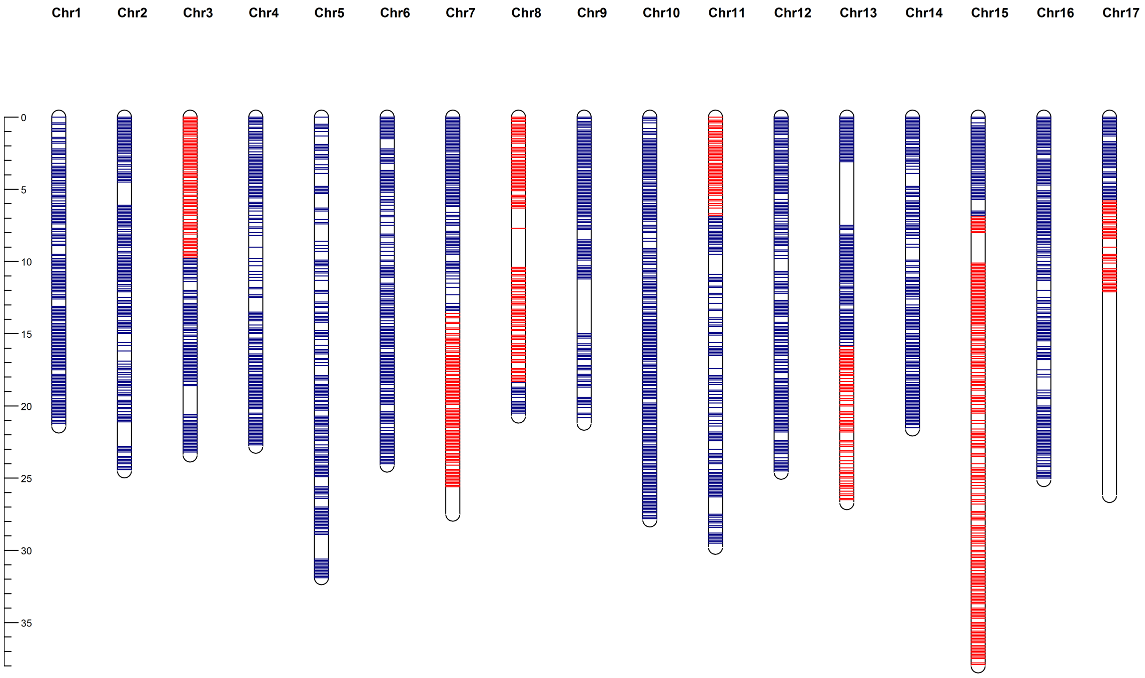

Figure S1: **Haplotype map for the BartlettDHv2.0. The blue and red colours represent the two different haplotypes of the diploid Bartlett progenitor.**

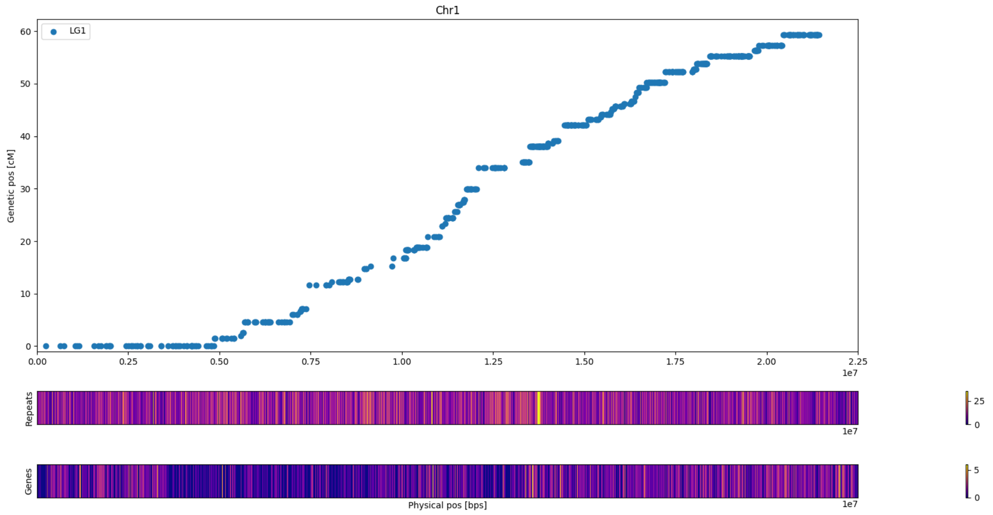

Figure S2: Marey plot of Chr1 with heatmap of Dispersed Repeats and Genes in bins of 200kb. The lighter the colour the more elements are present. Genetic positions refer to the high-density map of Bartlett. Dots represent the genetic and physical position (on of BartlettDHv2.0) of 11,474 SNPs.

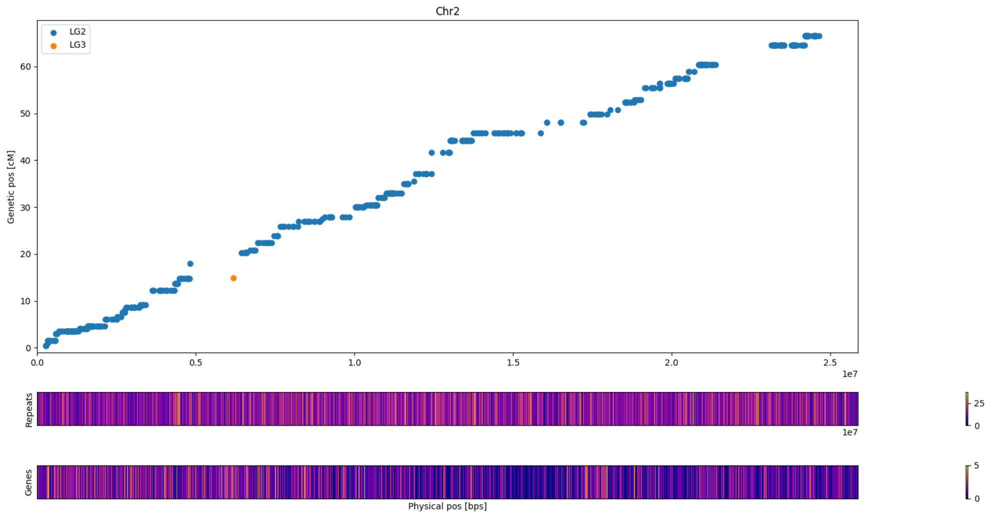

Figure S3: Marey plot of Chr2 with heatmap of Dispersed Repeats and Genes in bins of 200kb. The lighter the colour the more elements are present. Genetic positions refer to the high-density map of Bartlett. Dots represent the genetic and physical position (on of BartlettDHv2.0) of 11,474 SNPs.

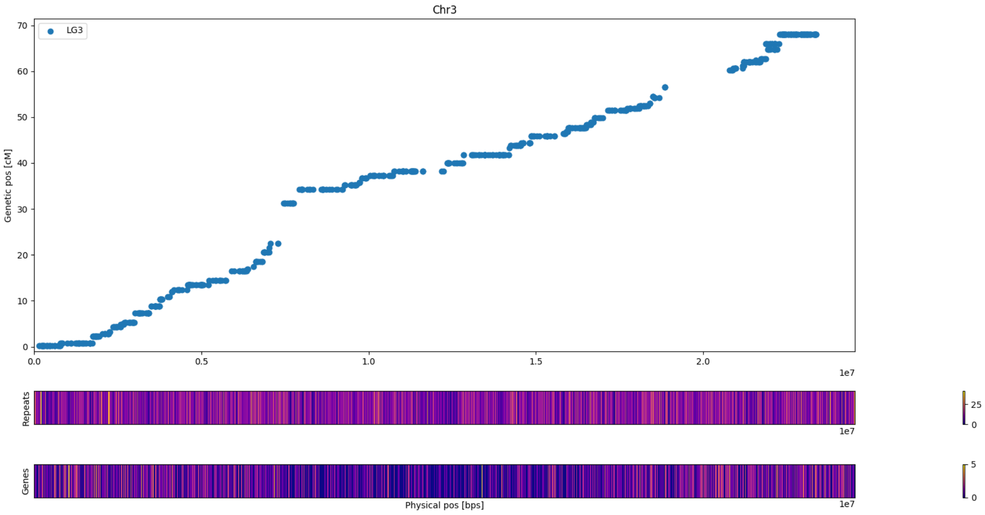

Figure S4: Marey plot of Chr3 with heatmap of Dispersed Repeats and Genes in bins of 200kb. The lighter the colour the more elements are present. Genetic positions refer to the high-density map of Bartlett. Dots represent the genetic and physical position (on of BartlettDHv2.0) of 11,474 SNPs.

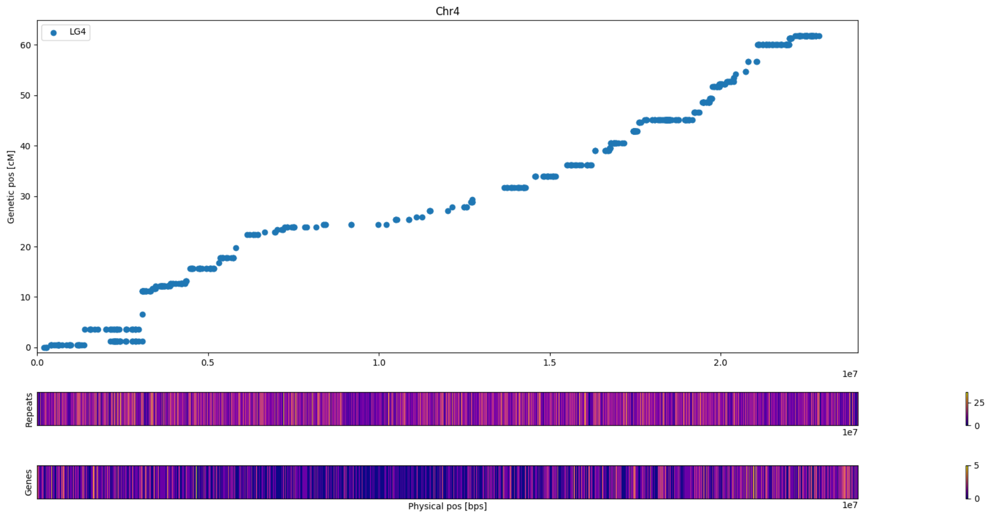

Figure S5: Marey plot of Chr4 with heatmap of Dispersed Repeats and Genes in bins of 200kb. The lighter the colour the more elements are present. Genetic positions refer to the high-density map of Bartlett. Dots represent the genetic and physical position (on of BartlettDHv2.0) of 11,474 SNPs.

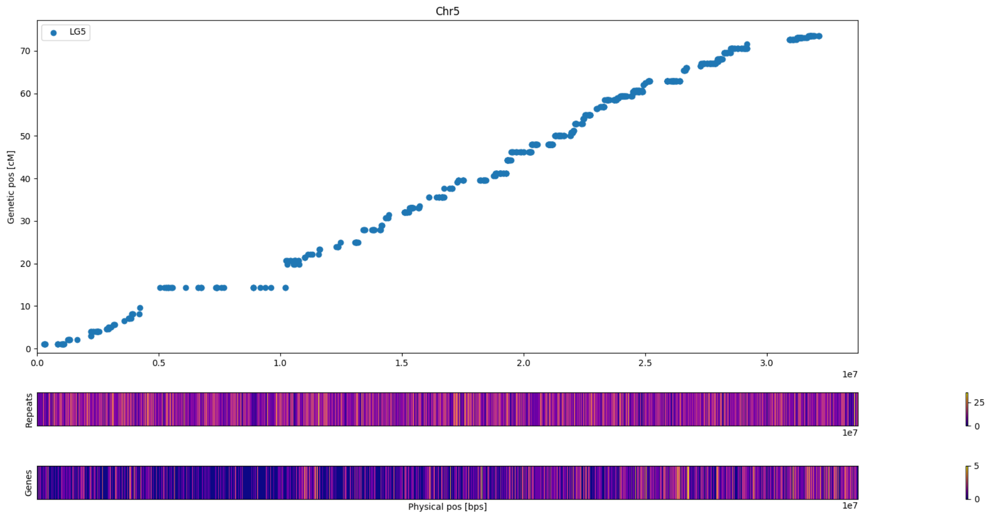

Figure S6: Marey plot of Chr5 with heatmap of Dispersed Repeats and Genes in bins of 200kb. The lighter the colour the more elements are present. Genetic positions refer to the high-density map of Bartlett. Dots represent the genetic and physical position (on of BartlettDHv2.0) of 11,474 SNPs.

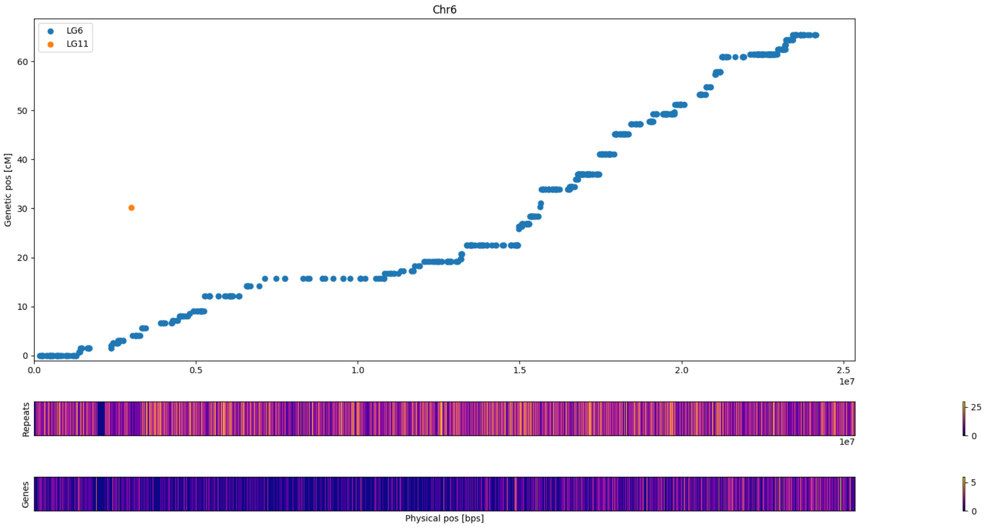

Figure S7: Marey plot of Chr6 with heatmap of Dispersed Repeats and Genes in bins of 200kb. The lighter the colour the more elements are present. Genetic positions refer to the high-density map of Bartlett. Dots represent the genetic and physical position (on of BartlettDHv2.0) of 11,474 SNPs.

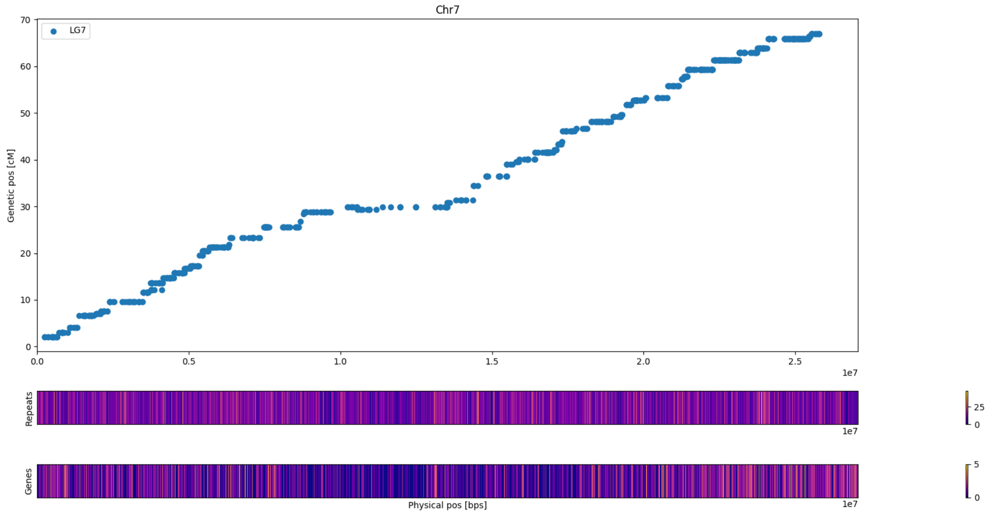

Figure S8: Marey plot of Chr7 with heatmap of Dispersed Repeats and Genes in bins of 200kb. The lighter the colour the more elements are present. Genetic positions refer to the high-density map of Bartlett. Dots represent the genetic and physical position (on of BartlettDHv2.0) of 11,474 SNPs.

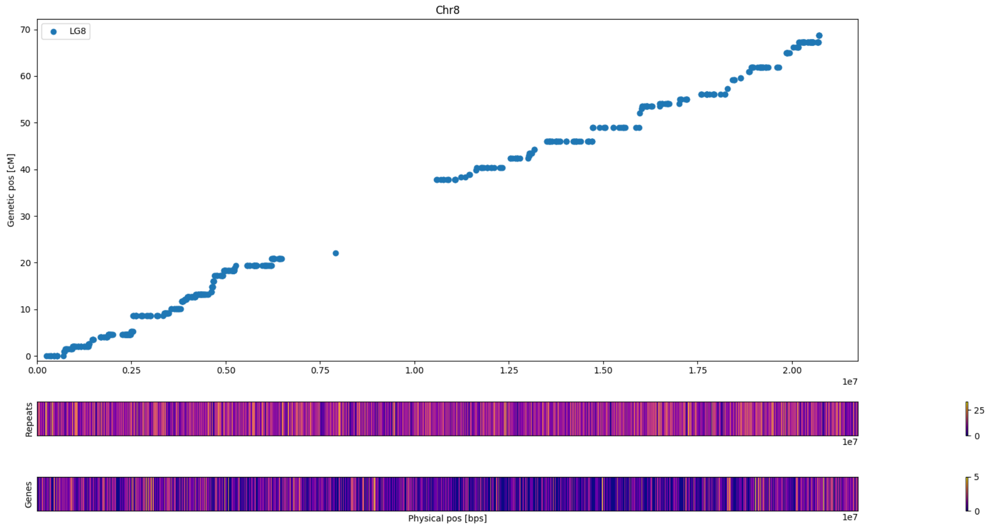

Figure S9: Marey plot of Chr8 with heatmap of Dispersed Repeats and Genes in bins of 200kb. The lighter the colour the more elements are present. Genetic positions refer to the high-density map of Bartlett. Dots represent the genetic and physical position (on of BartlettDHv2.0) of 11,474 SNPs.

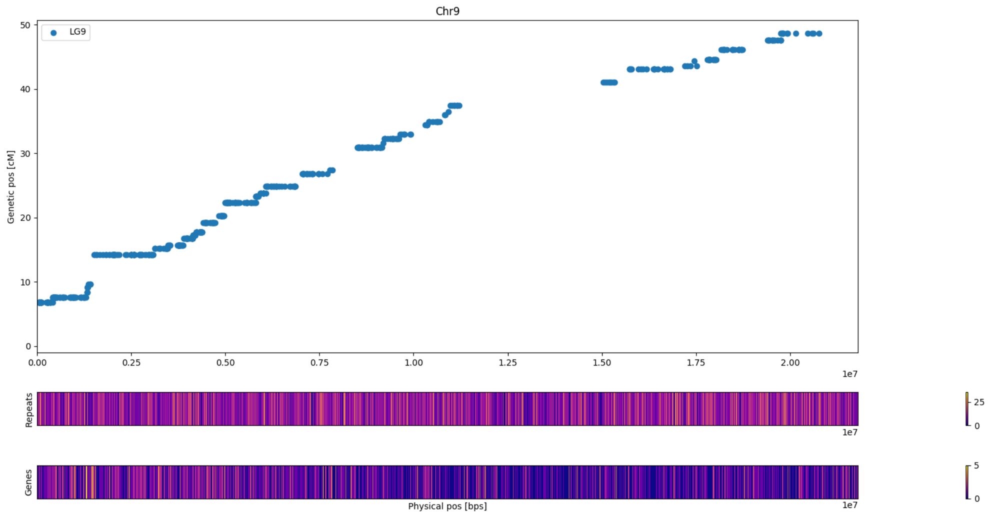

Figure S10: Marey plot of Chr9 with heatmap of Dispersed Repeats and Genes in bins of 200kb. The lighter the colour the more elements are present. Genetic positions refer to the high-density map of Bartlett. Dots represent the genetic and physical position (on of BartlettDHv2.0) of 11,474 SNPs.

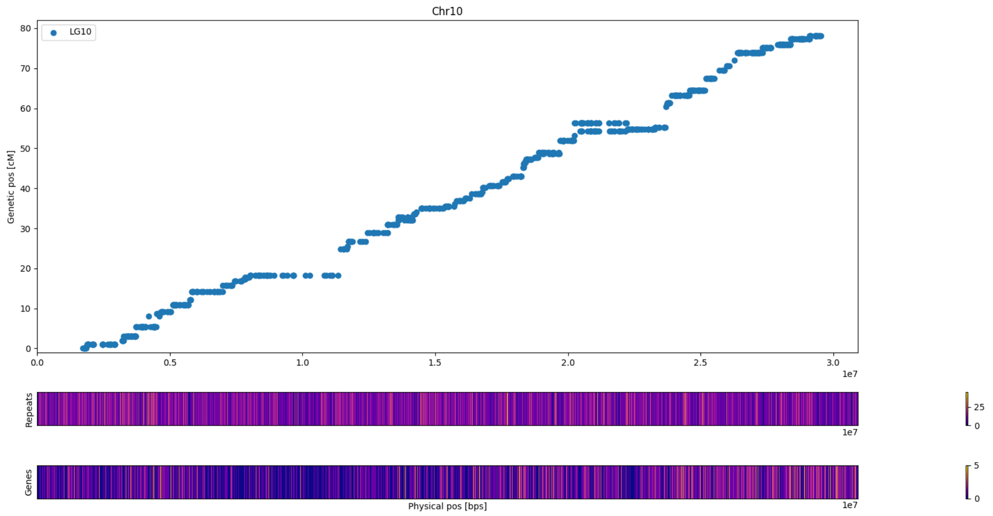

Figure S11: Marey plot of Chr10 with heatmap of Dispersed Repeats and Genes in bins of 200kb. The lighter the colour the more elements are present. Genetic positions refer to the high-density map of Bartlett. Dots represent the genetic and physical position (on of BartlettDHv2.0) of 11,474 SNPs.

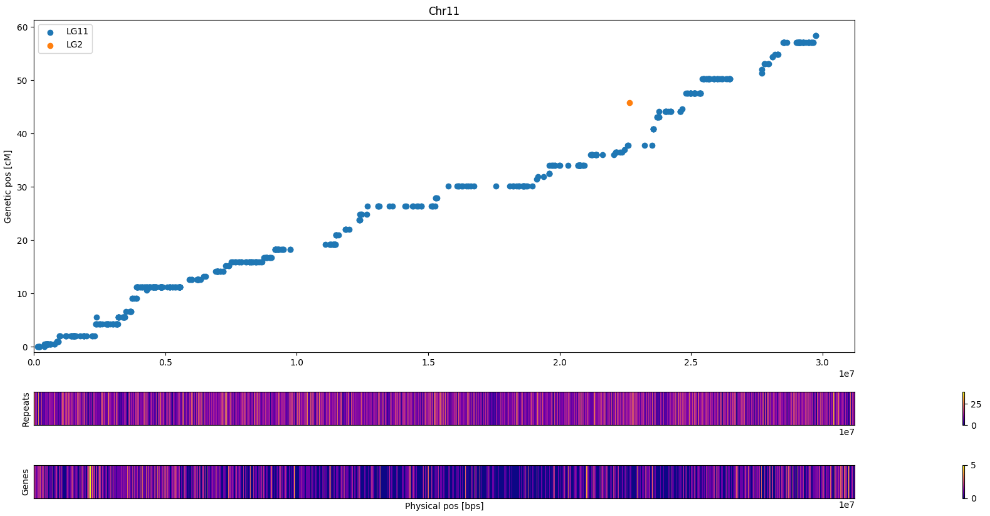

Figure S12: Marey plot of Chr11 with heatmap of Dispersed Repeats and Genes in bins of 200kb. The lighter the colour the more elements are present. Genetic positions refer to the high-density map of Bartlett. Dots represent the genetic and physical position (on of BartlettDHv2.0) of 11,474 SNPs.

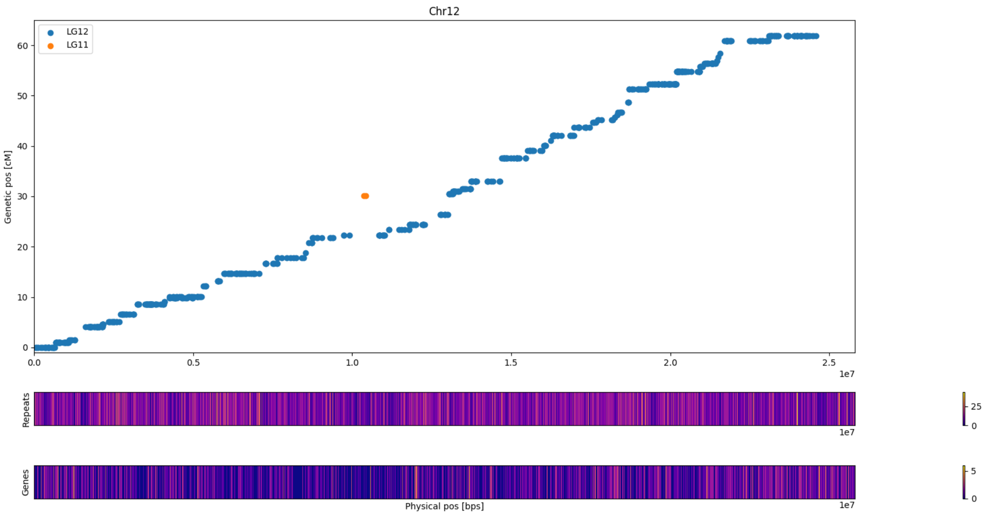

Figure S13: Marey plot of Chr12 with heatmap of Dispersed Repeats and Genes in bins of 200kb. The lighter the colour the more elements are present. Genetic positions refer to the high-density map of Bartlett. Dots represent the genetic and physical position (on of BartlettDHv2.0) of 11,474 SNPs.

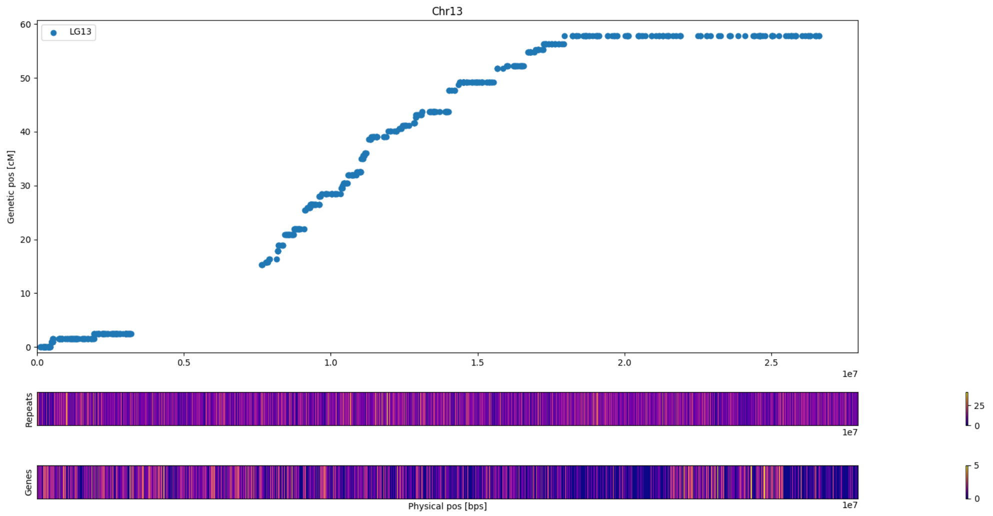

Figure S14: Marey plot of Chr13 with heatmap of Dispersed Repeats and Genes in bins of 200kb. The lighter the colour the more elements are present. Genetic positions refer to the high-density map of Bartlett. Dots represent the genetic and physical position (on of BartlettDHv2.0) of 11,474 SNPs.

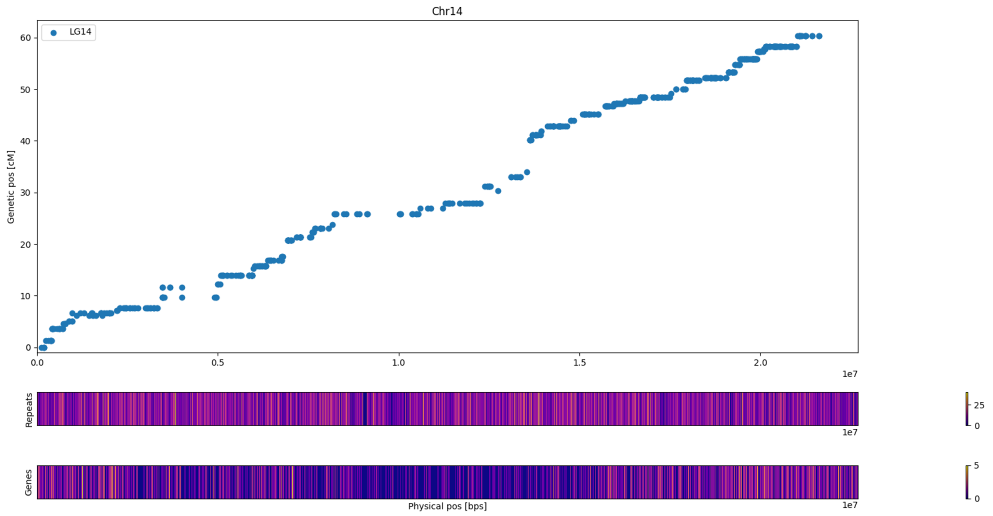

Figure S15: Marey plot of Chr14 with heatmap of Dispersed Repeats and Genes in bins of 200kb. The lighter the colour the more elements are present. Genetic positions refer to the high-density map of Bartlett. Dots represent the genetic and physical position (on of BartlettDHv2.0) of 11,474 SNPs.

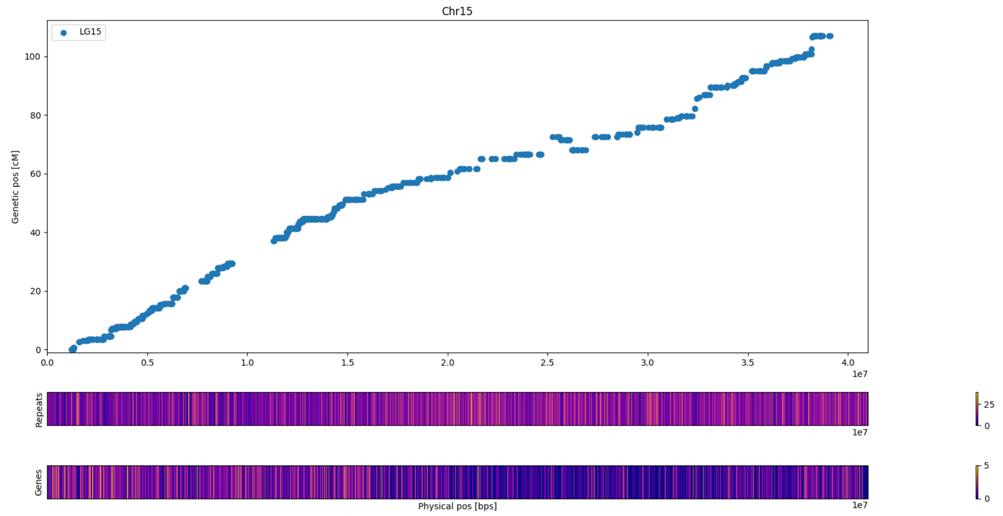

Figure S16: Marey plot of Chr15 with heatmap of Dispersed Repeats and Genes in bins of 200kb. The lighter the colour the more elements are present. Genetic positions refer to the high-density map of Bartlett. Dots represent the genetic and physical position (on of BartlettDHv2.0) of 11,474 SNPs.

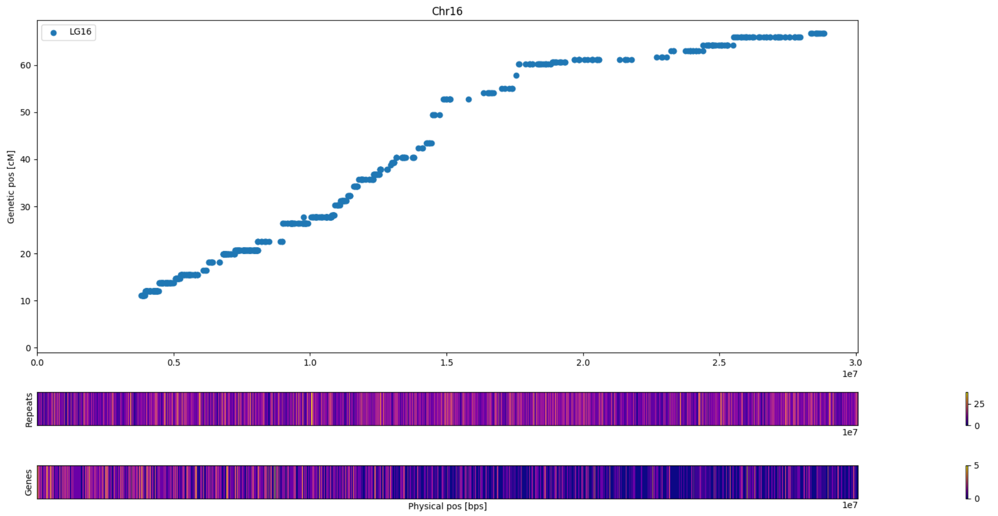

Figure S17: Marey plot of Chr16 with heatmap of Dispersed Repeats and Genes in bins of 200kb. The lighter the colour the more elements are present. Genetic positions refer to the high-density map of Bartlett. Dots represent the genetic and physical position (on of BartlettDHv2.0) of 11,474 SNPs.

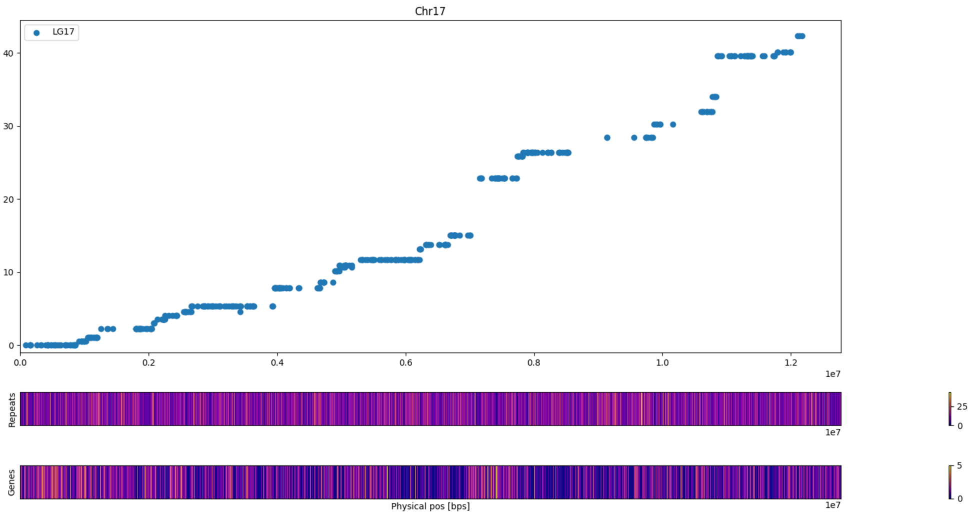

Figure S18: Marey plot of Chr17 with heatmap of Dispersed Repeats and Genes in bins of 200kb. The lighter the colour the more elements are present. Genetic positions refer to the high-density map of Bartlett. Dots represent the genetic and physical position (on of BartlettDHv2.0) of 11,474 SNPs. This Marey plot only shows data for the first 12Mbps of Chr17. Markers in the remaining region show a high level of segregation distortion (and therefore were not mapped genetically) that should be further investigated.

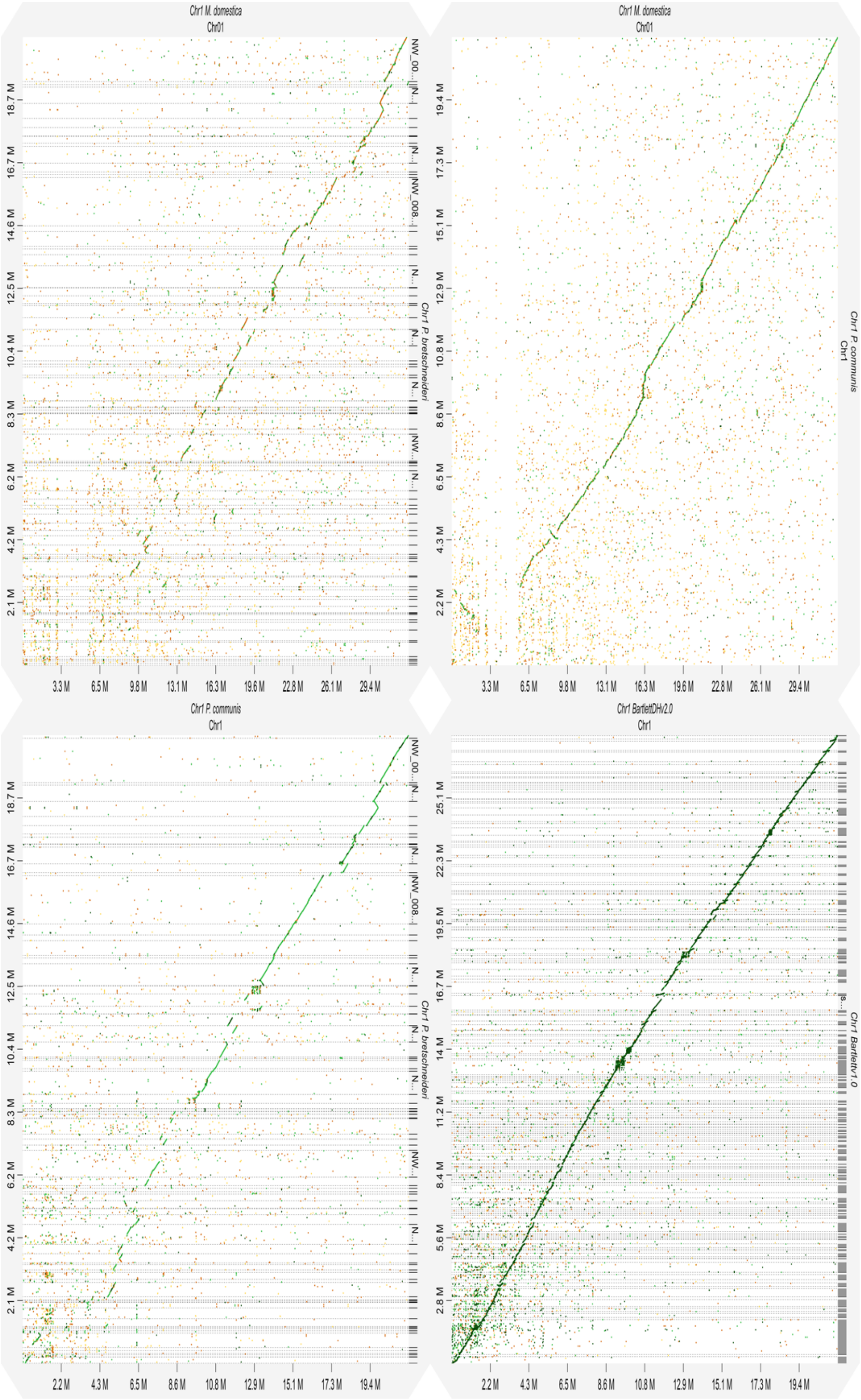

Figure S19. Chromosome 1 alignment dot plots. Dot plots are produced using the DGENIE software[1] and alignments with minimap2 (v2.16).

(Fig S18a) Alignment of Chromosome 1 *P. x bretschneideri* to *M. domestica* (top left)

(Fig S18b) Alignment of Chromosome 1 *P. communis* to *M. domestica* (top right)

(Fig S18c) Alignment of Chromosome 1 *P. x bretschneideri* to *P. communis* (bottom left)

(Fig S18d) Alignment of Chromosome 1 *P. communis* of Bartlettv1.0 to of BartlettDHv2.0

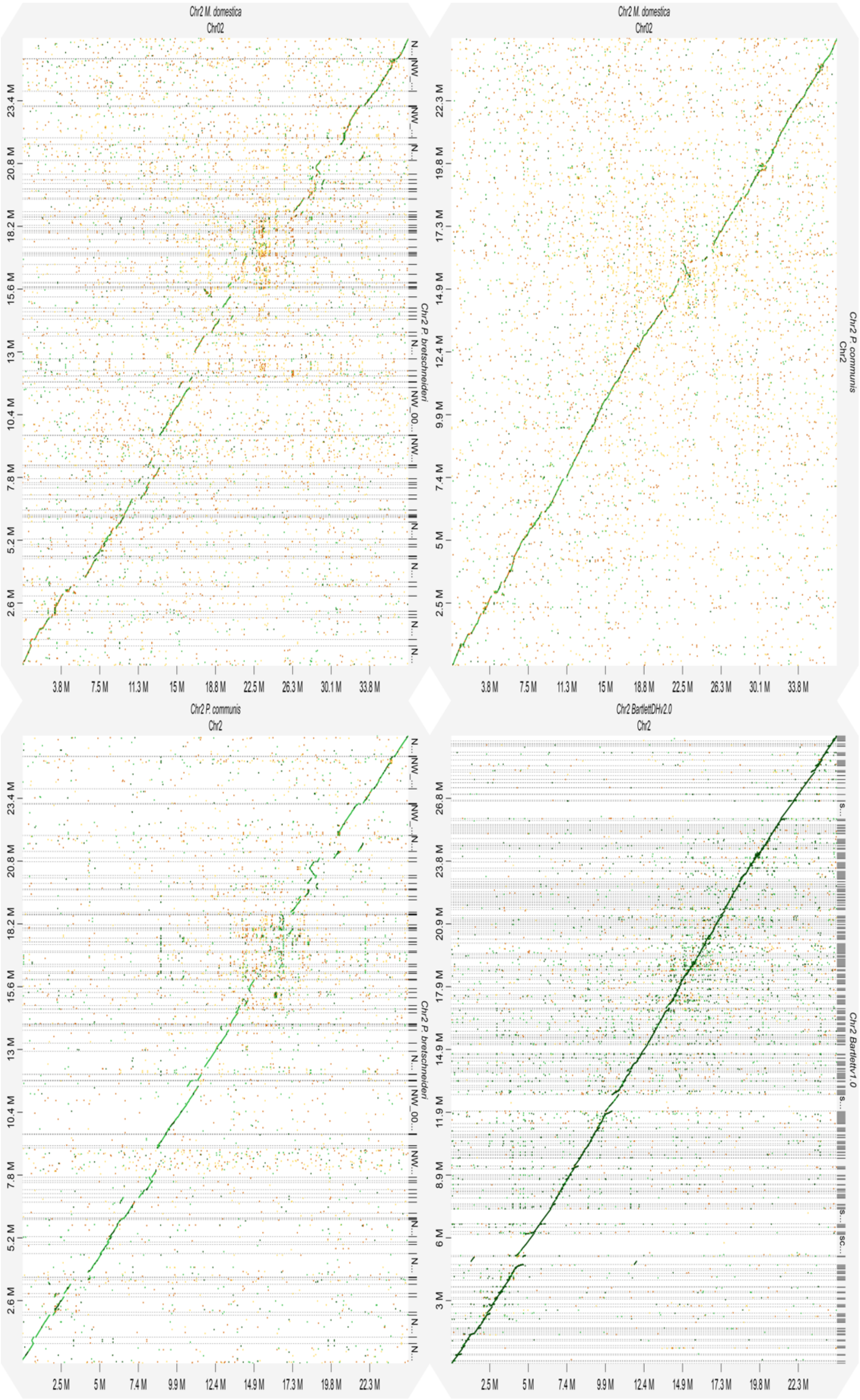

Figure S20. Chromosome 2 alignment dot plots. Dot plots are produced using the DGENIE software[1] and alignments with minimap2 (v2.16).

(Fig S19a) Alignment of Chromosome 2 *P. x bretschneideri* to *M. domestica* (top left)

(Fig S19b) Alignment of Chromosome 2 *P. communis* to *M. domestica* (top right)

(Fig S19c) Alignment of Chromosome 2 *P. x bretschneideri* to *P. communis* (bottom left)

(Fig S19d) Alignment of Chromosome 2 *P. communis* of Bartlettv1.0 to of BartlettDHv2.0

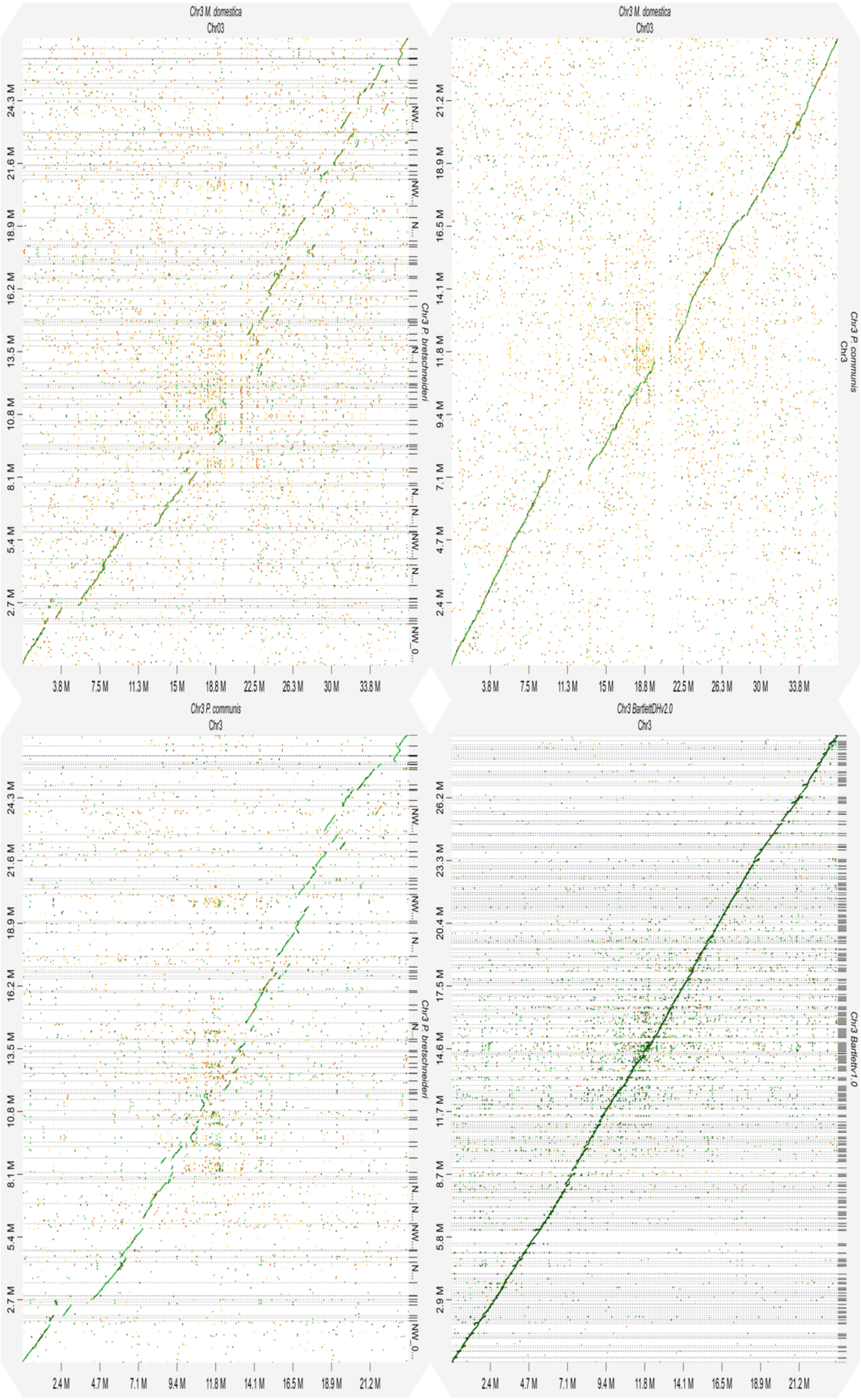

Figure S21. Chromosome 3 alignment dot plots. Dot plots are produced using the DGENIE software[1] and alignments with minimap2 (v2.16).

(Fig S20a) Alignment of Chromosome 3 *P. x bretschneideri* to *M. domestica* (top left)

(Fig S20b) Alignment of Chromosome 3 *P. communis* to *M. domestica* (top right)

(Fig S20c) Alignment of Chromosome 3 *P. x bretschneideri* to *P. communis* (bottom left)

(Fig S20d) Alignment of Chromosome 3 *P. communis* of Bartlettv1.0 to of BartlettDHv2.0

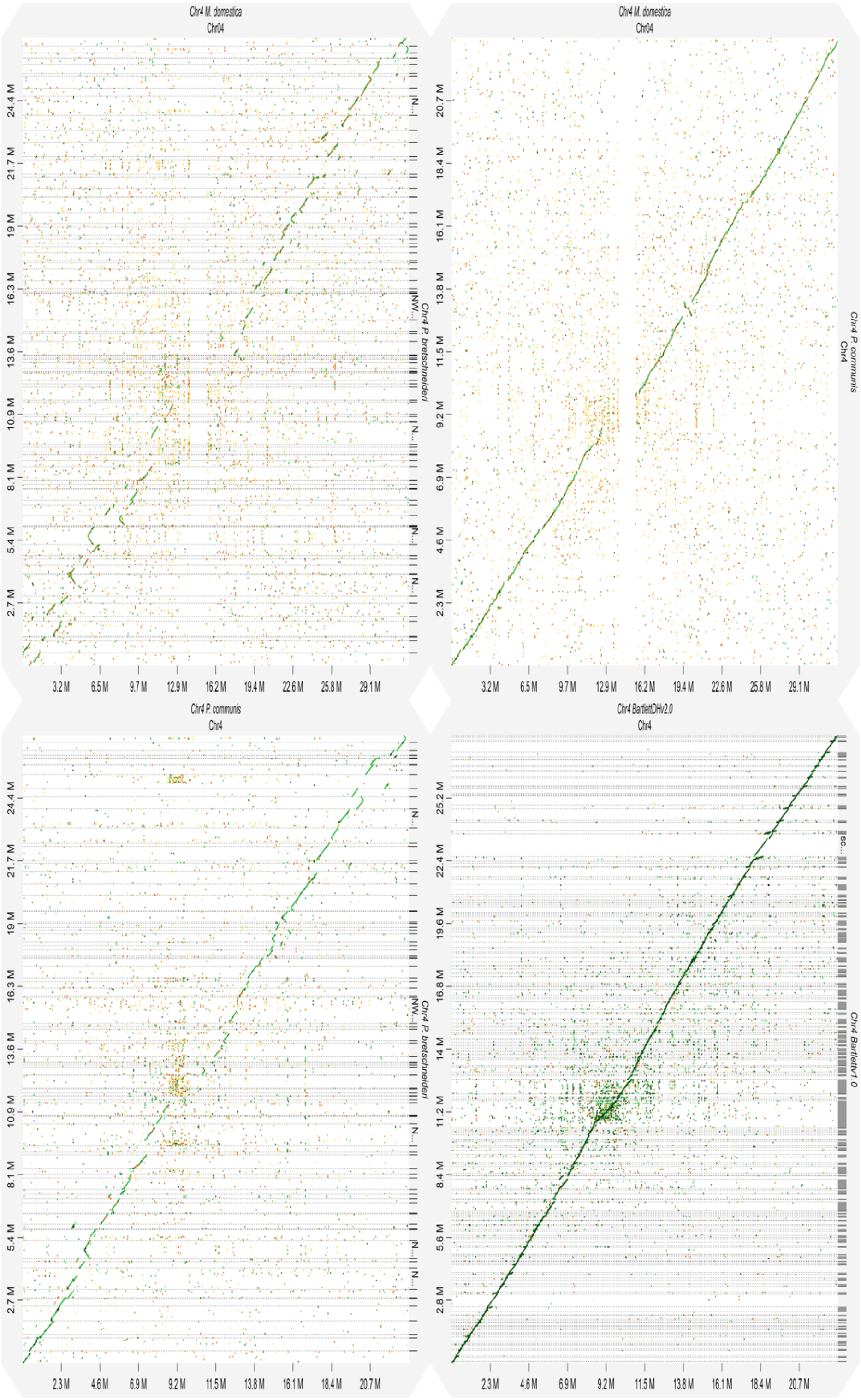

Figure S22. Chromosome 4 alignment dot plots. Dot plots are produced using the DGENIE software[1]and alignments with minimap2 (v2.16).

(Fig S21a) Alignment of Chromosome 4 *P. x bretschneideri* to *M. domestica* (top left)

(Fig S21b) Alignment of Chromosome 4 *P. communis* to *M. domestica* (top right)

(Fig S21c) Alignment of Chromosome 4 *P. x bretschneideri* to *P. communis* (bottom left)

(Fig S21d) Alignment of Chromosome 4 *P. communis* of Bartlettv1.0 to of BartlettDHv2.0

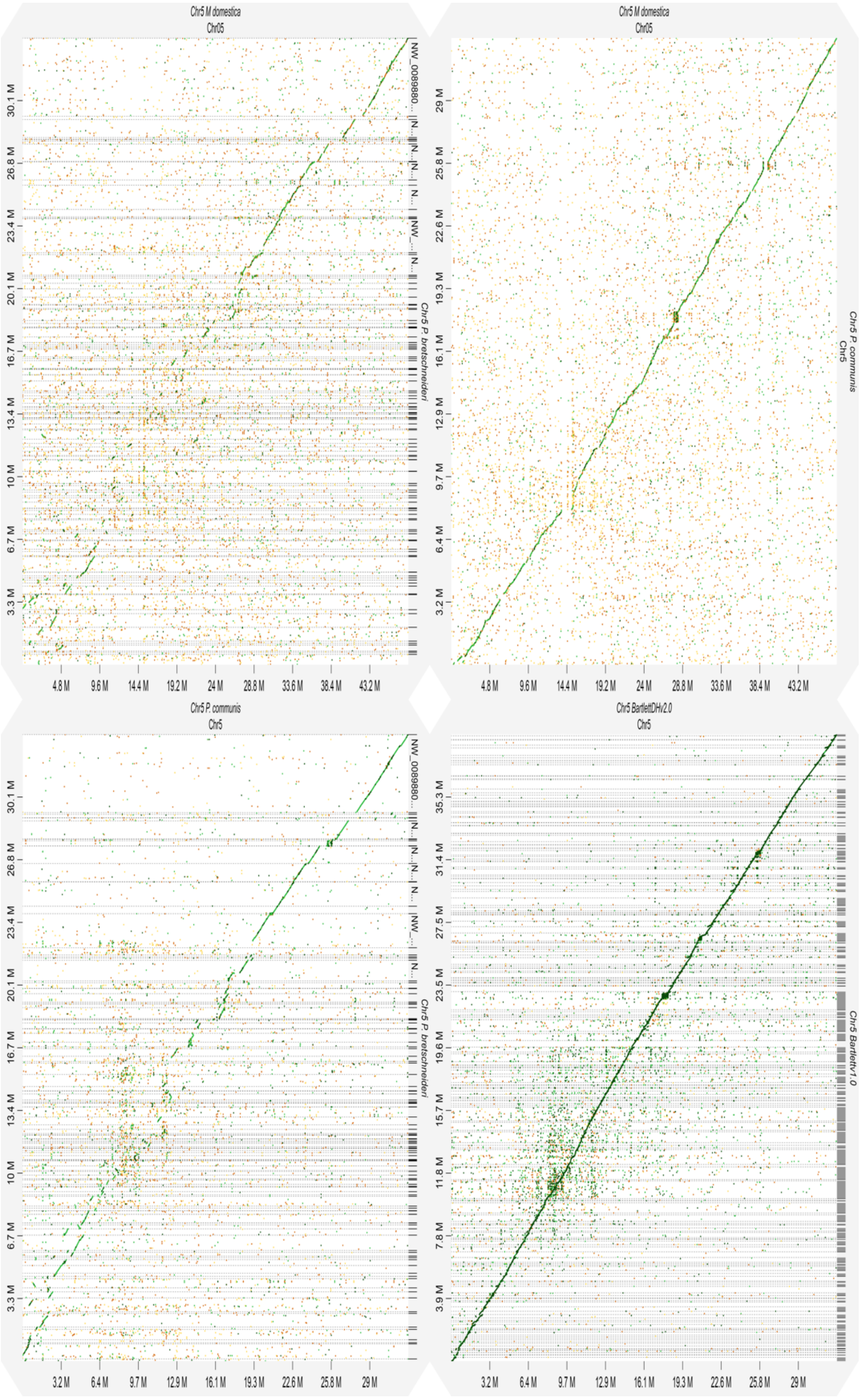

Figure S23. Chromosome 5 alignment dot plots. Dot plots are produced using the DGENIE software[1] and alignments with minimap2 (v2.16).

(Fig S22a) Alignment of Chromosome 5 *P. x bretschneideri* to *M. domestica* (top left)

(Fig S22b) Alignment of Chromosome 5 *P. communis* to *M. domestica* (top right)

(Fig S22c) Alignment of Chromosome 5 *P. x bretschneideri* to *P. communis* (bottom left)

(Fig S22d) Alignment of Chromosome 5 *P. communis* of Bartlettv1.0 to of BartlettDHv2.0

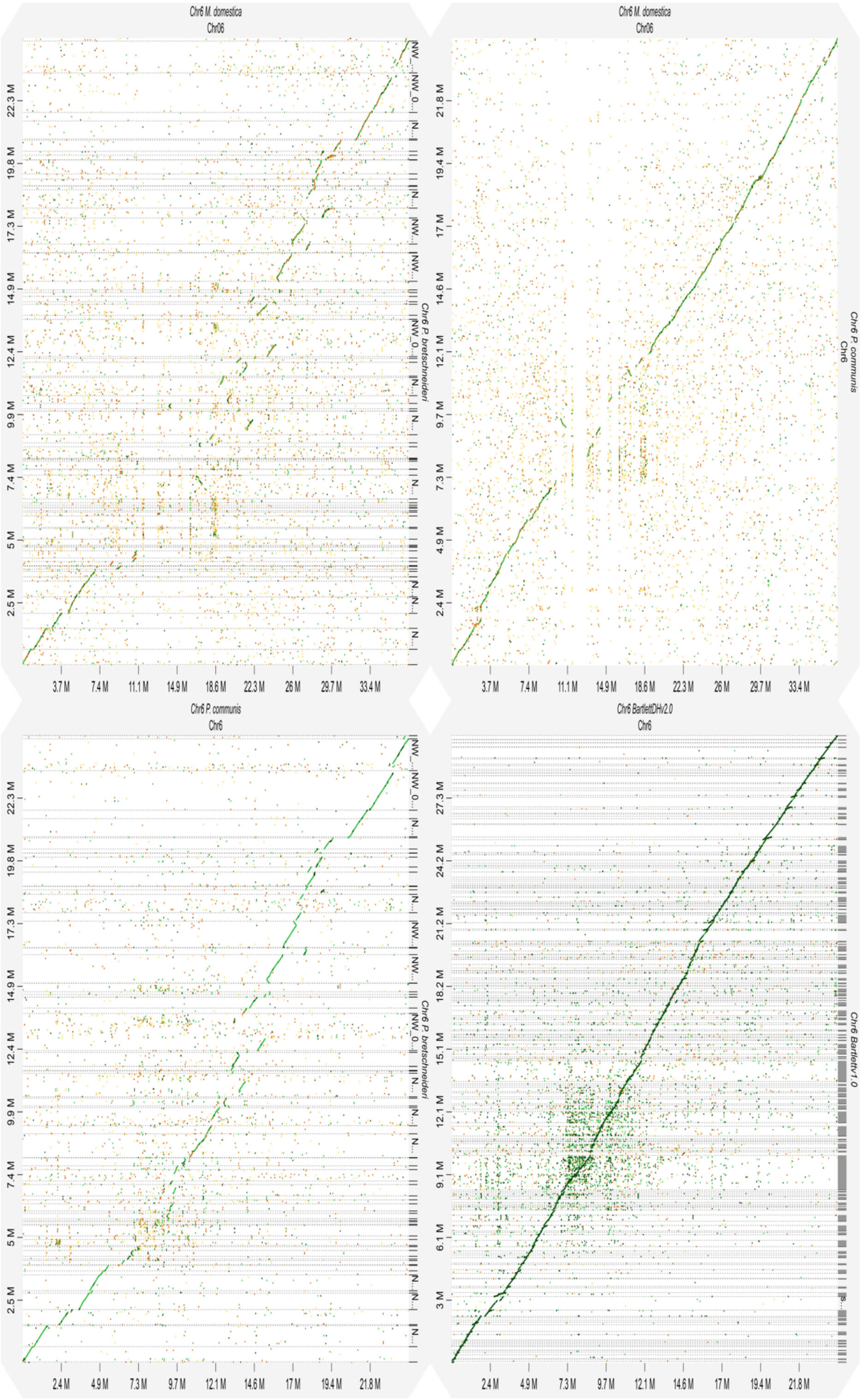

Figure S24. Chromosome 6 alignment dot plots. Dot plots are produced using the DGENIE software[1] and alignments with minimap2 (v2.16).

(Fig S23a) Alignment of Chromosome 6 *P. x bretschneideri* to *M. domestica* (top left)

(Fig S23b) Alignment of Chromosome 6 *P. communis* to *M. domestica* (top right)

(Fig S23c) Alignment of Chromosome 6 *P. x bretschneideri* to *P. communis* (bottom left)

(Fig S23d) Alignment of Chromosome 6 *P. communis* of Bartlettv1.0 to of BartlettDHv2.0

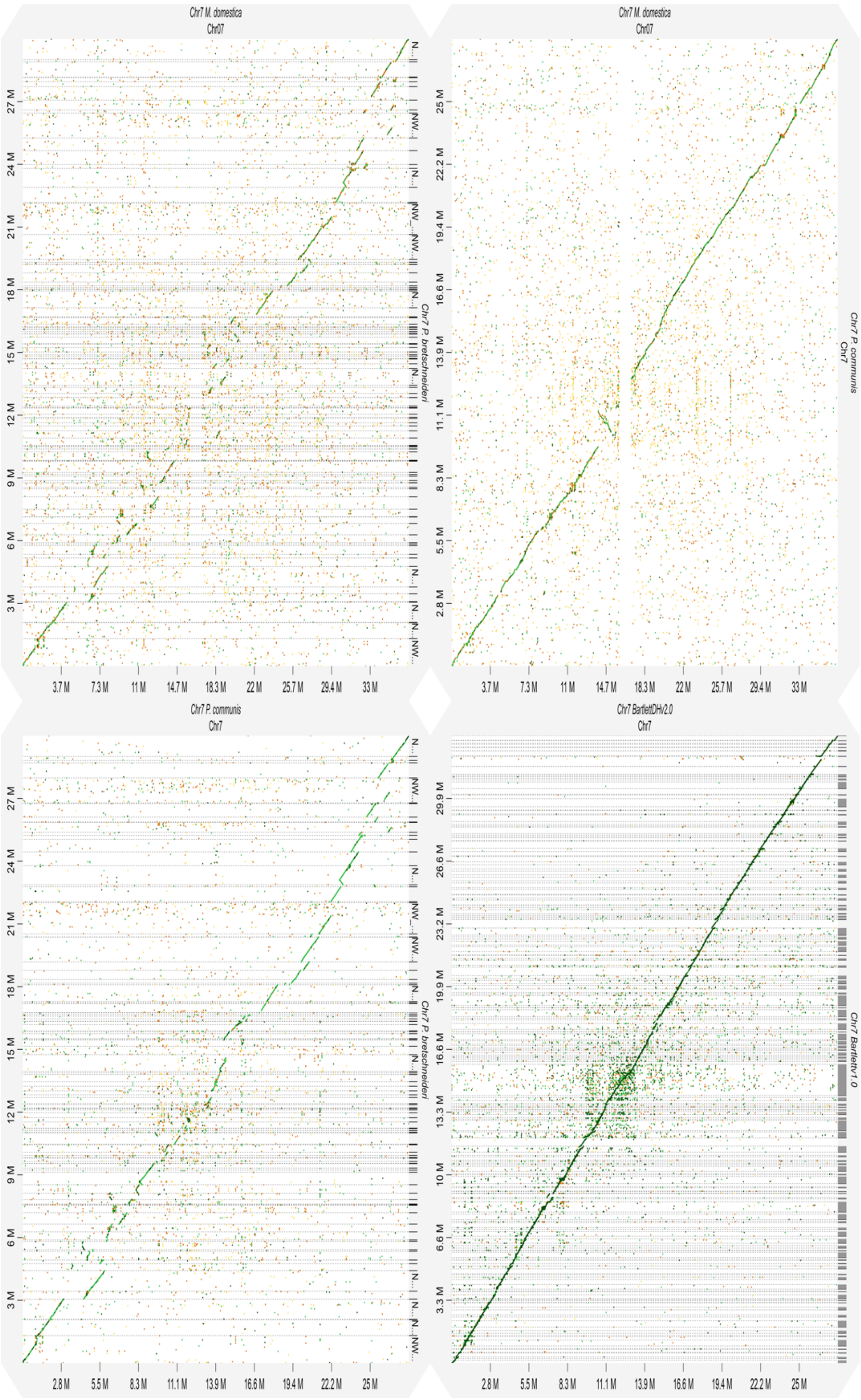

Figure S25. Chromosome 7 alignment dot plots. Dot plots are produced using the DGENIE software[1] and alignments with minimap2 (v2.16).

(Fig S24a) Alignment of Chromosome 7 *P. x bretschneideri* to *M. domestica* (top left)

(Fig S24b) Alignment of Chromosome 7 *P. communis* to *M. domestica* (top right)

(Fig S24c) Alignment of Chromosome 7 *P. x bretschneideri* to *P. communis* (bottom left)

(Fig S24d) Alignment of Chromosome 7 *P. communis* of Bartlettv1.0 to of BartlettDHv2.0

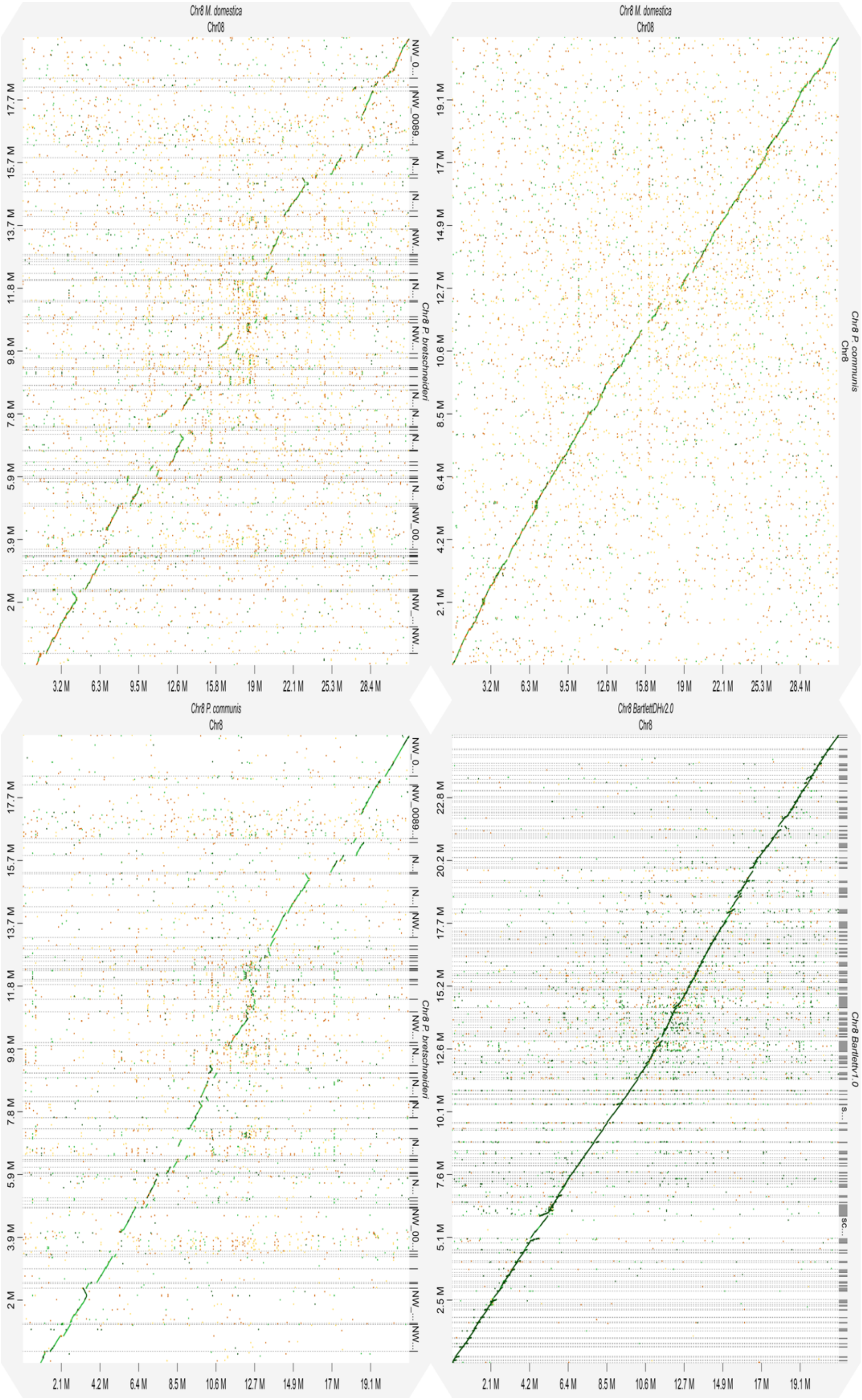

Figure S26. Chromosome 8 alignment dot plots. Dot plots are produced using the DGENIE software[1]and alignments with minimap2 (v2.16).

(Fig S25a) Alignment of Chromosome 8 *P. x bretschneideri* to *M. domestica* (top left)

(Fig S25b) Alignment of Chromosome 8 *P. communis* to *M. domestica* (top right)

(Fig S25c) Alignment of Chromosome 8 *P. x bretschneideri* to *P. communis* (bottom left)

(Fig S25d) Alignment of Chromosome 8 *P. communis* of Bartlettv1.0 to of BartlettDHv2.0

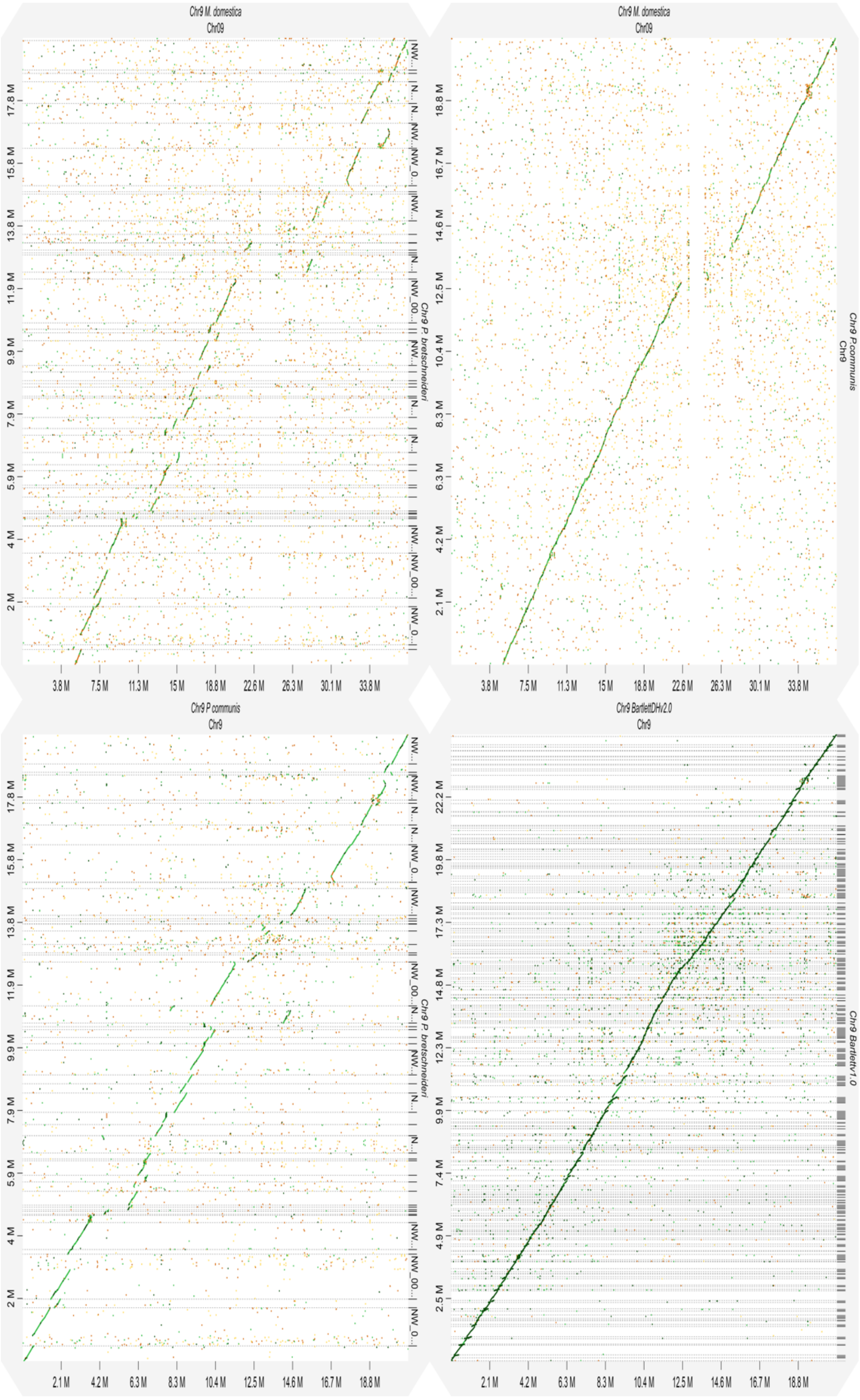

Figure S27. Chromosome 9 alignment dot plots. Dot plots are produced using the DGENIE software[1] and alignments with minimap2 (v2.16).

(Fig S26a) Alignment of Chromosome 9 *P. x bretschneideri* to *M. domestica* (top left)

(Fig S26b) Alignment of Chromosome 9 *P. communis* to *M. domestica* (top right)

(Fig S26c) Alignment of Chromosome 9 *P. x bretschneideri* to *P. communis* (bottom left)

(Fig S26d) Alignment of Chromosome 9 *P. communis* of Bartlettv1.0 to of BartlettDHv2.0

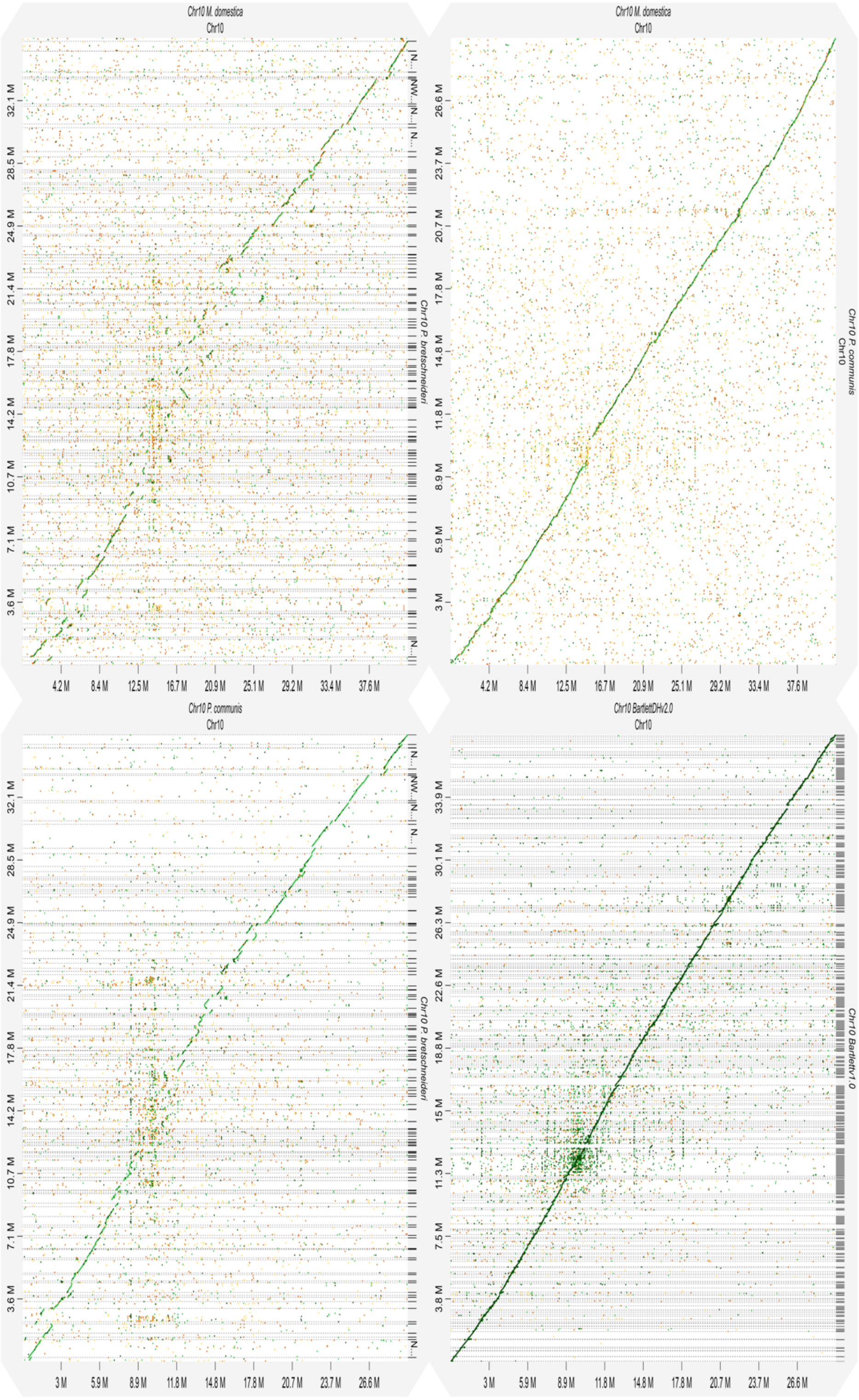

Figure S28. Chromosome 10 alignment alignment dot plots. Dot plots are produced using the DGENIE software[1]and alignments with minimap2 (v2.16).

(Fig S27a) Alignment of Chromosome 10 *P. x bretschneideri* to *M. domestica* (top left)

(Fig S27b) Alignment of Chromosome 10 *P. communis* to *M. domestica* (top right)

(Fig S27c) Alignment of Chromosome 10 *P. x bretschneideri* to *P. communis* (bottom left)

(Fig S27d) Alignment of Chromosome 10 *P. communis* of Bartlettv1.0 to of BartlettDHv2.0

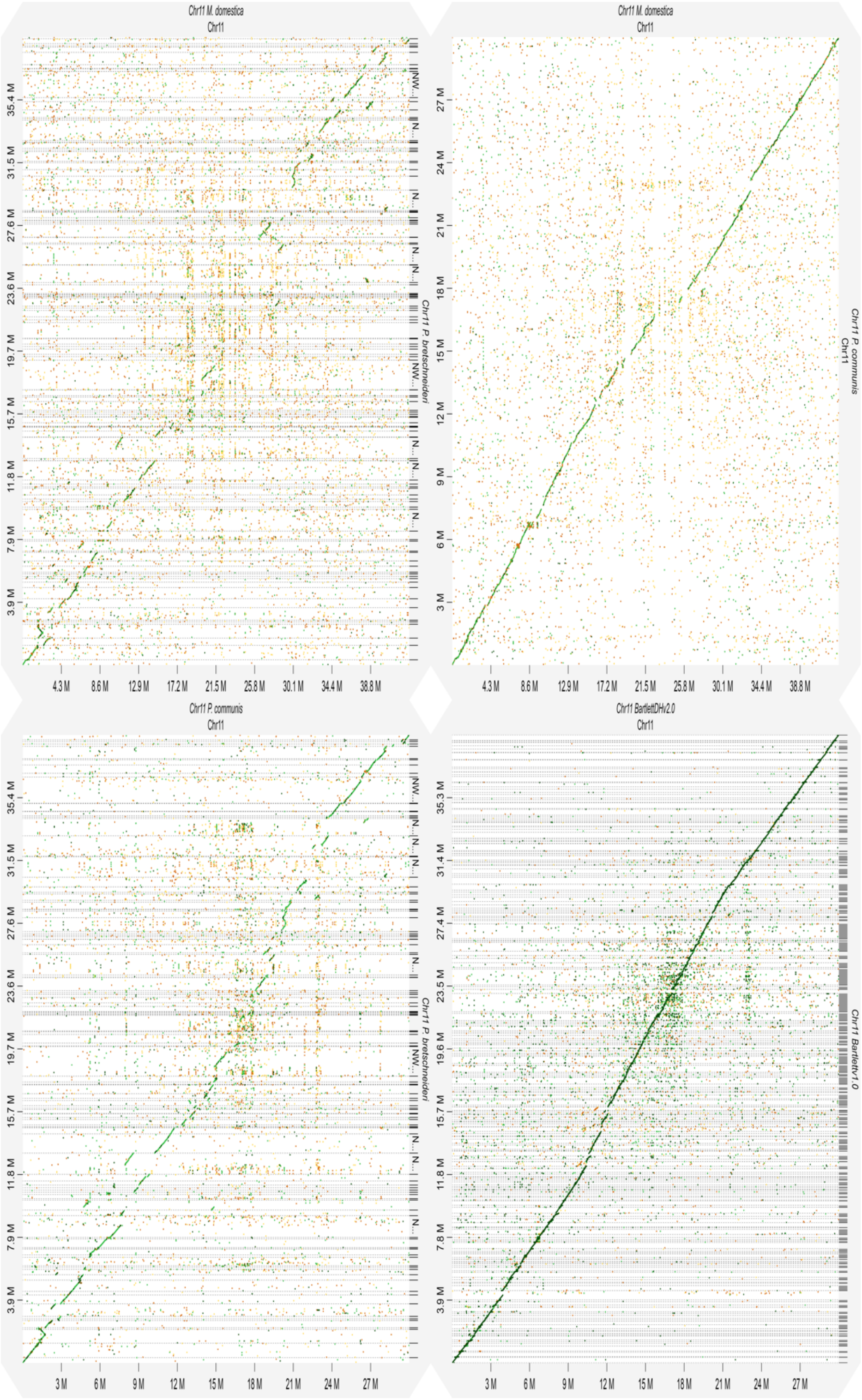

Figure S29. Chromosome 11 alignment alignment dot plots. Dot plots are produced using the DGENIE software[1]and alignments with minimap2 (v2.16).

(Fig S28a) Alignment of Chromosome 11 *P. x bretschneideri* to *M. domestica* (top left)

(Fig S28b) Alignment of Chromosome 11 *P. communis* to *M. domestica* (top right)

(Fig S28c) Alignment of Chromosome 11 *P. x bretschneideri* to *P. communis* (bottom left)

(Fig S28d) Alignment of Chromosome 11 *P. communis* of Bartlettv1.0 to of BartlettDHv2.0

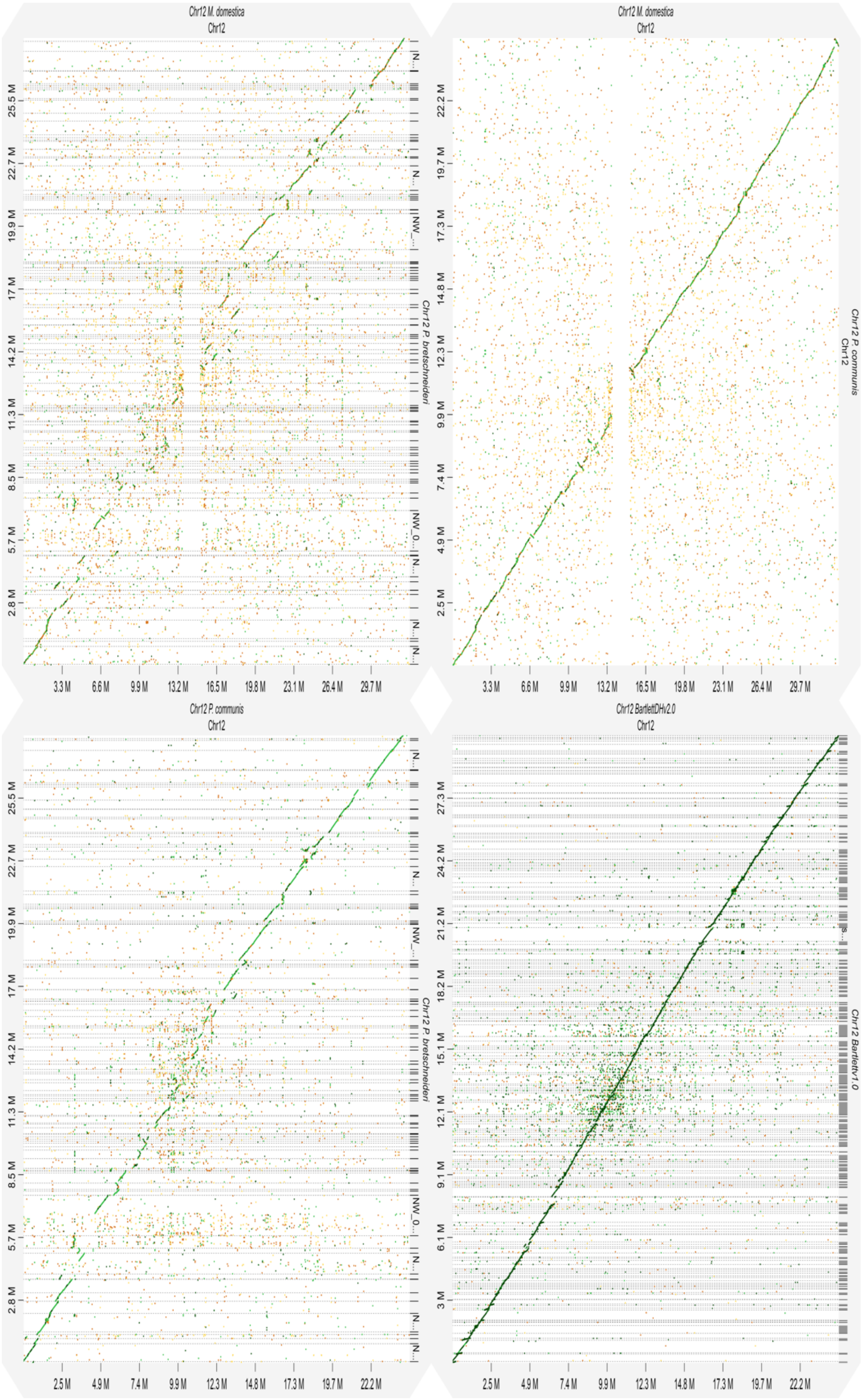

Figure S30. Chromosome 12 alignment alignment dot plots. Dot plots are produced using the DGENIE software[1] and alignments with minimap2 (v2.16).

(Fig S29a) Alignment of Chromosome 12 *P. x bretschneideri* to *M. domestica* (top left)

(Fig S29b) Alignment of Chromosome 12 *P. communis* to *M. domestica* (top right)

(Fig S29c) Alignment of Chromosome 12 *P. x bretschneideri* to P. communis (bottom left)

(Fig S29d) Alignment of Chromosome 12 *P. communis* of Bartlettv1.0 to of BartlettDHv2.0

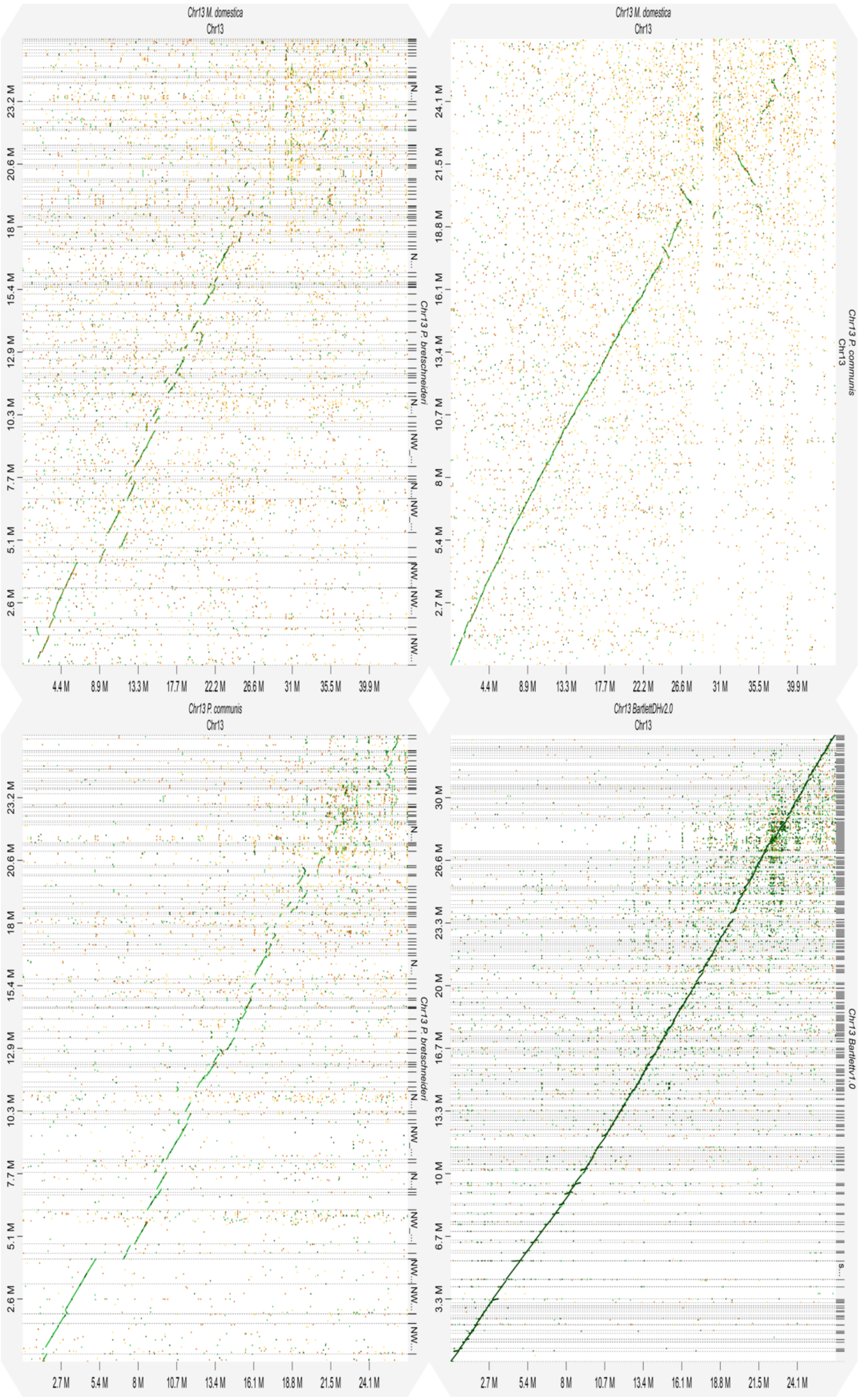

Figure S31. Chromosome 13 alignment alignment dot plots. Dot plots are produced using the DGENIE software[1] and alignments with minimap2 (v2.16).

(Fig S30a) Alignment of Chromosome 13 *P. x bretschneideri* to *M. domestica* (top left)

(Fig S30b) Alignment of Chromosome 13 *P. communis* to *M. domestica* (top right)

(Fig S30c) Alignment of Chromosome 13 *P. x bretschneideri* to *P. communis* (bottom left)

(Fig S30d) Alignment of Chromosome 13 *P. communis* of Bartlettv1.0 to of BartlettDHv2.0

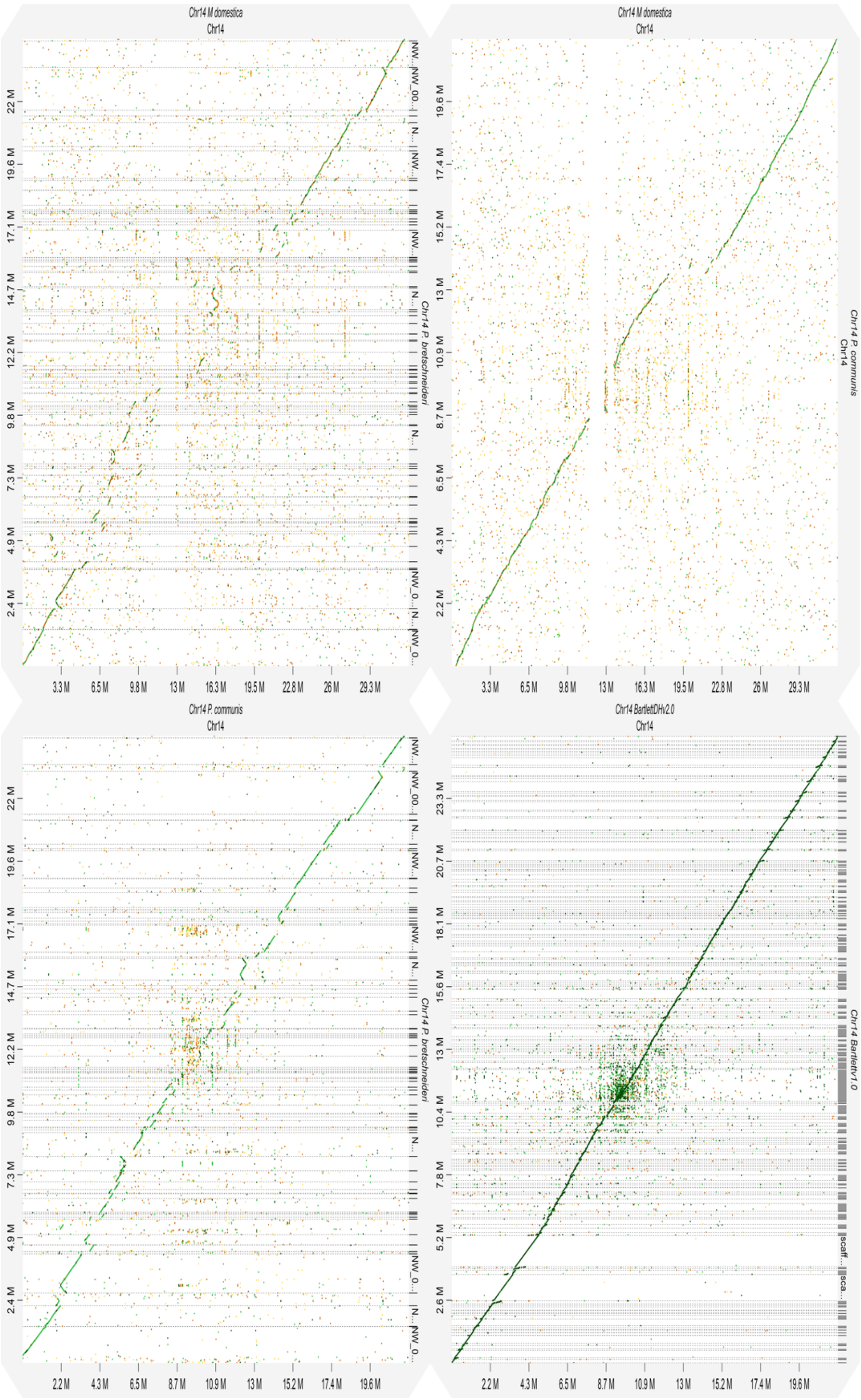

Figure S32. Chromosome 14 alignment alignment dot plots. Dot plots are produced using the DGENIE software[1]and alignments with minimap2 (v2.16).

(Fig S31a) Alignment of Chromosome 14 *P. x bretschneideri* to *M. domestica* (top left)

(Fig S31b) Alignment of Chromosome 14 *P. communis* to *M. domestica* (top right)

(Fig S31c) Alignment of Chromosome 14 *P. x bretschneideri* to *P. communis* (bottom left)

(Fig S31d) Alignment of Chromosome 14 *P. communis* of Bartlettv1.0 to of BartlettDHv2.0

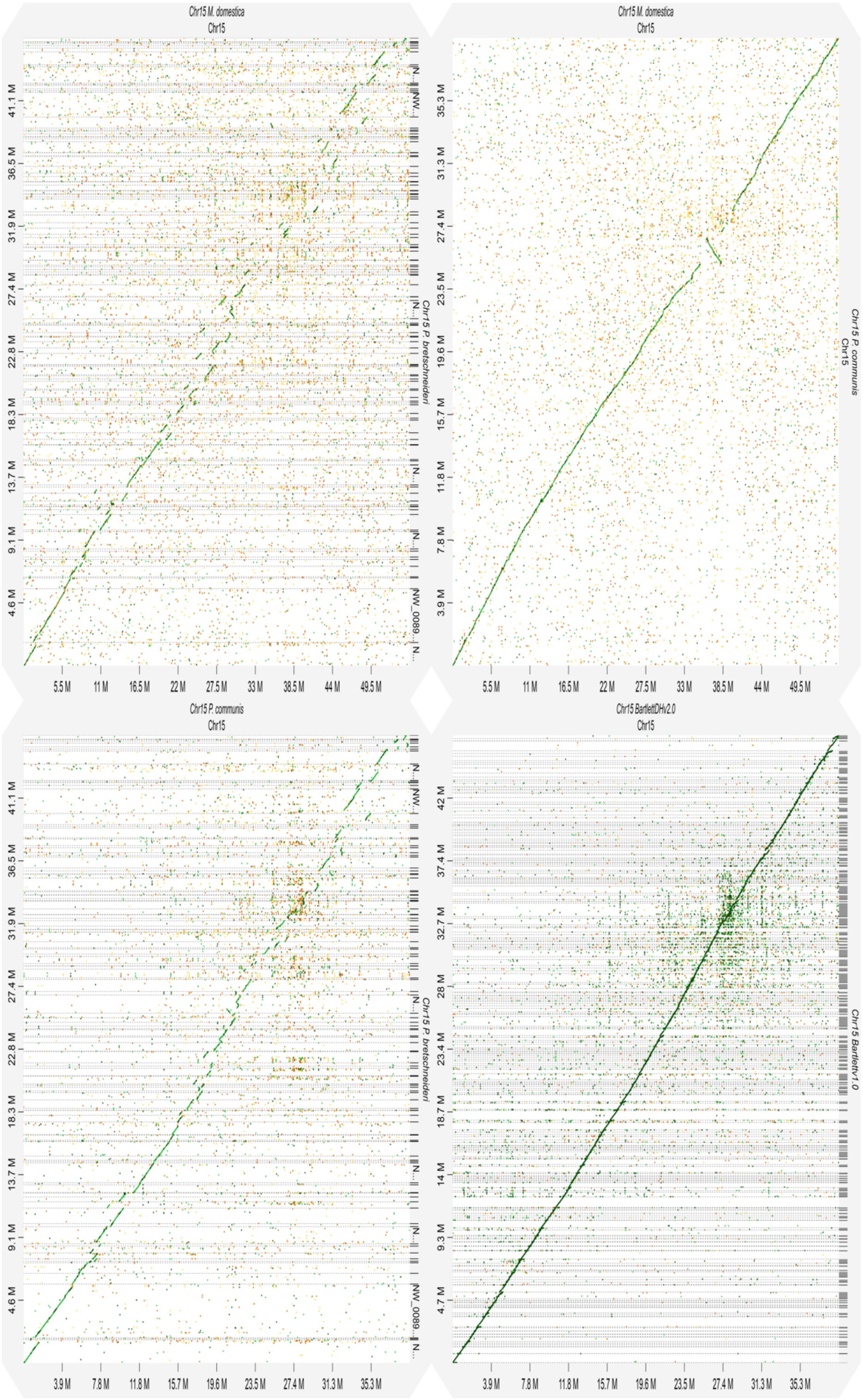

Figure S33. Chromosome 15 alignment alignment dot plots. Dot plots are produced using the DGENIE software[1] and alignments with minimap2 (v2.16).

(Fig S32a) Alignment of Chromosome 15 *P. x bretschneideri* to *M. domestica* (top left)

(Fig S32b) Alignment of Chromosome 15 *P. communis* to *M. domestica* (top right)

(Fig S32c) Alignment of Chromosome 15 *P. x bretschneideri* to *P. communis* (bottom left)

(Fig S32d) Alignment of Chromosome 15 *P. communis* of Bartlettv1.0 to of BartlettDHv2.0

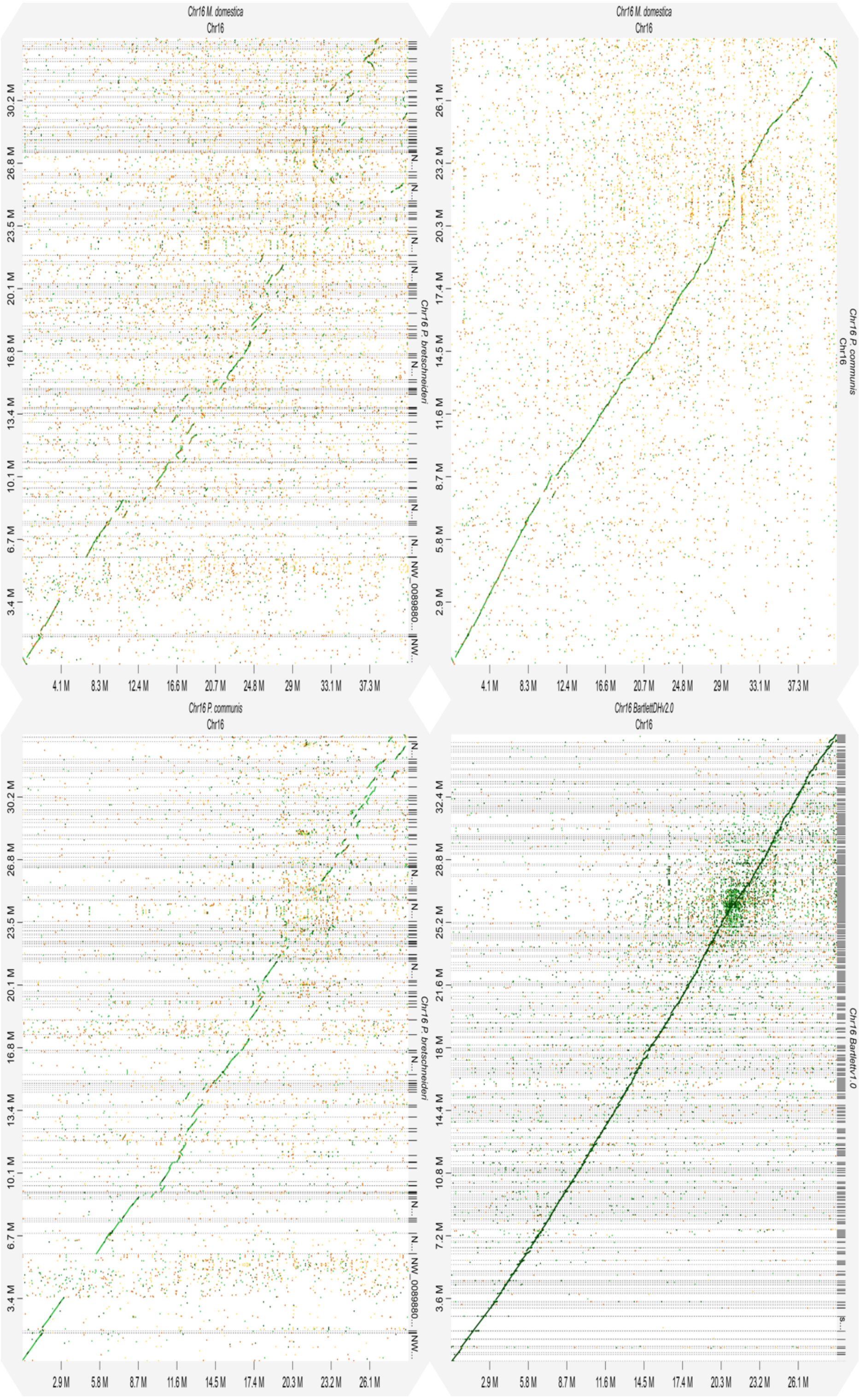

Figure S34. Chromosome 16 alignment alignment dot plots. Dot plots are produced using the DGENIE software[1] and alignments with minimap2 (v2.16).

(Fig S33a) Alignment of Chromosome 16 *P. x bretschneideri* to *M. domestica* (top left)

(Fig S33b) Alignment of Chromosome 16 *P. communis* to *M. domestica* (top right)

(Fig S33c) Alignment of Chromosome 16 *P. x bretschneideri* to *P. communis* (bottom left)

(Fig S33d) Alignment of Chromosome 16 *P. communis* of Bartlettv1.0 to of BartlettDHv2.0

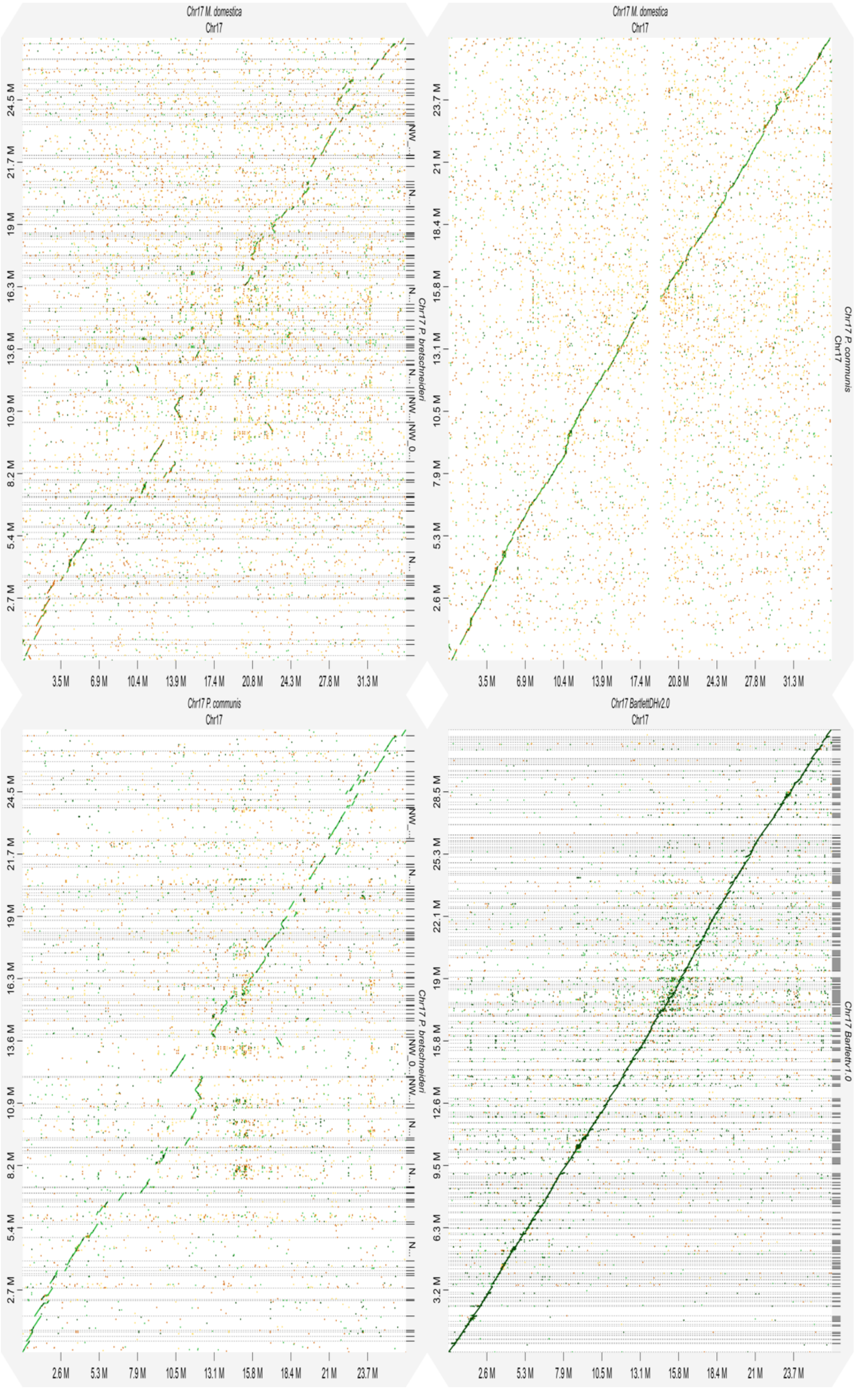

Figure S35. Chromosome 17 alignment alignment dot plots. Dot plots are produced using the DGENIE software[1] and alignments with minimap2 (v2.16).

(Fig S34a) Alignment of Chromosome 17 *P. x bretschneideri* to *M. domestica* (top left)

(Fig S34b) Alignment of Chromosome 17 *P. communis* to *M. domestica* (top right)

(Fig S34c) Alignment of Chromosome 17 *P. x bretschneideri* to *P. communis* (bottom left)

(Fig S34d) Alignment of Chromosome 17 *P. communis* of Bartlettv1.0 to of BartlettDHv2.

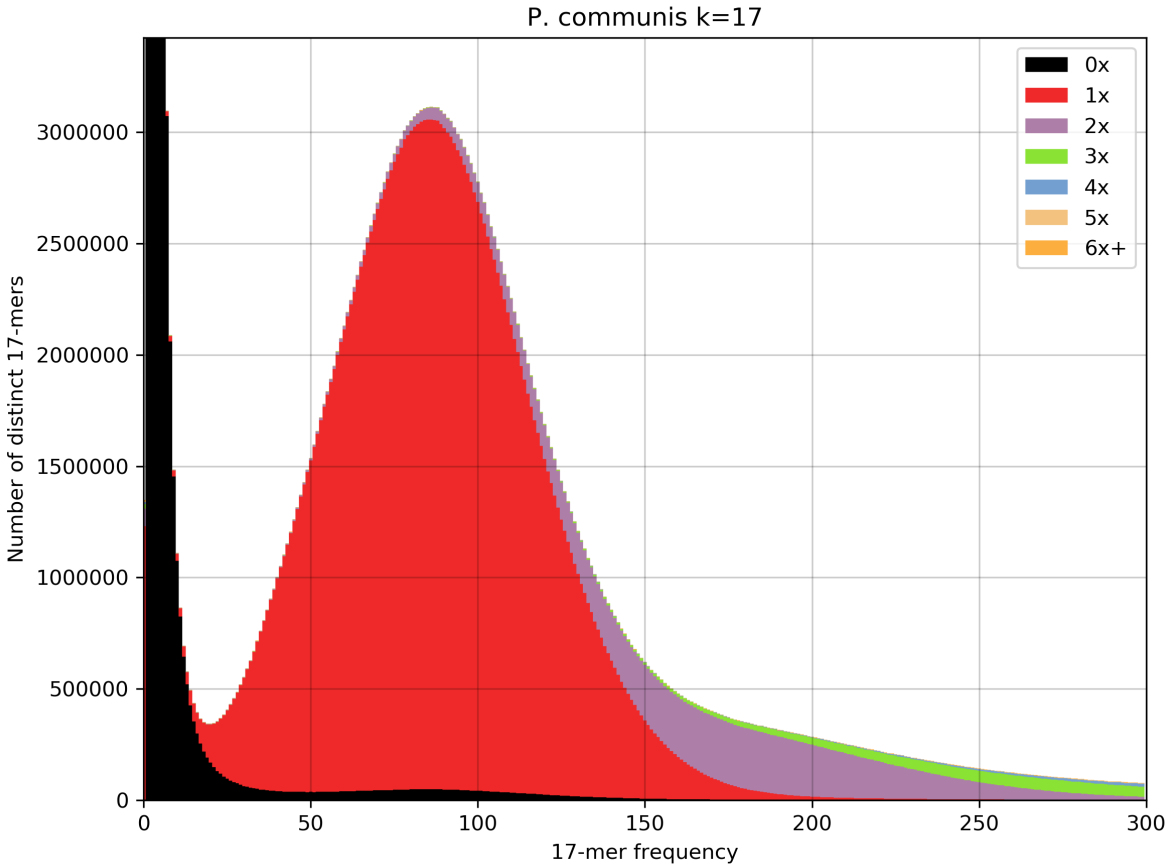

Figure S36. Kmer spectrum copy number plot for BartlettDHv2 *(k=17).*

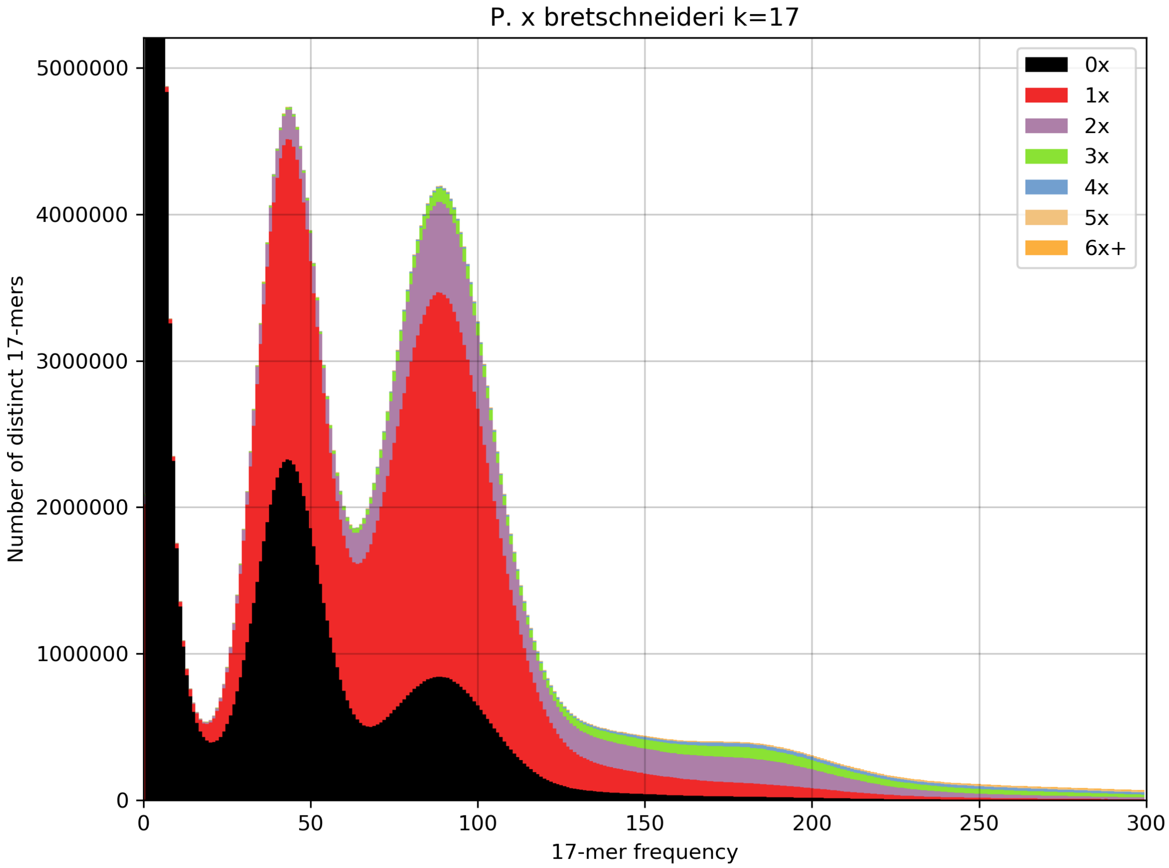

Figure S37. Kmer spectrum copy number plot for *P. × bretschneideri (k=17).*

Kmer comparison with Illumina data

As an additional QA measure the kmer content of the assembly was compared to that of the Illumina data using the Kmer analysis toolkit[31]. Using KAT[31] v2.3.4, (KAT comp tool) 17-mers were counted in all whole genome shotgun paired-end read and also in the BartlettDHv2 assembly. The kmer spectrum of the reads is then annotated with colours to denote the proportion of kmers with that frequency occurring once, twice, three times, etc. in the assembly (Figure S36). It is evident from this plot that very little of the content which should appear once in the genome (content from the homozygous single copy peak at 86X) is duplicated in the assembly (purple part of the single copy peak at 86X). The vast majority of kmers that appear twice in the assembly (purple coloured parts of the figure) are in the small bump in the Illumina kmer spectrum centred at 172X i.e. they are kmers from the duplicated part of the genome. By comparison the picture for the heterozygous *P. x bretschneideri* (Figure S37) is much more complicated. There are many kmers from the main peak appearing two or three times in the assembly and there are also many kmers missing from the assembly. These features likely represent a mixture of duplicated content and kmers from duplicated heterozygous regions which have been successfully collapsed to a haploid consensus sequence. This demonstrates the added complexity involved in assembling a heterozygous sample.

Enriched GO terms

Table S1 GO terms enriched in *P. communis* genes determined to be pomme specific

414 pomme specific gene families containing 1,447 *P. communis* genes were enriched for the following GO terms.

#Type	GO-id	Enrichment	p-value	subset-ratio	description
MF	GO:0001882	0.411353449	6.16E-04	25.03037667	nucleoside binding
MF	GO:0001883	0.409747787	7.06E-04	24.90886999	purine nucleoside binding
MF	GO:0003824	0.259594027	9.57E-07	59.29526124	catalytic activity
MF	GO:0004872	0.98725488	0.038161026	4.009720535	receptor activity
MF	GO:0004888	0.98725488	0.038161026	4.009720535	transmembrane receptor activity
MF	GO:0005215	0.595920778	0.043424568	8.991494532	transporter activity
MF	GO:0005216	1.580096674	0.010753402	2.187120292	ion channel activity
MF	GO:0005524	0.462925951	7.16E-05	24.54434994	ATP binding
MF	GO:0015075	1.066765598	2.41E-04	5.953827461	ion transmembrane transporter activity
MF	GO:0015082	1.81322548	0.026644798	1.579586877	di-, tri-valent inorganic cation transmembrane transporter activity
MF	GO:0015267	1.562818683	0.012551231	2.187120292	channel activity

MF	GO:0015662	2.039783931	2.60E-04	2.065613609	ATPase activity, coupled to transmembrane movement of ions, phosphorylative mechanism
MF	GO:0017076	0.362399652	0.003413612	26.00243013	purine nucleotide binding
MF	GO:0022803	1.562818683	0.012551231	2.187120292	passive transmembrane transporter activity
MF	GO:0022804	0.929996543	0.041293348	4.374240583	active transmembrane transporter activity
MF	GO:0022838	1.580096674	0.010753402	2.187120292	substrate-specific channel activity
MF	GO:0022857	0.79510714	0.002745981	7.776427704	transmembrane transporter activity
MF	GO:0022891	0.958105226	3.31E-04	6.925880923	substrate-specific transmembrane transporter activity
MF	GO:0022892	0.883137525	8.73E-04	7.290400972	substrate-specific transporter activity
MF	GO:0030554	0.409747787	7.06E-04	24.90886999	adenyl nucleotide binding

MF	GO:0032553	0.414544354	3.86E-04	25.63791009	ribonucleotide binding
MF	GO:0032555	0.414544354	3.86E-04	25.63791009	purine ribonucleotide binding
MF	GO:0032559	0.462212099	7.42E-05	24.54434994	adenyl ribonucleotide binding
MF	GO:0042625	1.59654954	0.015306675	2.065613609	ATPase activity, coupled to transmembrane movement of ions
MF	GO:0046873	1.463283009	0.019407649	2.308626974	metal ion transmembrane transporter activity
BP	GO:0006754	1.515121941	0.044409183	2.065613609	ATP biosynthetic process
BP	GO:0009141	1.495704487	0.033186541	2.187120292	nucleoside triphosphate metabolic process
BP	GO:0009142	1.495704487	0.033186541	2.187120292	nucleoside triphosphate biosynthetic process
BP	GO:0009144	1.495704487	0.033186541	2.187120292	purine nucleoside triphosphate metabolic process

BP	GO:0009145	1.495704487	0.033186541	2.187120292	purine nucleoside triphosphate biosynthetic process
BP	GO:0009199	1.495704487	0.033186541	2.187120292	ribonucleoside triphosphate metabolic process
BP	GO:0009201	1.495704487	0.033186541	2.187120292	ribonucleoside triphosphate biosynthetic process
BP	GO:0009205	1.495704487	0.033186541	2.187120292	purine ribonucleoside triphosphate metabolic process
BP	GO:0009206	1.495704487	0.033186541	2.187120292	purine ribonucleoside triphosphate biosynthetic process
BP	GO:0045087	1.037018255	0.038395427	3.888213852	innate immune response
BP	GO:0046034	1.515121941	0.044409183	2.065613609	ATP metabolic process
CC	GO:0005741	2.548171907	0.018169086	0.85054678	mitochondrial outer membrane

Parameters

Table S2 full parameter settings for all tools

Analysis	Tool and version	Parameters
Adaptor trimming	Scythe (one version)	Default
Quality trimming	Sickle (one version)	-t sanger -q 20
Bionano scaffolding	IrisView (v2.5)	alignment 1e-8 refineAlignment 1e-9<br>hybrid scaffolding_config_aggressive'
Final contig assembly	Canu (v1.5)	corOutCoverage=200 "batOptions=-dg 3 -db 3 -dr 1 -ca 500 -cp 50"
Alignments for assembly validation	Bowtie2 (v2.3.3.1)	--very-fast
Polishing - PacBio reads	Quiver (Genomic Consensus v2.3.3)	Default
Polishing - Illumina reads	BWA mem (v0.7.17), Pilon (v1.21)	Default
Polishing – RnaSeq data	Pilon (v1.21)	Default
Polishing – kmer assesment	KAT comp (v2.3.4)	-m 32
Polishing – transcript assessment	BLAT (v3.4)	-minIdentity=95
SNP to assembly mapping (genetic map comparison)	BLAT (v3.4)	Default
Hi-C clustering	Lachesis (one version)	Simple config file instead of command line parameters. The only important setting really is the number of expected linkage groups (set to 17).
Illumina assembly	SOAPdeNovo2 (v2.04)	pregraph -d 1 -K 51 -R<br>contig -m 127 -M 0 -R
Repeat annotation	Repeat masker (v4.0.5), Repeat modeller (v1.0.8)	Default. With consensus HODOR sequence from NCBI added to the repeat library manually.
Transcriptome assembly	Trinity GG (v2.2.0), Trinity (v2.2.0), Cufflinks (v2.2.1), Evigene (one version)	Default
Gene annotation	Eugene (v4.2)	Extensive config file. Available on request. The config file amongst other things points to files describing manually annotated gene models.

Gene annotation	Augustus (v3.3)	Default parameters but again manually annotated gene models were used for training and hints files were produced by mapping transcripts with BLAT.
Gene annotation	Evidence Modeller (v1.1.1)	Default parameters
Gene annotation: Protein alignment	Genome threader (v1.7.0)	Default parameters
Gene annotation: Cdna alignment	Genome threader (v1.7.0)	Default parameters
Gene annotation: Cdna alignment	BLAT (v3.4)	-minIdentity=95
Gene annotation: Rna Seq alignment	HISAT2 (v2.1.0)	--min-intronlen 40 --max-intronlen 1000 -k 20
Gene annotation: Splice site modelling and prediction	SpliceMachine (v1.2)	Default
Gene annotation: ORF prediction	Transdecoder (v5.0.2)	Default
Gene annotation: Genome based transcriptome assembly	PASA (v2.3.1)	-I 1000 -aligners BLAT
K_S distributions: Gene family construction	BLASTP (v2.2.17)	blastall -p blastp -m 8 -e 1e-10
K_S distributions: Gene family construction	mcblastline (v10-201)	--blast-score=e --blast-m9 --mcl-I=3 --mcl-scheme=7 --ass-repeat=max
K_S distributions: Paralog alignment	MUSCLE (v3.8.31)	Default
K_S distributions: K_S estimation	CODEML (PAML v4.4c)	Default, except: runmode=-2, seqtype=1, CodonFreq=2, model=0, kappa = 1.5, omega = 0.1

K_S distributions: Gene family phylogenetic trees	PhyML (v20150219)	-d aa -f m -v e -a e -s BEST -o tlr
K_S distributions: one-to-one ortholog detection	InParanoid (v4.1)	Default
Orthology: alignments	BLASTP	Evalue < 10^{-4}
Othology: gene families	Orthofinder	Default
Gene synteny	MCScanX (one version)	-s 20
Gene synteny: duplication depth	dissect_multiple_alignment.pl (MCScanX) (one version)	Default
Inter species chromosome alignments	Minimap2 (v2.16)	-x asm10
Intra species chromosome alignments	Minimap2 (v2.16)	-x asm5
Dot plots	DGENIES (one version)	Default

Table S3 assembly parameter settings including non optimal assemblies

Assembler	Parameters	Num Contigs	Min Contig length (bp)	N50 (Mb)	Max Contig Length (Mb)	Assembly Size (Mb)
canu	default	1066	3524	3.07	1.24	503
canu	corMaxEvidenceErate=0.15	3506	1316	0.22	0.10	477.4
canu	-corOutCoverage=200 -correctedErrorRate=0.040 "batOptions= -dg 3 -db 3-dr 1 -ca 500 -cp 50"	620	1582	5.33	17.01	501
canu	-minReadLength=2000 -corOutCoverage=200 -correctedErrorRate=0.040 "batOptions=-dg 3 -db 3 -dr 1 -ca 500 -cp 50"	614	2851	5.42	17.01	500.8
canu	-corOutCoverage=200 minOverlapLength=1000 -minReadLength=2000 -corOutCoverage=200 -correctedErrorRate=0.040 "batOptions=-dg 3 -db 3 -dr 1 -ca 500 -cp 50"	612	2851	5.42	15.45	500.8
canu	-corOutCoverage=200 minOverlapLength=1750 -minReadLength=2000 -corOutCoverage=200 -correctedErrorRate=0.040 "batOptions=-dg 3 -db 3 -dr 1 -ca 500 -cp 50"	613	7128	4.77	15.45	500.3
canu	-corOutCoverage=200 minOverlapLength=2000 -minReadLength=2000 -corOutCoverage=200 -correctedErrorRate=0.040 "batOptions=-dg 3 -db 3 -dr 1 -ca 500 -cp 50"	628	4267	4.69	14.75	500
Falcon	length_cutoff_pr=1000	1144	582	3.02	8.57	507.4
Falcon	length_cutoff_pr=2000	1158	582	2.95	8.57	507.7
Falcon	length_cutoff_pr=3000	1153	582	2.88	8.71	508.2
Falcon	length_cutoff_pr=4000	1149	582	2.99	8.60	508.7
Falcon	length_cutoff_pr=5000	1136	582	2.69	14.18	509.1
Falcon	length_cutoff_pr=6000	1124	582	2.53	9.50	509.6
Falcon	length_cutoff_pr=7000	1282	533	2.46	8.61	512.5
Falcon	length_cutoff_pr=8000	1253	533	2.67	8.61	512.4
Falcon	length_cutoff_pr=9000	1227	533	2.52	10.07	512
Falcon	length_cutoff_pr=10000	1082	637	2.53	9.47	510.3
Falcon	length_cutoff_pr=11000	1087	533	2.47	11.67	510.1
Falcon	length_cutoff_pr=12000	1057	533	2.45	11.69	510
Falcon	length_cutoff_pr=13000	1043	533	2.55	8.79	509.6
Falcon	length_cutoff_pr=14000	1024	533	2.28	8.78	508.5
Falcon	length_cutoff_pr=15000	977	533	2.12	8.79	506.2
