# Supplementary material for: Pseudo-chromosome–length genome assembly of a double haploid “Bartlett” pear (Pyrus communis L.)
Source: Gigascience. 2019 Dec 9;8(12):giz138. doi: 10.1093/gigascience/giz138 (PMC6901071; doi:10.1093/gigascience/giz138)
Supplement: giz138_Response_to_Reviewer_Comments_Original_Submission [file giz138_response_to_reviewer_comments_original_submission.pdf]

Author's Response To Reviewer Comments

Close

Dear Editor,

We are thankful to the reviewers for their useful comments. We addressed them as described below and are confident these updates improved the quality of the manuscript, which we hope is now suitable for publication. In the following, we report the concern raised by the reviewer and our actions to improve the manuscript (we enabled the track changes feature to highlight the changes in the manuscript).

Responses to Reviewer #1

• Provide the number of duplicated BUSCOs in the genome.

We have added this information in the updated manuscript

• Determine the number of BUSCOs in the annotated gene set.

We have run Busco on the gene annotation as well as the assembly and we now provide the results in the 'Gene Annotation' section of the results and discussion

• What number and diversity of RNAseq datasets were used to annotate the genome?

The complete list of SRA identifiers are now listed in the Methods subsection 'Transcriptome Assembly'.

• Raw reads and data need to be deposited in NCBI or EMBL or DDBJ

The raw data is now deposited under the bioproject PRJNA555057. An entry for the data related to this manuscript has also already been set up in the GigaDB database.

• Provide a color scale for Figure 2 and associated supplemental figures

This has been done in the updated manuscript

• Figure 4. In between Malus and domestica there is a box instead of an "x"

This has been fixed in the updated manuscript

• Telomeres is frequently misspelled.

We have corrected this misspelling mistake in the updated manuscript

• I am unclear what is meant in this sentence "Inter-assembly synteny is very strong, suggesting that although highly fragmented, the Bartlettv1.0 assembly was a veridical depiction of the genome". What is verdical? Do you mean vertical? If so, what does that mean?

We are sorry about this not so clear sentence. Verdical is a misspelling of veridical. What we meant here is that the Bartlettv1.0 genome was indeed a genuine representation of the genome. To make this more clear, we replaced “veridical” with “true” in the updated manuscript.

Responses to Reviewer #2

• Review Summary: Overall the paper reads well and describes an improved genome assembly for the European pear. The improvements come from using a mix of data sources: long read assemblies polished with short read data; optical and genetic maps and Hi-C data for scaffolding; and RNA-seq data

to improve annotations. This is an appropriate and detailed set of technologies to use for a genome assembly. The specimen chosen is a double haploid, which makes assembly more tractable, in comparison to the heterozygous specimen used for the first European pear assembly (I think that this should be mentioned in the Abstract). The paper's organisation and language used are clear. The work is discussed in context of previous and current research on pear and apple genomes; relevant literature is cited. I commend the authors for this large detailed study and the improved assembly of this important species. Additional review comments and suggestions are below, mainly: some methods need a more detailed description.

We thank the reviewer for the appreciation of our efforts. We added the following sentences to the abstract to emphasize the heterozygous nature of the original European pear assembly: "The sample selected for sequencing is a doubled haploid derived from the same 'Bartlett' pear that was sequenced for the existing P. communis reference. Sequencing of di-haploid plants makes assembly more tractable in highly heterozygous species such as P. communis".

• Data availability: Raw (genomic) sequencing data is provided in the supplementary data; however, for Illumina reads, I can only see the R2 (not R1) file. SRR accession numbers are provided for RNA-seq data. The link to the genome assembly does not work; but this does:
https://www.rosaceae.org/organism/Pyrus/communis

We are sorry about the link that did not work. The administrators of the GDR changed the URL shortly after our initial submission to bioRxiv. We double checked the link and put the correct URL in the revised manuscript. The correct address is:
https://www.rosaceae.org/species/pyrus/pyrus_communis/genome_v2.0
As already mentioned to Reviewer #1, the raw data has now been uploaded to SRA (bioproject PRJNA555057) and an entry to GigaDB has also been set up.

• Detail of how tools were used: Tool versions have usually been provided but please provide the parameters used so that analyses are reproducible. GigaScience also recommends listing whether tools have an open source license.

We amended the text and tool versions are now listed where the tool is versioned. Some of the tools (for example ALE) simply have a single version that was released on publication. A parameter settings section has now been added to the Supplementary material. At the beginning of the Material and Methods section we added the following sentence to refer to this table: "For the ease of reading. software versions (where applicable) and complete set of parameters for each tool used in the analysis have been reported in Supplementary Table S2."

• Read QC: Please describe if any QC, trimming, or filtering was taken for raw reads.

This is an omission on our part and Illumina read trimming has now been added to the methods section. The following sentences were added to the Section Sample preparation and sequencing: "Illumina adaptor sequences were clipped using the Scythe software. Illuimina sequences were trimmed at their 3' end where the average quality within a 25bp window fell below Q20 using the Sickle software." PacBio read trimming is part of canu's read correction algorithm. Canu goes through explicit trimming, correction, and assembly phases.

• Assembly: method: manuscript says the description is in the Supplementary Methods, but I can't see the relevant file(s) in the downloaded "assembly-results" folder. This information is important and should be included.

We believe the reviewer is referring to this sentence. "For each assembler the most important assembly parameters were systematically varied (Supplementary Methods), as defined by the tool developers, and by consideration of assembly theory (e.g. overlap length, overlap identity for overlap layout consensus assembly)."
The reviewer is quite correct, and these details were not in the original Supplementary methods. Following the reviewer's suggestion, we have now included this detail in the main text instead of in the supplementary methods.

• Kmer analysis of Illumina reads: This is discussed in the first and last paragraphs of Results, and in Assembly Polishing; Figures 1a, 1b, 8, 9. Please provide more detail on tool settings used for KAT and a brief justification for choice of kmer size 17. Figures 8 and 9 show which kmers are shared between

reads and the assemblies, noting that some kmers in the "homozygous" peak appear twice or three times in the assembly (and this fraction has been used to assess each iteration of Pilon assembly polishing).

The reviewer raises a number of points here (since KAT analysis is used in more than one place in our study) and we will address them separately below.

Kmer size of 17. This is actually an omission from the methods, since different kmer sizes have been used for different purposes, with kmer size appropriate for each analysis being selected. A kmer size of 17 was used for the heterozygosity (Fig 1a and 1b) and assembly duplication plots (Figs 8 and 9 now Figs S36 and S37), however, a larger kmer size (of 32) was used for the assessment of Pilon assembly polishing. We originally did not provide the kmer size used for analysis of polishing, and we have now added that to the manuscript.

A kmer size of 17 was chosen for the heterozygosity and assembly duplication plot because it clearly demonstrates the peaks in the spectra. At a larger kmer sizes, (more specific) there are less pronounced differences between the heterozygous and homozygous peaks, since most kmers will fall into only one of the two haplotypes. The same applies to duplications in the genome. These are clearer at a smaller kmer size but at large kmer sizes everything just becomes unique. This is also a function of the error rate of the data. Every error introduces k error kmers into the analysis. Higher quality data will support this type of analysis at larger kmer sizes. We have actually done these analyses at a range of kmer sizes on both raw and corrected data. We provided plots at k = 17 basically because they are the most visually striking in making our point about high heterozygosity vs. low (if any) residual heterozygosity. Any residual heterozygosity in the di-haploid would be visually clearer to detect at a low value of k.

A larger kmer size is used for analysing the polishing because it is more specific and a kmer of 32 is used since this is the largest kmer which can be stored as a 16 bit integer. So it is for reasons of computational efficiency that we don't use an even larger kmer. The GenomeScope authors recommend 27 as a default, most likely because they are using one number to cover different types of analysis.

• However it is my understanding that true repeats may appear in this homozygous peak, if they are from a duplicated heterozygous sequence (e.g. as per the GenomeScope paper supplement, page 7). If so, consider if this affects any conclusions drawn? Consider also including log scale plots if possible to more clearly see all the peaks.

On the interpretation of the plots the reviewer opens an interesting discussion. If we introduce a terminology whereby we referred to the possible 'allelles' of a repeat as AA-AA, AA-BB, AB-AB, AB-BB then we believe the reviewer is referring to AB-AB kmers from repeat regions. These would then be kmers from heterozygosity that preceded the whole genome duplication. The reviewer is correct that these kmers would fall into the main peak (ABAB and AABB are not distinguishable). If there were a lot of these regions then this could indeed affect the conclusion that significant sequence is erroneously duplicated in the asian pear assembly.

Another possible interpretation of these figures which we have been offered since submission of the manuscript, is that these regions which appear duplicated in the asian pear assembly are in fact substitution of sequence from one copy of an imperfect repeat to another. This explanation would also account for the large amount of content which is missing from the assembly. Under this explanation, content has essentially been transferred from the black coloured regions of the main peak to the green and purple coloured regions.

Since we can provide three possible interpretations of these figures and the reality is likely to represent a mixture of these three scenarios, we've decided to move these figures to the supplementary, where we briefly speculate on all three of these possibilities. In the main text we focus on the genome we assembled, the european pear di-haploid.

In addition we have included more information about the exact parameters etc. used for KAT analysis as per the reviewer's suggestion.

• Relationship between genetic and physical distance: Marey maps in supplementary figures S2-S18: Please provide more detail on which software was used to create these plots. The manuscript text says that these maps show "good agreement between the assembly and the Bartlett genetic map"; however there seems to be no mention of the points that don't fit this pattern (e.g. Figure S3). For the plots:

Label axes to ends. What does it mean if a linkage group does not follow the particular relationship between physical chromosome position and actual sequence length distance? Are the blue and orange colours shared between plots or only to distinguish an alternate linkage group within a single plot? Figure S2 - the dot point in the top right hand corner could be easily missed - would suggest not boxing in the plot.

As the reviewer correctly reported, marey maps simply represent the relationship between physical and genetic distance and provide an additional proof of the correctness of the assembled sequence by using independent data (i.e. not used in the assembly process). These simple plots can be made with any data plotting toolkit. In our case, we plotted the data using python and matplotlib. We did not report this because we thought the specific tool used was not relevant due to the lack of any specific analysis. To comply with the reviewer request, we added the specification of the usage of python's matplotlib v.3.0.3 to produce the plot. One of the plots had a different color (orange instead of blue) and this could be confusing the reader. Therefore we re-created the plots using consistent colors and also added a legend for the heat maps.
We agree with the reviewer that there are a few minor local inconsistencies between the physical and genetic positions but these might be due to the resolution of the genetic map used, which is limited by the number of progenies available (100 individuals) and from the fact that this Bartlett parental map was obtained from an integrated map (Bartlett x Old Home).
Nevertheless, these plots represent an independent assessment of the overall quality of the assembled sequence.

• Heatmaps: For the heatmaps in Figure 2 and Figures S3-S18 please describe in the manuscript text how these were obtained (method, software).

Heatmaps are produced by binning the count of the interesting features (i.e. genes or dispersed repeats) in bins of 200,000 base pairs. As in the case above, we did not use any specific software to do this, we just plotted the information with python's matplotlib v.3.0.3.

• Figure 3. I find this plot hard to read, both the font size and the layout. Is there an alternative way to show these results, perhaps with colour? The caption would benefit from re-wording for clarity.

We have replaced Figure 3 by plotting the orthology overlap information in an alternative 'rugby ball' format which we believe is much easier to read.

• Figure 5. Not referred to in manuscript.

We included Figure 5 as an example of the alignment figures shown in the Supplementary methods. In fact it is repeated as Figure S18. We have now made this clear in the text. We added the following sentences to the section Chromosome Structure: "Figure 5 is included as an example of the intra-species and intra-assembly alignments showing chromosome 1. Alignments for all 17 chromosomes are given in Supplementary figures S18 to S34".

• Table 4: The caption should explain where the statistics are from. I would also suggest adding the references for these genome assemblies.

We have added these references to the figure caption, also to the caption of Figure 3. We also recomputed these figures after removing UTRs from the GFF annotations, since annotation of UTR is difficult and we realized it is inconsistently implemented across these different studies.

• References: Consider including Wu et al. 2018 "Diversification and independent domestication of Asian and European pears" to put P. communis in context with genomics from 33 other Pyrus species.

We thank the reviewer for this suggestion. We took it on board now and added "There currently exists resequencing data from 113 pear accession representing both cultivated and wild pear species, but as yet no chromosome scale reference genome has been published" as the final sentence in the introduction, citing the Wu et al. paper.

We have also added this reference:
Watson, M. & Warr, A. Errors in long-read assemblies can critically affect protein prediction. Nat. Biotechnol. 37, 124–126 (2019).

This paper is cited at the following sentence “The rationale for this was that small errors are particularly a problem in coding regions because they can introduce frameshifts that severely affect the annotation of genes’

• Supplementary Figure 1: I find the caption wording a bit unclear; would it be better to use the term "haplotypes" and not "alleles"; and further note that these are the two parental haplotypes in the double haploid sample?

We agree with the reviewer and we made the suggested change.

• Supplementary Table S1: Add more information to caption about how this information was obtained.

Please see comment above on marey maps. We amended the caption of these supplementary figures as discussed above.

• Other: define acronyms and unusual words at first use, e.g. LG; paranome.

Thanks for pointing this out, we have fixed this in the text.

On behalf of all the authors.
Luca Bianco, Michela Troggio, Yves Van De Peer

Close
