# Supplementary material for: Pseudo-chromosome–length genome assembly of a double haploid “Bartlett” pear (Pyrus communis L.)
Source: Gigascience. 2019 Dec 9;8(12):giz138. doi: 10.1093/gigascience/giz138 (PMC6901071; doi:10.1093/gigascience/giz138)
Supplement: giz138_Response_to_Reviewer_Comments_Revision_1 [file giz138_response_to_reviewer_comments_revision_1.pdf]

Author's Response To Reviewer Comments

Close

Dear Editor,

As requested, track changes have been disabled and references updated.

Close
