# Supplementary material for: Pseudo-chromosome–length genome assembly of a double haploid “Bartlett” pear (Pyrus communis L.)
Source: Gigascience. 2019 Dec 9;8(12):giz138. doi: 10.1093/gigascience/giz138 (PMC6901071; doi:10.1093/gigascience/giz138)
Supplement: giz138_Reviewer_1_Report_Original_Submission — C Robin Buell -- 7/31/2019 Reviewed [file giz138_reviewer_1_report_original_submission.pdf]

Reviewer Report

Title: Pseudo-chromosome length genome assembly of a double haploid 'Bartlett' pear (Pyrus communis L.)

Version: Original Submission **Date:** 7/31/2019

Reviewer name: C Robin Buell

Reviewer Comments to Author:

This is a well done set of genome analyses. The manuscript is well written and the datasets are convincing that the final assembly represents a solid, well annotated set of gene models. A few minor suggestions to consider:
Provide the number of duplicated BUSCOs in the genome.
Determine the number of BUSCOs in the annotated gene set.
What number and diversity of RNAseq datasets were used to annotate the genome?
Raw reads and data need to be deposited in NCBI or EMBL or DDBJ
PRovide a color scale for Figure 2 and associated supplemental figures
Figure 4. In between Malus and domestica there is a box instead of an "x"
Telomeres is frequently misspelled.
I am unclear what is meant in this sentence "Inter-assembly synteny is very strong, suggesting that although highly fragmented, the Bartlettv1.0 assembly was a veridical depiction of the genome". What is verdical? Do you mean vertical? If so, what does that mean?

Level of Interest

Please indicate how interesting you found the manuscript: Choose an item.

Quality of Written English

Please indicate the quality of language in the manuscript: Choose an item.

Declaration of Competing Interests

Please complete a declaration of competing interests, considering the following questions:

- Have you in the past five years received reimbursements, fees, funding, or salary from an organisation that may in any way gain or lose financially from the publication of this manuscript, either now or in the future?
- Do you hold any stocks or shares in an organisation that may in any way gain or lose financially from the publication of this manuscript, either now or in the future?
- Do you hold or are you currently applying for any patents relating to the content of the manuscript?

- Have you received reimbursements, fees, funding, or salary from an organization that holds or has applied for patents relating to the content of the manuscript?
- Do you have any other financial competing interests?
- Do you have any non-financial competing interests in relation to this paper?

If you can answer no to all of the above, write 'I declare that I have no competing interests' below. If your reply is yes to any, please give details below.

I declare that I have no competing interests

I agree to the open peer review policy of the journal. I understand that my name will be included on my report to the authors and, if the manuscript is accepted for publication, my named report including any attachments I upload will be posted on the website along with the authors' responses. I agree for my report to be made available under an Open Access Creative Commons CC-BY license (http://creativecommons.org/licenses/by/4.0/). I understand that any comments which I do not wish to be included in my named report can be included as confidential comments to the editors, which will not be published.

Choose an item.

To further support our reviewers, we have joined with Publons, where you can gain additional credit to further highlight your hard work (see: https://publons.com/journal/530/gigascience). On publication of this paper, your review will be automatically added to Publons, you can then choose whether or not to claim your Publons credit. I understand this statement.

Yes Choose an item.
