# Supplementary material for: Pseudo-chromosome–length genome assembly of a double haploid “Bartlett” pear (Pyrus communis L.)
Source: Gigascience. 2019 Dec 9;8(12):giz138. doi: 10.1093/gigascience/giz138 (PMC6901071; doi:10.1093/gigascience/giz138)
Supplement: giz138_Reviewer_2_Report_Original_Submission — Anna Syme -- 8/7/2019 Reviewed [file giz138_reviewer_2_report_original_submission.pdf]

Reviewer Report

Title: Pseudo-chromosome length genome assembly of a double haploid 'Bartlett' pear (Pyrus communis L.)

Version: Original Submission **Date:** 8/7/2019

Reviewer name: Anna Syme

Reviewer Comments to Author:

Review of "Pseudo-chromosome length genome assembly of a double haploid 'Bartlett' pear (Pyrus communis L.)"

Review Summary: Overall the paper reads well and describes an improved genome assembly for the European pear. The improvements come from using a mix of data sources: long read assemblies polished with short read data; optical and genetic maps and Hi-C data for scaffolding; and RNA-seq data to improve annotations. This is an appropriate and detailed set of technologies to use for a genome assembly. The specimen chosen is a double haploid, which makes assembly more tractable, in comparison to the heterozygous specimen used for the first European pear assembly (I think that this should be mentioned in the Abstract). The paper's organisation and language used are clear. The work is discussed in context of previous and current research on pear and apple genomes; relevant literature is cited. I commend the authors for this large detailed study and the improved assembly of this important species. Additional review comments and suggestions are below, mainly: some methods need a more detailed description.

Data availability: Raw (genomic) sequencing data is provided in the supplementary data; however, for Illumina reads, I can only see the R2 (not R1) file. SRR accession numbers are provided for RNA-seq data. The link to the genome assembly does not work; but this does: https://www.rosaceae.org/organism/Pyrus/communis

Detail of how tools were used: Tool versions have usually been provided but please provide the parameters used so that analyses are reproducible. GigaScience also recommends listing whether tools have an open source license.

Read QC: Please describe if any QC, trimming, or filtering was taken for raw reads.

Assembly: method: manuscript says the description is in the Supplementary Methods, but I can't see the relevant file(s) in the downloaded "assembly-results" folder. This information is important and should be included.

Kmer analysis of Illumina reads: This is discussed in the first and last paragraphs of Results, and in Assembly Polishing; Figures 1a, 1b, 8, 9. Please provide more detail on tool settings used for KAT and a brief justification for choice of kmer size 17. Figures 8 and 9 show which kmers are shared between reads and the assemblies, noting that some kmers in the "homozygous" peak appear twice or three times in the assembly (and this fraction has been used to assess each iteration of Pilon assembly polishing). However it is my understanding that true repeats may appear in this homozygous peak, if they are from a duplicated heterozygous sequence (e.g. as per the GenomeScope paper supplement,

page 7). If so, consider if this affects any conclusions drawn? Consider also including log scale plots if possible to more clearly see all the peaks.
Relationship between genetic and physical distance: Marey maps in supplementary figures S2-S18: Please provide more detail on which software was used to create these plots. The manuscript text says that these maps show "good agreement between the assembly and the Bartlett genetic map"; however there seems to be no mention of the points that don't fit this pattern (e.g. Figure S3). For the plots: Label axes to ends. What does it mean if a linkage group does not follow the particular relationship between physical chromosome position and actual sequence length distance? Are the blue and orange colours shared between plots or only to distinguish an alternate linkage group within a single plot?
Figure S2 - the dot point in the top right hand corner could be easily missed - would suggest not boxing in the plot.
Heatmaps: For the heatmaps in Figure 2 and Figures S3-S18 please describe in the manuscript text how these were obtained (method, software).
Figure 3. I find this plot hard to read, both the font size and the layout. Is there an alternative way to show these results, perhaps with colour? The caption would benefit from re-wording for clarity.
Figure 5. Not referred to in manuscript.
Table 4: The caption should explain where the statistics are from. I would also suggest adding the references for these genome assemblies.

References: Consider including Wu et al. 2018 "Diversification and independent domestication of Asian and European pears" to put P. communis in context with genomics from 33 other Pyrus species.
Supplementary Figure 1: I find the caption wording a bit unclear; would it be better to use the term "haplotypes" and not "alleles"; and further note that these are the two parental haplotypes in the double haploid sample?
Supplementary Table S1: Add more information to caption about how this information was obtained.
Other: define acronyms and unusual words at first use, e.g. LG; paranome.

Level of Interest

Please indicate how interesting you found the manuscript: Choose an item.

Quality of Written English

Please indicate the quality of language in the manuscript: Choose an item.

Declaration of Competing Interests

Please complete a declaration of competing interests, considering the following questions:

- Have you in the past five years received reimbursements, fees, funding, or salary from an organisation that may in any way gain or lose financially from the publication of this manuscript, either now or in the future?
- Do you hold any stocks or shares in an organisation that may in any way gain or lose financially from the publication of this manuscript, either now or in the future?
- Do you hold or are you currently applying for any patents relating to the content of the manuscript?
- Have you received reimbursements, fees, funding, or salary from an organization that holds or has applied for patents relating to the content of the manuscript?
- Do you have any other financial competing interests?
- Do you have any non-financial competing interests in relation to this paper?

If you can answer no to all of the above, write 'I declare that I have no competing interests' below. If your reply is yes to any, please give details below.

I declare that I have no competing interests.

I agree to the open peer review policy of the journal. I understand that my name will be included on my report to the authors and, if the manuscript is accepted for publication, my named report including any attachments I upload will be posted on the website along with the authors' responses. I agree for my report to be made available under an Open Access Creative Commons CC-BY license (http://creativecommons.org/licenses/by/4.0/). I understand that any comments which I do not wish to be included in my named report can be included as confidential comments to the editors, which will not be published.

Choose an item.

To further support our reviewers, we have joined with Publons, where you can gain additional credit to further highlight your hard work (see: https://publons.com/journal/530/gigascience). On publication of this paper, your review will be automatically added to Publons, you can then choose whether or not to claim your Publons credit. I understand this statement.

Yes Choose an item.
